# Asymmetric Total Synthesis of C2-OH Lycopodium Alkaloids (−)-Palhinine B, (−)-Palhinine C, and (+)-Palhinine B Enabled by Stereocontrolled Diels–Alder Strategy

**DOI:** 10.1021/acs.orglett.6c01704

**Published:** 2026-06-15

**Authors:** Amit Pantawane, Chih-Ming Chen, Tung-Chun Kuo, Julakanti Satyanarayana Reddy, Mu-Jeng Cheng, Shun-Yuan Luo, Hsing-Pang Hsieh

**Affiliations:** † Institute of Biotechnology and Pharmaceutical Research, 50115 National Health Research Institutes, Miaoli County 350, Taiwan, ROC; ‡ Department of Chemistry, 34916 National Chung Hsing University, Taichung 402, Taiwan, ROC; § Department of Chemistry, 34912 National Cheng Kung University, Tainan 701, Taiwan, ROC; ∥ Department of Chemistry, National Tsing Hua University, Hsinchu 300, Taiwan, ROC

## Abstract

The first asymmetric total synthesis of *Lycopodium* alkaloids bearing a 1,2-amino alcohol motif, including (−)-palhinine B, (−)-palhinine C, and (+)-palhinine B, has been achieved. The synthesis features early stage installation of a chiral C2-OH group from a tyrosine-derived 1,2-amino alcohol, which governs the overall stereochemical outcome at C7, C13, C15, and a quaternary stereogenic center at C12 of the tetracyclic palhinine skeleton. A highly diastereoselective Diels–Alder reaction of a masked *ortho*-benzoquinone, followed by a thiol-mediated acyl radical cyclization, enables rapid construction of the isotwistane core. DFT-guided analysis revealed the challenge of late-stage C2-OH inversion and led to a successful chemo- and diastereoselective reduction of a caged hemiaminal ketone, providing access to the C2 epimer. This work establishes a unified asymmetric strategy for complex *Lycopodium* alkaloids and highlights the integration of computation with synthetic design.

## Graphical abstract

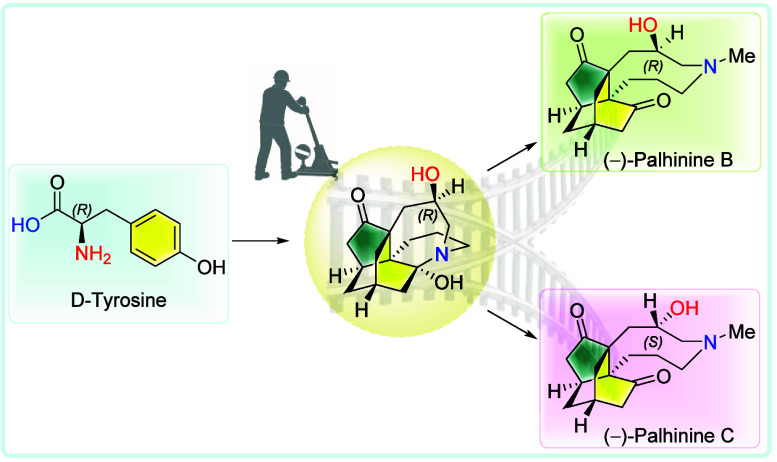

The *Lycopodium* alkaloids constitute a broad class of naturally occurring compounds with unique structures. Owing to their distinctive polycyclic structures and diverse biological functions, these alkaloids have attracted considerable interest from the synthetic community as both inspiration and challenging targets. Among them, the palhinine alkaloids, including palhinine B (**1**) and palhinine C (**2**), feature a densely fused azonane ring attached to an isotwistane (tricyclo­[4.3.1.0^3,7^]) skeleton (Figure 1a) bearing multiple contiguous stereocenters.^1^ Their intricate stereochemical arrangement, particularly around the 1,2-amino alcohol motif, distinguishes these natural products from other *Lycopodium* alkaloids and presents significant challenges to stereocontrolled synthesis.^2–5^ In several *Lycopodium* alkaloids, the 1,2-amino alcohol motif is a prevalent structural element that plays a crucial role in defining both molecular architecture and biological activity (Figure 1b).^6^ To the best of our knowledge, no total synthesis of *Lycopodium* alkaloids bearing a 1,2-amino alcohol group has been reported to date. Although the total syntheses of 1,3-amino alcohol motif-containing alkaloids palhinine A (**3**), palhinine D (**4**), and isopalhinine A (**5**) have been reported, a unified and asymmetric total synthesis of these alkaloids remains undeveloped.^4,5^ Motivated by our interest in the total synthesis of complex natural products, we set out to accomplish the first asymmetric total synthesis of C2-hydroxy (−)-palhinine B (**1**), its enantiomer (+)-palhinine B (*ent*-**1**), and its epimer (−)-palhinine C (**2**). To establish the absolute stereochemistry at an early stage, our strategy employed a chiral pool tyrosine-derived 1,2-amino alcohol, which also guided the desired configuration in the subsequent diastereoselective Diels–Alder reaction. Previous synthetic studies of related alkaloids have shown that the late-stage flip of the C2-hydroxy group is challenging.^4^ Therefore, DFT calculations were used to predict the outcome of the reduction of a C2-keto palhinine B intermediate, as shown in Figure 2.

**Figure 1:**
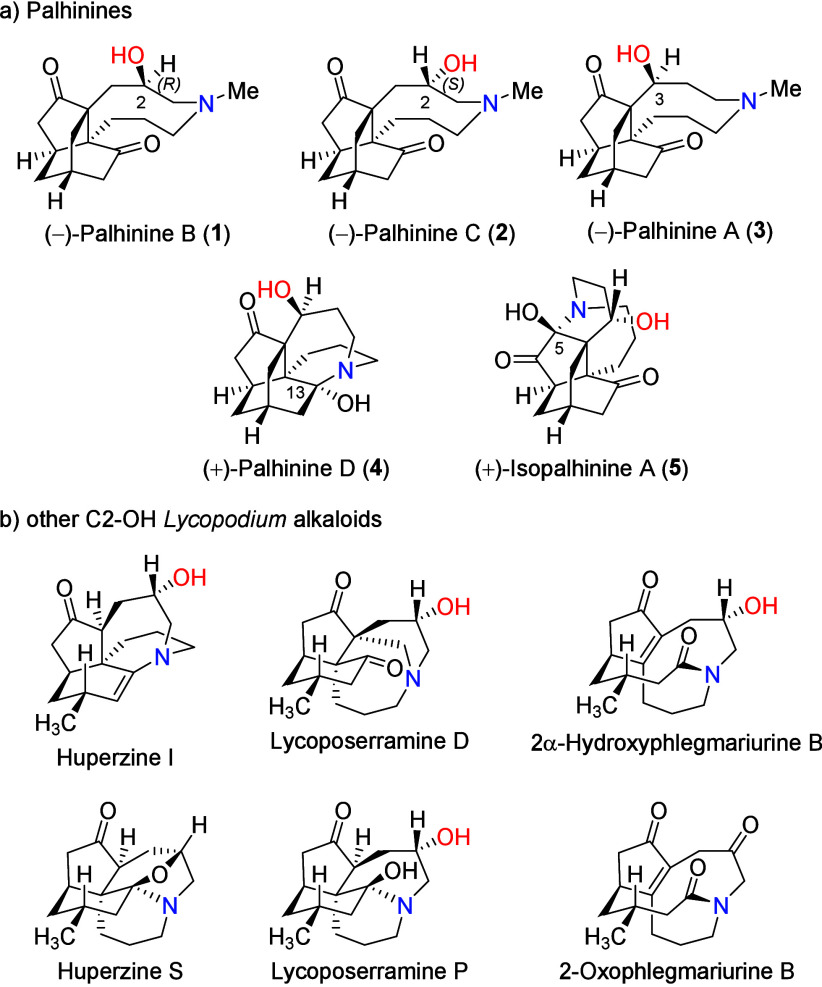
a) Palhinine alkaloids. b) Other C2-oxygenated *Lycopodium* alkaloids.^6^

**Figure 2:**
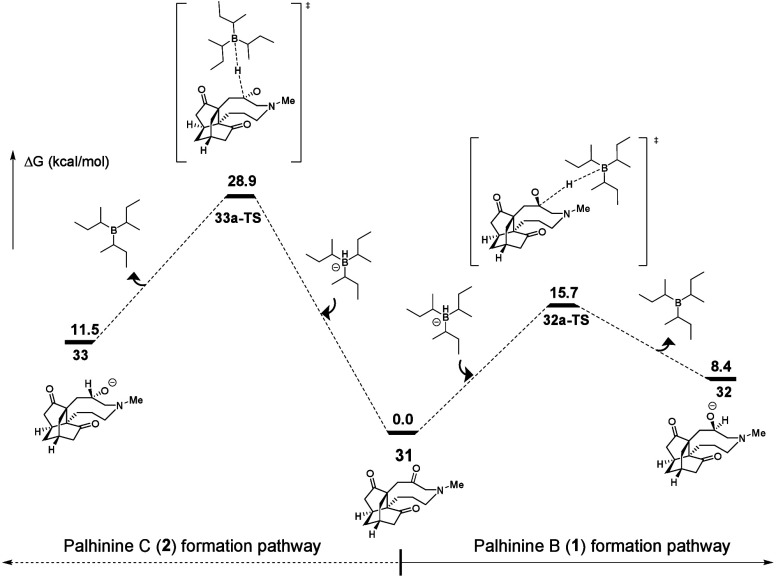
DFT calculations for the reduction of C2-carbonyl.

The retrosynthetic analysis is outlined in Scheme 1. To address the issue of transannular strain, our strategy involves constructing the nine-membered azonane ring first, followed by formation of the isotwistane skeleton.^5^ Based on our understanding of palhinine’s structures,^5^ we anticipated that palhinine B (**1**) could be accessed through a strategically designed Diels–Alder reaction with masked *ortho*-benzoquinone (MOB) **8**. This approach enables the stereoselective formation of the bridged tricyclic skeleton **6** with the desired endo configuration, while installing the chiral centers at C7, C15, and the quaternary center at C12 in a single step, thereby defining the palhinine framework. The MOB precursor **9**, containing a C2-OH azonane ring, could be synthesized from tyrosine (**10**). The C2-amine of tyrosine would be converted to a hydroxy group with retention of configuration. A sequence of transformations, including Friedel–Crafts reaction/Baeyer–Villiger oxidation to install the *ortho*-phenol, Claisen rearrangement, hydroboration oxidation, and subsequent S_N_2 cyclization, was employed to construct the azonane ring in **9**. Oxidative dearomatization of **9** would then provide MOB **8**. A Diels–Alder reaction with methyl acrylate would afford **7**. From intermediate **7**, the isotwistane core could be formed via the construction of the C4–C5 bond through a thiol-mediated acyl radical cyclization between an aldehyde and an alkene. Subsequent deprotection of isotwistane **6** to give the caged intermediate **25**, followed by N-methylation, would furnish palhinine B (**1**).

**Scheme 1:**
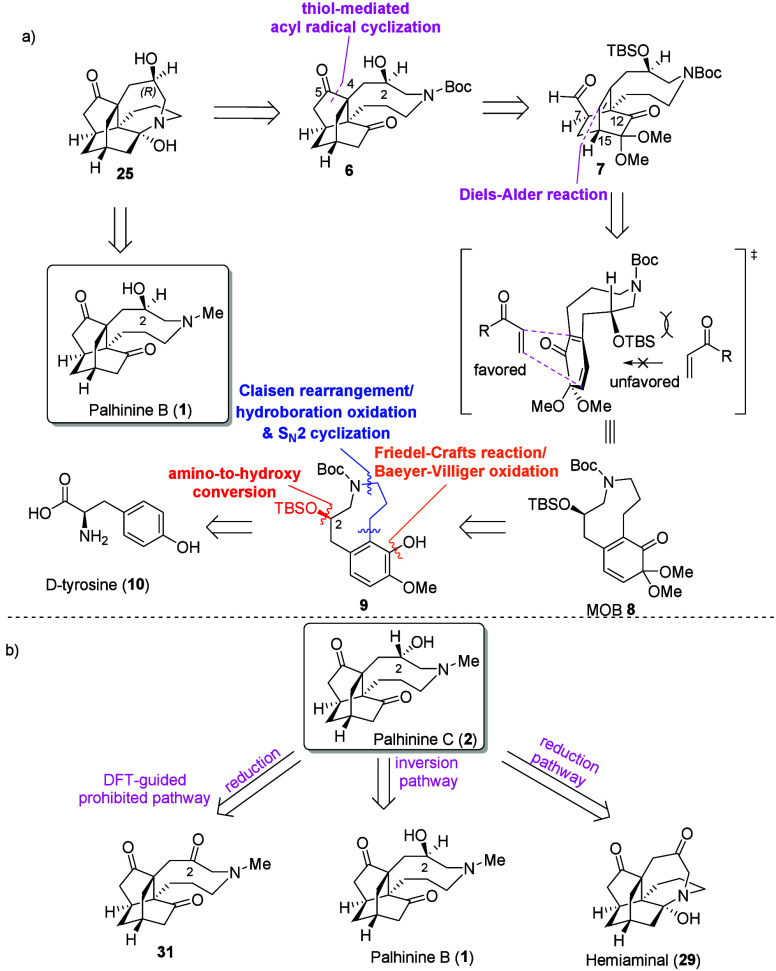
a) Retrosynthetic Analysis of Palhinine B (**1**) from d-Tyrosine (**10**). b) Retrosynthetic Analysis of Palhinine C (**2**)

A late-stage inversion of the C2-hydroxy group to access palhinine C (**2**) is expected to be challenging. Therefore, DFT calculations were performed to predict the outcome of the reduction. According to Fan’s results, a sterically demanding hydride reagent approaches the less hindered face of the rigid polycyclic framework during the late-stage reduction of the C3 ketone, leading to facially selective formation of the hydroxy group.^4^ Based on these findings, we deduced that the conversion of palhinine B (**1**) to palhinine C (**2**) through an oxidation–reduction pathway would be difficult, which is consistent with our computational results. Focusing on the key hydride attack by L-Selectride, our DFT calculations indicate that the transition state leading to the experimentally favored product via intermediate **32** has an activation barrier of 15.7 kcal/mol, whereas formation of palhinine C via intermediate **33** requires a significantly higher barrier of 28.9 kcal/mol (Figure 2). This substantial energy difference suggests that formation of palhinine C through this pathway is kinetically unfavorable, effectively ruling out this strategy. Accordingly, alternative strategies such as direct inversion or reduction of a hemiaminal ketone intermediate were considered (see Scheme 1b).

As shown in Scheme 2, the C2-OH group of carboxylic acid **11** was prepared from tyrosine (**10**) with retention of configuration via a known two-step procedure.^7^ After installing the C2-OH group, the α-hydroxy carboxylic acid **11** was protected using 2,2-dimethoxypropane (2,2-DMP), and the phenol was methylated. Subsequent Baeyer–Villiger oxidation afforded the acetylated intermediate **13**. Hydrolysis of the acetyl ester under basic conditions also cleaved the dioxolanone moiety, and reprotection furnished 2-methoxyphenol **14**. Phenolic allylation followed by Claisen rearrangement provided allylbenzene **15**, and subsequent removal of the dioxolanone with ammonia delivered amide **16**.^10a^ An alternative route to avoid repetitive protection steps involved hydrolysis using ammonia, followed by selective allylation to afford allyl ether **17** and subsequent Claisen rearrangement to give intermediate **16**. After protection of both hydroxy and phenol groups to furnish silyl ether **18**, reduction of the primary amide without affecting the alkene proved challenging. Classical LAH reduction resulted in undesired deprotection of the C2-hydroxy TBS group. The BEt_3_-catalyzed amide reduction with phenylsilane reported by Huang *et* *al*. led to the simultaneous reduction of both the amide and alkene, affording an inseparable mixture (see Scheme 2).^10b^ To overcome this issue, a one-pot sequential process was developed, involving hydroboration/amide reduction, borane oxidation, and N-Boc protection, which delivered primary alcohol intermediate **19**. This intermediate was then converted to mesylate **20**. Subsequent S_N_2 reaction using excess sodium hydride in THF, followed by quenching with methanol to selectively remove the phenolic silyl ether, afforded the desired MOB precursor **9** with (90% ee for **9** and 94% ee for ***ent*** **-9**) on a gram scale.

**Scheme 2:**
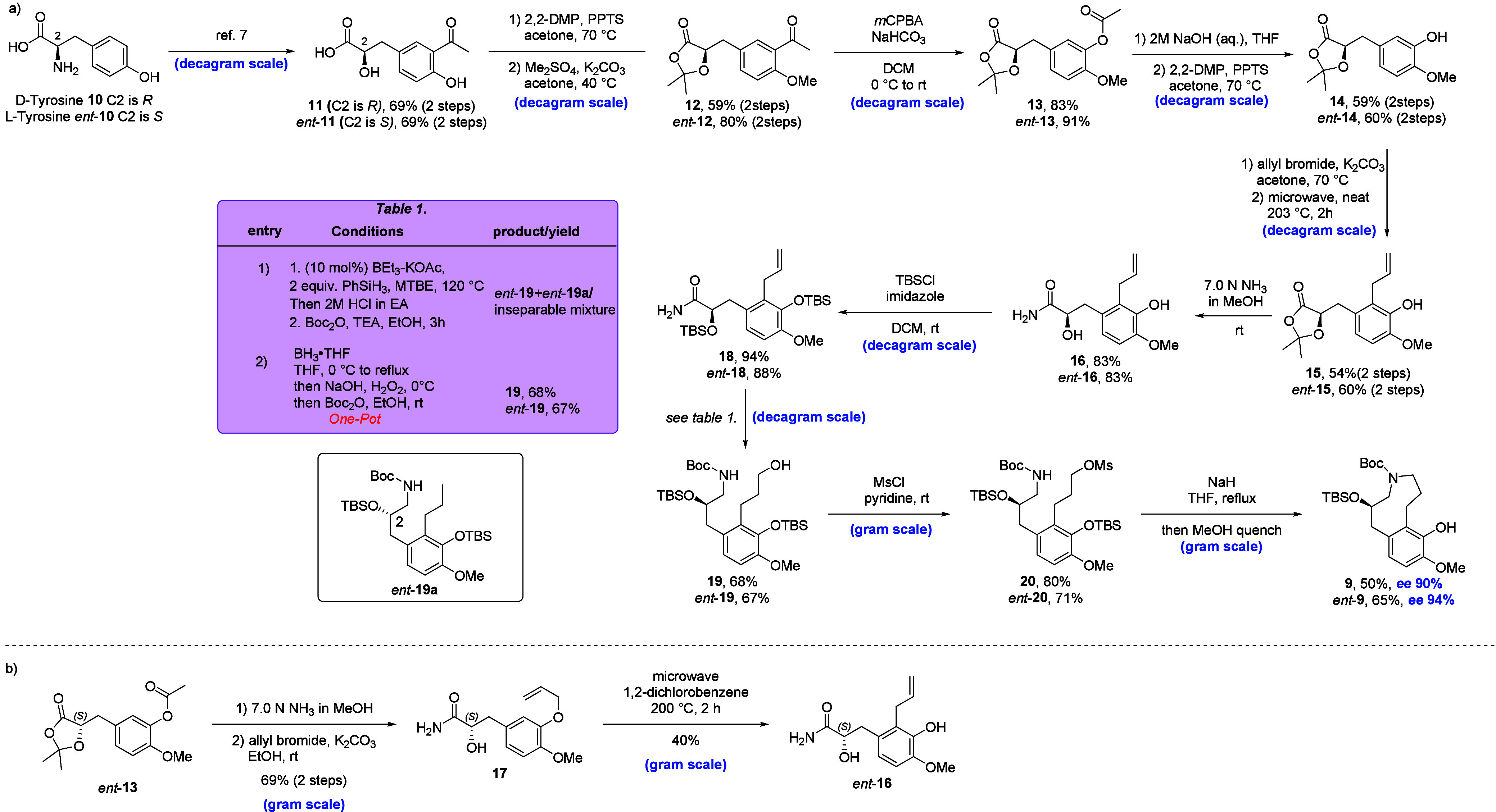
a) Synthesis of 2-Methoxyphenol with C2-OH Azonane **9** Using Tyrosine. b) Alternative Route for the Synthesis of *ent*-**16**

We next investigated the selectivity of the Diels–Alder reaction to construct the desired 6/6/9 tricyclic skeleton with the correct relative configuration of C2, C7, C12, and C15. In our previous work, the Diels–Alder reaction between C3-OH MOB intermediate and acrolein proceeded with a selectivity of 3:1.^5^ However, when MOB **8** (see Scheme 1a) was generated from **9** and treated with acrolein, in situ, the desired Diels–Alder adduct **7** was obtained only in trace amounts, as shown in Scheme 3. To improve both stability and selectivity, a bulkier dienophile, methyl acrylate, was examined. Gratifyingly, the Diels–Alder adduct **21** was obtained as a single product in 68% yield. This step efficiently established the key stereocenters at C7, C15, and a quaternary center at C12 in a single step on a gram scale.^8^

**Scheme 3:**
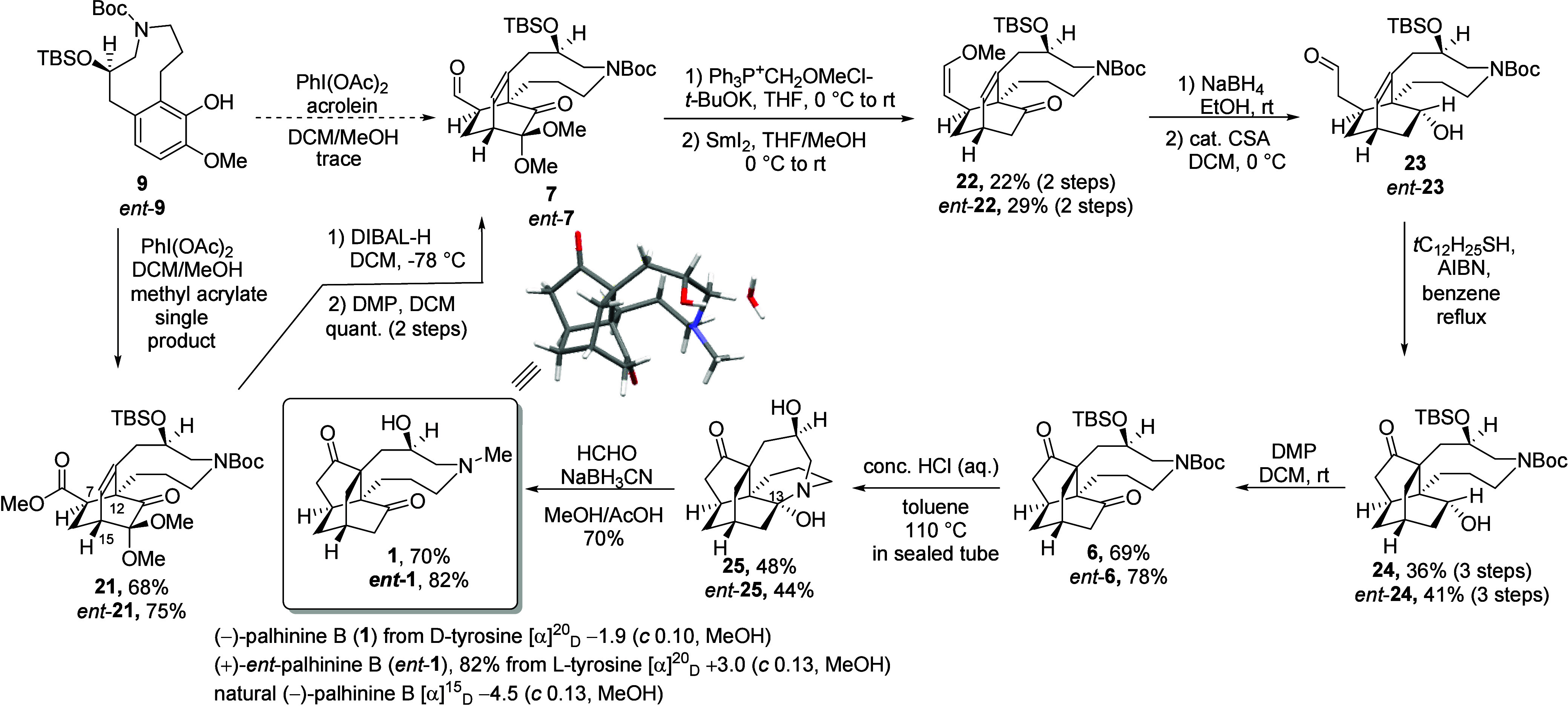
Asymmetric Total Synthesis of (−)-Palhinine B (**1**), and (+)-*ent*-Palhinine B (*ent*-**1**)

Construction of the final five-membered ring of the isotwistane skeleton was achieved through a thiol-mediated acyl radical cyclization^9^ from aldehyde precursor **23**, as shown in Scheme 3. Initial efforts to access aldehyde **23** as a radical precursor involved reductive demethoxylation of the geminal dimethoxy groups using samarium­(II) iodide, followed by reduction and homologation. However, this approach failed to produce the desired product from Diels–Alder adduct **21**. Therefore, an alternative route was pursued. Reduction and oxidation of methyl ester **21** provided aldehyde **7**. Wittig olefination gave an unstable mixture of *cis*- and *trans*-enol ether, which was directly subjected to reductive demethoxylation. During this process, the *trans*-enol ether was decomposed, and only *cis*-enol ether **22** was obtained in 22% yield over two steps. To prevent undesired rearrangement,^9b,9c^ the C13 ketone was reduced. Hydrolysis of enol ether **22** to obtain aldehyde **23** was initially attempted using PTSA based on previous reports,^5^ but this condition also removed the TBS protecting group. To avoid this side reaction, acids such as CSA and aq. HCl were screened, and CSA was ultimately found to provide the desired acyl radical precursor **23**. Due to its instability, aldehyde **23** was directly subjected to thiol-mediated acyl radical cyclization, forming the five-membered ring and delivering the palhinine tetracyclic framework **24** in 50% yield. Notably, C2-OH azonane intermediates **7**, **22**, and **23** were found to exhibit lower stability compared to their corresponding C3-OH analogues.^5^

The key tetracyclic intermediate **24** was initially oxidized to ketone **25** using Dess-Martin periodinane (DMP). Treatment with conc. aqueous HCl (aq.) in toluene enabled simultaneous removal of both TBS and Boc groups in a one-pot process. This was followed by spontaneous intramolecular aza-ketalization at C13 to give hemiaminal intermediate **25**. Finally, reductive methylation using formaldehyde and sodium cyanoborohydride furnished synthetic (−)-palhinine B (**1**) from d-tyrosine, and (+)-palhinine B from l-tyrosine. Comparison of the optical rotation with that of natural (−)-palhinine B (**1**) established its absolute configuration as 2*R*, 4*R*, 7*S*, 12*S*, 15*R*.^1b,11^

After completing the synthesis of palhinine B (**2**), we turned our attention to the inversion of the C2 hydroxy group to accomplish the total synthesis of palhinine C (**2**). As predicted by DFT analysis, reduction of the C2-ketone is kinetically disfavored. Therefore, we initially explored the possibility of C2-hydroxy inversion via S_N_2-type reaction using a nine-membered azonane ring intermediate, as shown in Scheme 4a. We first attempted a Mitsunobu reaction to directly invert the C2 hydroxy group from palhinine B (**1**), but only decomposition was observed. Considering that the tertiary amine might interfere with the Mitsunobu reaction, we removed the TBS group from silyl ether **6** to afford alcohol **26** bearing a Boc-protected amine. Attempts to obtain the inverted product **27** under Mitsunobu conditions were unsuccessful, resulting only in recovery of the starting material, likely due to the steric hindrance of the bulky Boc group. Subsequently, smaller leaving groups such as mesylate and triflate were introduced. However, these reactions also led to recovery of the starting material **26**, as shown in Scheme 4a. Since S_N_2 direct strategies failed to achieve the inversion of C2- hydroxy group, alternative approaches were investigated. We then considered caged hemiaminal intermediate **25**. Oxidation of **25** afforded the C2-ketone **29** as a reduction substrate.

**Scheme 4:**
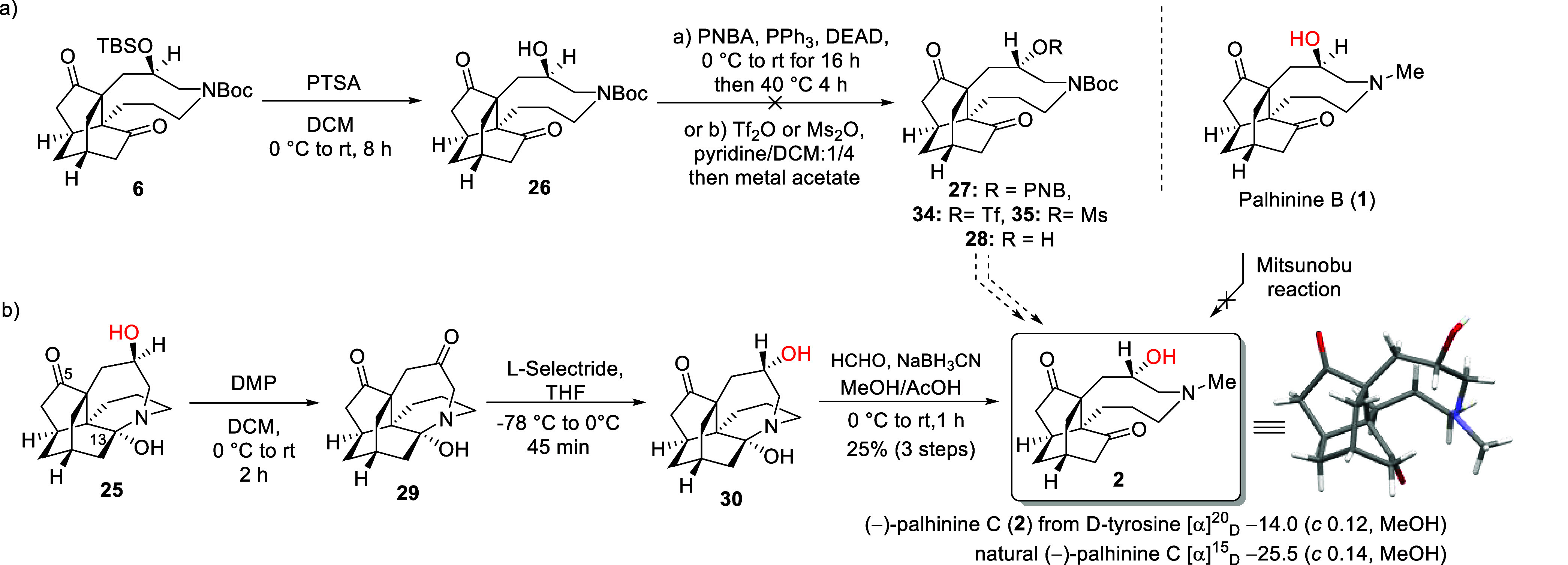
a) Synthetic Attempts for Inversion of C2-Hydroxy. b) Total Synthesis of (−)-Palhinine C (**2**)

A bulky reducing agent, L-Selectride, was employed for the diastereoselective reduction of the C2 carbonyl. Chemoselectivity was not problematic, as the C5 carbonyl is known to be tolerated under L-Selectride reduction,^4^ and the C13 carbonyl is protected through spontaneous intramolecular aza-ketalization. Reduction of ketone **29** with L-Selectride successfully delivered the desired C2-inversion product **30**. Finally, reductive methylation furnished the total synthesis of (−)-palhinine C (**2**) while the carbonyl reduction proceeded with complete diastereoselectivity. The structure and absolute configuration of (−)-palhinine C (**2**) were unambiguously confirmed as 2*S*, 4*R*, 7*S*, 12*S*, 15*R* by comparing the optical rotations of the natural product (−)-palhinine C (**2**) and X-ray crystallography, as shown in Scheme 4b.^1b,12^

In summary, we have accomplished the first asymmetric total synthesis of C2-OH *Lycopodium* alkaloids, including (−)-palhinine B (**1**), (−)-palhinine C (**2**), and (+)-*ent*-palhinine B (*ent* **-1**). Our strategy features several key elements: (a) Installation of the chiral C2-OH group on the azonane ring was achieved using chiral tyrosine, enabling rapid introduction of both the stereocenter and the nitrogen atom required for ring construction. (b) The isotwistane skeleton with the desired absolute configuration (C7, C12, C13, and C15) was constructed through a highly diastereoselective Diels–Alder reaction of MOB intermediate **8** with methyl acrylate, followed by a thiol-mediated acyl radical cyclization, furnishing tetracyclic intermediate **24**. The absolute configuration of the framework was ultimately governed by the chirality of the C2-OH group. (c) DFT-guided analysis enabled evaluation of late-stage C2-OH inversion strategies. The successful inversion was achieved through chemo and diastereoselective reduction of the C2 carbonyl in hemiaminal intermediate **29** using L-Selectride. (d) Notably, we observed that C2-OH azonane intermediates are less stable than their C3-OH counterparts, which may contribute to the synthetic challenges associated with C2-OH *Lycopodium* alkaloids.

## Supplementary Material



## Data Availability

The data underlying this study are available in the published article and its Supporting Information.

## Notes

General procedures, experimental procedures, copies of ^1^H & ^13^C NMR spectra, X-ray crystallography data for (−)-palhinine B (**1**) and (−)-palhinine C (**2**) (PDF)

## Abbreviations

AIBN: azobisisobutyronitrile
Boc: *tert*-butyloxycarbonyl
CSA: camphorsulfonic acid
DCM: dichloromethane
DEAD: diethyl azodicarboxylate
DIBAL-H: diisobutylaluminum hydride
DMP: Dess–Martin periodinane
2,2-DMP: 2,2-dimethoxypropane
*m*CPBA: *meta*-chloroperoxybenzoic acid
Ms (mesylate): methanesulfonyl
MOB: masked *ortho*-benzoquinone
PPTS: pyridinium *para*-toluenesulfonate
PNBA: *para*-nitrobenzoic acid
PTSA: *para*-toluenesulfonic acid
TBS: *tert*-butyldimethylsilyl
Tf (triflate): trifluoromethanesulfonyl
THF: tetrahydrofuran
