# Supplementary material for: Asymmetric Total Synthesis of C2-OH Lycopodium Alkaloids (−)-Palhinine B, (−)-Palhinine C, and (+)-Palhinine B Enabled by Stereocontrolled Diels–Alder Strategy
Source: Org Lett. 2026 Jun 15;28(25):7965–70. doi: 10.1021/acs.orglett.6c01704 (PMC13316983; doi:10.1021/acs.orglett.6c01704)
Supplement: Supplementary file 1 [file ol6c01704_si_003.pdf]

Supporting Information

Asymmetric Total Synthesis of C2-OH Lycopodium Alkaloids (−)-Palhinine B, (−)-Palhinine C, and (+)-Palhinine B Enabled by Stereocontrolled Diels–Alder Strategy

Amit Pantawane,[a,b,] Chih-Ming Chen,[a,] Tung-Chun Kuo,[c] Julakanti Satyanarayana Reddy,[a] Mu-Jeng Cheng,[c] Shun-Yuan Luo,[b,*] Hsing-Pang Hsieh[a,d,*]

a. Institute of Biotechnology and Pharmaceutical Research, National Health Research Institutes, Miaoli County 350, Taiwan, ROC.

b. Department of Chemistry, National Chung Hsing University, Taichung 402, Taiwan, ROC.

c. Department of Chemistry, National Cheng Kung University, Tainan 701, Taiwan, ROC.

d. Department of Chemistry, National Tsing Hua University, Hsinchu 300, Taiwan, ROC.

*Email: *hphsieh@nhri.edu.tw;*

**syluo@dragon.nchu.edu.tw*

Table of Contents

1. General Procedures..S2
2. Synthetic Procedure and Characterization of Compounds S3
3. Natural Product Spectral Comparisons..S20
4. Computational Details..S26
5. References..S37
6. X-Ray Crystal Data..S38
7. ^{1}H- and ^{13}C-NMR Spectra..S42
8. HPLC Spectra S87

1. General Procedures

Unless stated otherwise, reagents were obtained from commercial sources and used without further purification. All moisture- or oxygen-sensitive reactions were performed under positive pressure of anhydrous argon or nitrogen in anhydrous solvents, which were dried prior to use following standard procedures. Thin-layer chromatography (TLC) was performed using Merck 5554 DC-Alufolien Kieselgel 60 F254. Flash column chromatography was performed using 230–400 mesh silica gel from Merck Art.9385 Kieselgel 60H. Except as otherwise indicated, yields were calculated after flash column chromatography. Reactions requiring elevated temperatures were performed using an oil bath under the specified conditions unless otherwise noted. ^{1}H-NMR and ^{13}C-NMR were measured by using Bruker AVANCE NEO-400 MHz and Bruker AVANCE NEO-600 MHz spectrometers. Chemical shifts are reported as δ values in ppm, and calibrated using residual undeuterated solvent ($CDCl_3$ (7.26 ppm) or CD_3OD (3.31 ppm)) as internal reference for ^{1}H NMR and the deuterated solvent ($CDCl_3$ (77.00 ppm) or CD_3OD (49.00 ppm)) as internal standard for ^{13}C NMR. Coupling constants are reported in Hz; multiplicities are indicated as follows: s = singlet, d = doublet, t = triplet, q = quartet, quint = quintet, m = multiplet (denotes complex pattern), dd = doublet of doublets, dt = doublet of triplets, td = triplet of doublets, and br = broad signal. Infrared (IR) spectra were recorded on a FT/IR-4X1typeA and are reported in wavenumbers (cm-1). High-resolution mass spectra (HRMS) were recorded using a VARIAN 901-MS(TOF). Melting points were determined with a Krüss Optronic KSP1N melting point meter. Optical rotations were recorded using an Anton Paar MCP 5100 polarimeter instrument with 1 dm pathlength cell. X-ray diffraction analysis was measured on a Bruker D8 Dual Single Crystal X-ray Diffractometer (D8 Venture IuS 3.0 Dual source) with monochromatic MoKα radiation. High-performance liquid chromatography (HPLC) was performed on JASCO instruments (UV-2075 plus/EC-7000/ADC/PU-2089 plus) using Daicel Chiralpak IA (250×4.6 mm) analytical columns with an HPLC-grade solvent mixture, as indicated in each experiment. Specific rotations ($[\alpha]^{T}_{D}$) were measured on a JASCO Co. DIP-1000 digital polarimeter at the stated temperature with a sodium lamp (D line, λ = 589 nm). Measurements were performed in a 50 mm length cell with concentrations (c given in g/100 mL) reported in the corresponding solvent.

2. Synthetic Procedure and Characterization of Compounds

For Compound 11 and *ent*-11[1]

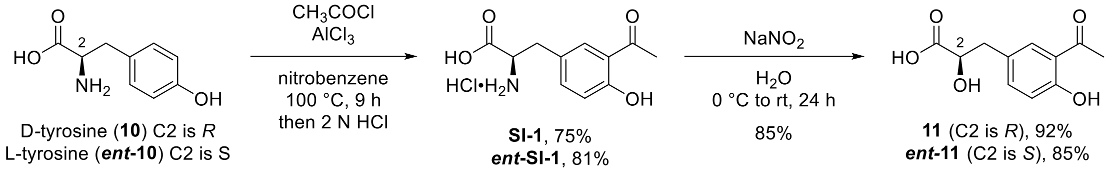

Anhydrous aluminium chloride (73.54 g, 551.93 mmol) was added slowly to a solution of tyrosine (***ent*-10**, 25.00 g, 137.98 mmol) in dry nitrobenzene (400 mL) at rt. This slightly exothermic reaction was stirred at rt for 10 minutes. Then acetyl chloride (11.77 mL, 66.23 mmol) was added dropwise over 1 minute, during which a color change was observed from red to yellow. The reaction mixture was heated at 100 °C with stirring for 9 hours and turned into a dark green thick gel after cooling down to room temperature. The thick gel was dissolved in cold 2.0 N aqueous solution of HCl (2.5 L). The nitrobenzene layer was separated, and the aqueous phase was washed with ethyl acetate (2 × 100 mL). The aqueous layer was concentrated to ca. 500 mL in volume and then kept at 4 °C for 14 hours. The desired product ***ent*-SI-1** precipitated as a pearl white solid and was collected via filtration and recrystallized with 6.0 N aqueous solution of HCl to get hydrochloride salt (29.15 g, 112.25 mmol, 81%).

For **SI-1**: $[\alpha]^{20}_{D} = +2.0$ (*c* 1.0, H_2O) (lit.[1a] $[\alpha]^{20}_{D} = +2.0$ (*c* 0.5, MeOH); for ***ent*-SI-1**: $[\alpha]^{20}_{D} = -2.6$ (*c* 1.0, H_2O) (lit.[2] $[\alpha]^{25}_{D} = -2.3$ (*c* 1.0, MeOH)).
m.p.: 224.4–225.8 °C.
^{1}H NMR (400 MHz, D_2O): δ 7.75 (d, J = 2.2 Hz, 1H), 7.42 (dd, J = 8.6, 2.2 Hz, 1H), 6.92 (d, J = 8.6 Hz, 1H), 4.23 (dd, J = 7.2, 5.6 Hz, 1H), 3.25 (dd, J = 14.6, 5.6 Hz, 1H), 3.16 (dd, J = 14.6, 7.2 Hz, 1H), 2.60 (s, 3H).[1]
IR (NaCl): ν 3420, 1753 cm^{-1}.
HRMS (ESI) m/z: $[M-H]^-$ Calcd for $C_{11}H_{13}ClNO_4$ 258.0533; Found 258.0534.

The salt compound ***ent*-SI-1** (40.00 g, 154.03 mmol) was dissolved in water (1.00 L) and cooled in an ice-water bath. The solution of $NaNO_2$ (18.07 g, 261.86 mmol) in water (0.50 L) was added dropwise and slowly stirred. After the solution was stirred at room temperature for 20 hours, ammonium sulfate (8.95 g, 67.77 mmol) was added and stirred for a further 1 hour. The crude product was extracted in ethyl acetate (adjust pH = 2 by addition of 2.0 N aqueous solution of HCl), and the separated organic phase was dried on $MgSO_4$. Excess solvent was removed by rotary evaporator and purified with silica gel column chromatography (EtOAc/Hex: 2:8–7:2) to afford α-hyddroxy acid ***ent*-11** (29.51 g, 131.62 mmol, 85%) as a yellow oil.

For **11**: $[\alpha]^{20}_{D}$ = +10.0 (*c* 0.5, MeOH) (lit.[1a] $[\alpha]^{20}_{D}$ = +14.8 (*c* 0.5, MeOH); for ***ent*-11**: $[\alpha]^{25}_{D}$ = −12.0 (*c* 1.0, H_2O) (lit.[1b] $[\alpha]^{25}_{D}$ = −12.0 (*c* 0.64, MeOH).

^{1}H NMR (600 MHz, $CDCl_3$): *δ* 12.18 (s, 1H, -OH), 7.63 (d, *J* =2.1 Hz, 1H), 7.37 (dd, *J* =8.7, 2.1 Hz, 1H), 6.92 (d, *J* =8.7 Hz, 1H), 4.49 (dd, *J* = 6.6, 4.2 Hz, 1H), 3.15 (dd, *J* = 14.4, 4.2 Hz, 1H), 2.97 (dd, *J* = 14.4, 6.6 Hz, 1H), 2.62 (s, 3H).[1b]

^{13}C NMR (150 MHz, $CDCl_3$): *δ* 204.6, 177.3, 161.4, 137.7, 131.7, 126.4, 119.5, 118.5, 70.8, 39.0, 26.7.[1b]

IR (KBr): *ν* 2925, 2854, 1727, 1641, 1466, 1368, 1299 cm^{-1}.

HRMS (ESI) m/z: $[M-H]^-$ Calcd for $C_{11}H_{11}O_5$ 223.0606; Found 223.0603.

For Compound 12 and *ent*-12

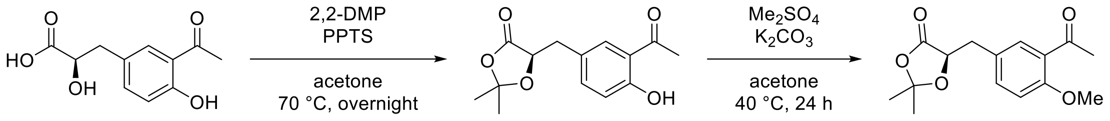

11
***ent*-11**

SI-2, 60%
***ent*-SI-2**, 82%

12, 98%
***ent*-12**, 99%

A solution of acid ***ent*-11** (33.00 g, 147.18 mmol), 2,2-dimethoxypropane (90.17 mL, 735.59 mmol) and pyridinium *p*-toluenesulphonate (18.49 g, 73.59 mmol) in acetone (735 mL) was stirred at 70 °C for overnight. The mixture was cooled to ambient temperature and excess solvent was removed by rotary evaporator. The crude oil was purified by silica gel column chromatography (EA/Hex: 15:85) to afford the desired product ***ent*-SI-2** (31.86 g,120.56 mmol, 82 %) as a yellow oil.

For **SI-2**: $[\alpha]^{20}_{D}$ = +29.9 (*c* 1.0, DCM); for ***ent*-SI-2**: $[\alpha]^{20}_{D}$ = −17.0 (*c* 1.0, DCM).

^{1}H NMR (400 MHz, $CDCl_3$): *δ* 12.18 (s, 1H), 7.60 (d, *J* =2.4 Hz, 1H), 7.37 (dd, *J* = 8.6, 2.4 Hz, 1H), 6.93 (d, *J* = 8.6 Hz, 1H), 4.65 (dd, *J* = 5.3, 4.4 Hz, 1H), 3.14 (dd, *J* = 14.6, 4.4 Hz, 1H), 3.04 (dd, *J* = 14.6, 5.3 Hz, 1H), 2.63 (s, 3H), 1.51 (s, 3H), 1.36 (s, 3H).

^{13}C NMR (150 MHz, $CDCl_3$): *δ* 204.5, 172.2, 161.5, 138.4, 132.0, 125.8, 119.4, 118.3, 110.9, 74.9, 36.3, 26.9, 26.7, 26.1.

IR (KBr): *ν* 2993, 2928, 1791, 1643, 1620, 1487, 1377, 1252 cm^{-1}.

HRMS (ESI) m/z: $[M+H]^+$ Calcd for $C_{14}H_{16}NaO_5$ 287.0895; Found 287.0893.

To a solution of phenol ***ent*-SI-2** (29.46 g, 111.46 mmol) in acetone (560 ml), anhydrous potassium carbonate (30.81 g, 222.93 mmol) was added slowly at 0 °C, and stirred for 5 min with a magnetic stirrer. Dimethyl sulfate (15.86 mL, 167.19 mmol) was added dropwise over 10 min, and the resultant solution was heated to 40 °C for 24 h. After the completion of phenol, excess K_2CO_3 was filtered out using a pad of celite. The filtrate was concentrated with a rotary evaporator, and purified by column

chromatography (EA/Hex: 15:85–25:75) to afford pure product ***ent*-12** (30.67 g, 110.20 mmol, 99%) as a yellowish oil.

For **12**: $[\alpha]^{20}_{D}$ = +18.9 (*c* 1.0, DCM): for ***ent*-12**: $[\alpha]^{20}_{D}$ = −23.0 (*c* 1.0, DCM).

^{1}H NMR (400 MHz, $CDCl_3$): *δ* 7.64 (d, *J* = 2.4 Hz, 1H), 7.35 (dd, *J* = 8.4, 2.4 Hz, 1H), 6.91 (d, *J* = 8.4 Hz, 1H), 4.62 (dd, *J* = 6.6, 4.2 Hz, 1H), 3.90 (s, 3H), 3.16 (dd, *J* = 14.6, 4.2 Hz, 1H), 2.99 (dd, *J* = 14.6, 6.6 Hz, 1H), 2.60 (s, 3H), 1.50 (s, 3H), 1.42 (s, 3H).

^{13}C NMR (100 MHz, $CDCl_3$): *δ* 199.6, 172.3, 158.1, 134.9, 131.7, 128.0, 127.8, 111.7, 110.8, 74.9, 55.6, 36.5, 31.8, 27.0, 26.1.

IR (KBr): *ν* 2994, 2942, 2842, 1792, 1674, 1609, 1498, 1293, 1255, 1119 cm^{-1}.

HRMS (ESI) m/z: $[M+H]^+$ Calcd for $C_{15}H_{19}O_5$ 279.1232; Found 279.1234.

For Compound 13 and *ent*-13

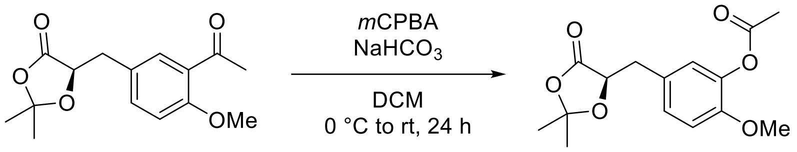

12
***ent*-12**

13, 83%
***ent*-13**, 91%

Ketone ***ent*-12** (30.67 g, 110.18 mmol) was dissolved in dry dichloromethane (1.10 L), $NaHCO_3$ (27.77 g, 330.56 mmol), and *meta*-chloroperoxybenzoic acid (76.06 g, 330.56 mmol) were added at 0 °C and stirred with a magnetic stirrer for 24h at rt. After the completion, it was diluted with excess sat. aqueous solution of $NaHCO_3$ and extract with DCM (3 × 200 mL). The combined organic extracts were dried over $MgSO_4$, concentrated in vacuo to afford the desired product ***ent*-13** as a crude greenish oil (29.49 g, 100.20 mmol, 91%) and used for the next step without further purification.

^{1}H NMR (400 MHz, $CDCl_3$): *δ* 7.08 (dd, *J* = 8.2, 2.2 Hz, 1H), 6.95 (d, *J* = 2.2 Hz, 1H), 6.90 (d, *J* = 8.2 Hz, 1H), 4.61 (dd, *J* = 6.2, 4.2 Hz, 1H), 3.81 (s, 3H), 3.11 (dd, *J* = 14.6, 4.2 Hz, 1H), 3.00 (dd, *J* = 14.6, 6.2 Hz, 1H), 2.30 (s, 3H), 1.50 (s, 3H), 1.38 (s, 3H).

^{13}C NMR (150 MHz, $CDCl_3$): *δ* 172.4, 169.0, 150.2, 139.4, 128.2, 124.4, 111.2, 110.9, 74.9, 55.9, 36.5, 26.9, 26.1, 20.6.

For Compound 14 and *ent*-14

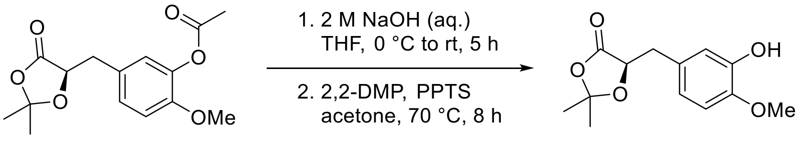

13
***ent*-13**

14, 59% (2 steps)
***ent*-14**, 60% (2 steps)

A 2 M aqueous solution of NaOH (276 mL) was added to the solution of phenyl

acetate ***ent*-13** (30.67 g, 110.20 mmol) in THF (500 mL) at 0 °C. After the mixture was stirred for 5 hours, 2 N aqueous solution of HCl was added until the pH was close to 2. The resulting aqueous solution was extracted with ethyl acetate (3 × 100 mL). The combined organic phase was washed with brine and then dried over anhydrous $MgSO_4$. After the removal of the solvent under reduced pressure, the crude product was submitted for the next step without further purification. A solution of the crude product (23.38 g, 110.15 mmol), 2,2-dimethoxypropane (67.48 mL, 550.75 mmol), and pyridinium *p*-toluenesulfonate (2.77 g, 11.02 mmol) in acetone (740 mL) was stirred at 70 °C for 8 h. The mixture was cooled to ambient temperature and excess solvent was removed by rotary evaporator. The crude oil was purified by silica gel column chromatography (EtOAc/Hexane: 15:85), which gave the desired product ***ent*-14** (16.74 g, 66.37 mmol, 60%) as a yellow oil.

For **14**: $[\alpha]^{20}_{D}$ = +37.9 (*c* 1.0, DCM); for ***ent*-14**: $[\alpha]^{20}_{D}$ = −32.0 (*c* 1.0, DCM).
^{1}H NMR (400 MHz, $CDCl_3$): δ 6.85 (d, *J* = 2.2 Hz, 1H), 6.79 (d, *J* = 8.2 Hz, 1H), 6.73 (dd, *J* = 8.2, 2.2 Hz, 1H), 5.59 (s, 1H), 4.60 (dd, *J* = 7.0, 3.8 Hz, 1H), 3.87 (s, 3H), 3.11 (dd, *J* = 14.6, 3.8 Hz, 1H), 2.93 (dd, *J* = 14.6, 7.0 Hz, 1H), 1.50 (s, 3H), 1.43 (s, 3H).
^{13}C NMR (100 MHz, $CDCl_3$): δ 172.5, 145.7, 145.4, 129.1, 121.2, 116.0, 110.8, 110.5, 75.1, 55.9, 37.2, 27.1, 26.1.
IR (KBr): ν 2960, 2926, 2854, 1792, 1594, 1459, 1261, 1093 cm^{-1}.
HRMS (ESI) m/z: $[M+Na]^+$ Calcd for $C_{13}H_{16}NaO_5$ 275.0895; Found 275.0898.

For Compound 15 and *ent*-15

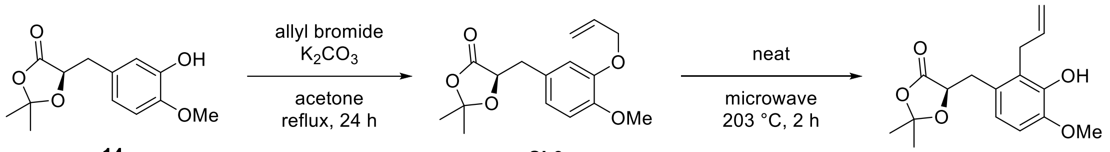

14
***ent*-14**

SI-3
***ent*-SI-3**

15, 54 % (2 steps)
***ent*-15**, 60 % (2 steps)

To a solution of phenol ***ent*-14** (16.74 g, 66.36 mmol) in acetone (442 mL) was added K_2CO_3 (18.34 g, 132.73 mmol) and allyl bromide (8.62 mL, 99.54 mmol) at 0 °C. The reaction mixture was stirred at reflux temperature for 20 h. The mixture was cooled to ambient temperature, and excess solvent was removed by rotary evaporator. The crude ***ent*-SI-3** yellowish oil was submitted to the next step without further purification.

^{1}H NMR (400 MHz, $CDCl_3$): δ 6.82–6.77 (m, 3H), 6.08 (ddt, *J* = 17.2, 10.4, 5.4 Hz, 1H), 5.41 (ddt, *J* = 17.2, 1.4, 1.4 Hz, 1H), 5.28 (ddt, *J* = 10.4, 1.4, 1.4 Hz, 1H), 4.62 (dd, *J* = 6.0, 4.2 Hz, 1H), 4.60 (ddd, *J* = 5.4, 1.4, 1.4 Hz, 2H), 3.85 (s, 3H), 3.10 (dd, *J* =

14.6, 4.2 Hz, 1H), 2.99 (dd, J = 14.6, 6.0 Hz, 1H), 1.50 (s, 3H), 1.35 (s, 3H).
^{13}C NMR (100 MHz, $CDCl_3$): δ 172.5, 148.5, 147.6, 133.2, 128.0, 122.4, 118.1, 115.2, 111.4, 110.8, 75.2, 69.8, 55.9, 37.1, 27.0, 26.2.
HRMS (ESI) m/z: $[M+Na]^+$ Calcd for $C_{16}H_{20}NaO_5$ 315.1208; Found 315.1208.

The crude allyl ether ***ent*-SI-3** (18.30 g, 62.60 mmol) was heated at 203 °C and stirred for 2 h in a microwave oven. After cooling to room temperature, the crude product was purified with silica gel column chromatography (EtOAc/Hexane: 10:90) to afford the desired product ***ent*-15** (11.64 g, 39.83 mmol, 60% yield over 2 steps) as a white solid.

For **15**: $[\alpha]^{20}_D$ = +37.9 (c 1.0, DCM); for ***ent*-15**: $[\alpha]^{20}_D$ = −29.0 (c 1.0, DCM).
m.p.: 68.4–69.4.
^{1}H NMR (400 MHz, $CDCl_3$): δ 6.77 (d, J = 8.2 Hz, 1H), 6.72 (d, J = 8.2 Hz, 1H), 5.97 (ddt, J = 17.0, 10.2, 5.8 Hz, 1H), 5.72 (s, 1H), 5.00 (ddt, J = 10.2, 1.8, 1.8 Hz, 1H), 4.93 (ddt, J = 17.0, 2.0, 1.8 Hz, 1H), 4.61 (dd, J = 7.8, 3.8 Hz, 1H), 3.87 (s, 3H), 3.59–3.44 (m, 2H), 3.24 (dd, J = 14.8, 3.8 Hz, 1H), 2.91 (dd, J = 14.8, 7.8 Hz, 1H), 1.49 (s, 3H), 1.46 (s, 3H).
^{13}C NMR (100 MHz, $CDCl_3$): δ 172.8, 145.4, 143.7, 136.1, 128.1, 124.6, 121.2, 114.8, 110.8, 108.4, 75.1, 55.9, 34.2, 30.2, 27.1, 26.0.
IR (NaCl): ν 3495, 3077, 2963, 2933, 1790, 1637, 1619, 1588, 1494, 1462, 1275 cm^{-1}.
HRMS (ESI) m/z: $[M+Na]^+$ Calcd for $C_{16}H_{20}NaO_5$ 315.1208; Found 315.1211.

For Compound 16 and *ent*-16

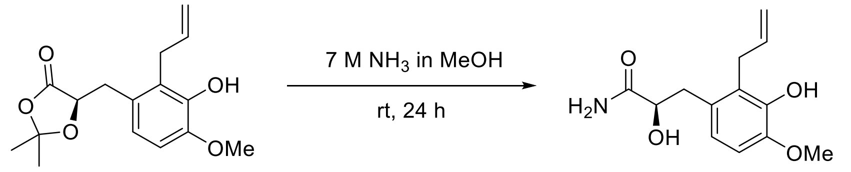

15
***ent*-15**

16, 83%
***ent*-16**, 83%

Phenol ***ent*-15** (11.64 g, 39.83 mmol) was dissolved in a solution of NH_3 (7.0 N in anhydrous MeOH, 56.90 mL) at 0 °C, and the mixture was stirred under an argon atmosphere for 18 h at room temperature. The mixture was then concentrated in vacuo, and the residue was purified using silica gel column chromatography (EtOAc/Hexane: 90:10–100:0), yielding amide ***ent*-16** (8.35 g, 33.24 mmol, 83%) as a colorless gum.

For **16**: $[\alpha]^{20}_D$ = +26.9 (c 1.0, DCM); for ***ent*-16**: $[\alpha]^{20}_D$ = −10.0 (c 1.0, DCM).
^{1}H NMR (400 MHz, $CDCl_3$): δ 6.75 (s, 2H), 6.38 (br, 1H), 6.00 (ddt, J =17.2, 10.2, 5.8 Hz, 1H), 5.79 (s, 1H), 5.54 (br, 1H), 5.03 (ddt, J = 10.2, 1.8, 1.8 Hz, 1H), 4.96 (ddt, J = 17.2, 1.8, 1.8 Hz, 1H), 4.26 (dd, J = 9.4, 4.2 Hz, 1H), 3.88 (s, 3H), 3.60–3.43 (m, 2H),

3.28 (dd, J = 14.4, 4.2 Hz, 1H), 2.78 (dd, J = 14.4, 9.4 Hz, 1H), 2.47 (br, 1H).
^{13}C NMR (150 MHz, $CDCl_3$): δ 175.4, 145.5, 144.2, 136.4, 128.4, 124.7, 121.2, 115.1, 108.7, 72.4, 56.0, 37.5, 30.2.
IR (NaCl): ν 3465, 3353, 2962, 2939, 2840, 1668, 1585, 1493, 1277, 1087 cm^{-1}.
HRMS (ESI) m/z: $[M+Na]^+$ Calcd for $C_{13}H_{17}NNaO_4$ 274.1055; Found 274.1057.

For Compound 17

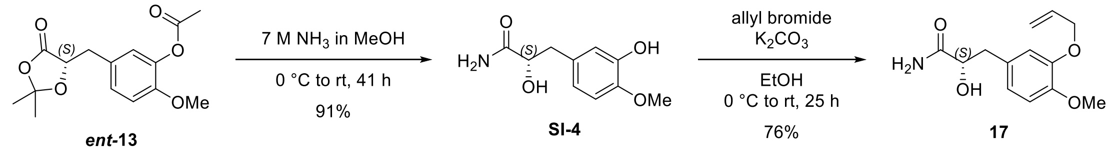

ent-13 SI-4 17

Phenyl acetate ***ent*-13** (3.82 g, 12.96 mmol) was dissolved in a solution of 7.0 M NH_3 in anhydrous MeOH (27.78 mL) at 0 °C, and the mixture was stirred under an argon atmosphere for 41 h at rt. The mixture was then concentrated in vacuo, and the residue was purified using column chromatography (MeOH/DCM: 1:9), yielding amide **SI-4** (2.49 g, 11.77 mmol, 91%) as a light orange solid.

$[\alpha]^{20}{}_D = -30.0$ (c 1.0, MeOH).
m.p.: 92.6–94.5.
^{1}H NMR (400 MHz, CD_3OD): δ 6.82 (d, J = 8.2 Hz, 1H), 6.76 (d, J = 2.0 Hz, 1H), 6.70 (dd, J = 8.2, 2.0 Hz, 1H), 4.16 (dd, J = 8.2, 3.8 Hz, 1H), 3.81 (s, 3H), 2.98 (dd, J = 13.8, 3.8 Hz, 1H), 2.70 (dd, J = 13.8, 8.2 Hz, 1H).
^{13}C NMR (100 MHz, CD_3OD): δ 179.8, 147.7, 147.2, 132.1, 121.9, 117.7, 112.6, 74.0, 56.5, 41.2.
IR (KBr): ν 3364, 2951, 2934, 2915, 2842, 1682, 1591, 1514, 1444, 1277, 1238, 1081, 966 cm^{-1}.
HRMS (ESI) m/z: $[M+Na]^+$ Calcd for $C_{10}H_{13}NNaO_4$ 234.0737, Found 234.0743.

To a solution of phenol **SI-4** (2.37 g, 11.22 mmol) in ethanol (74.5 mL) was added K_2CO_3 (1.86 g, 13.47 mmol) and allyl bromide (1.17 mL, 13.47 mmol) at 0 °C. The reaction mixture was stirred at room temperature for 25 h. After completion, it was filtered, concentrated, and purified with silica gel column chromatography (MeOH/DCM: 4:96) to afford the desired product **17** (2.14 g, 8.52 mmol, 76%) as a white solid.

$[\alpha]^{28}{}_D = -35.6$ (c 1.0, MeOH).
m.p.: 150.3–155.9.
^{1}H NMR (400 MHz, CD_3OD): δ 6.90 (d, J = 1.8 Hz, 1H), 6.87 (d, J = 8.2 Hz, 1H), 6.82

(dd, J = 8.2, 1.8 Hz, 1H), 6.08 (ddt, J = 17.4, 10.6, 5.4 Hz, 1H), 5.40 (ddt, J = 17.4, 1.4, 1.4 Hz, 1H), 5.24 (ddt, J = 10.6, 1.4, 1.4 Hz, 1H), 4.56 (ddd, J = 5.4, 1.4, 1.4 Hz, 2H), 4.17 (dd, J = 8.0, 3.8 Hz, 1H), 3.80 (s, 3H), 3.01 (dd, J = 13.8, 3.8 Hz, 1H), 2.76 (dd, J = 13.8, 8.0 Hz, 1H).

^{13}C NMR (100 MHz, CD_3OD): δ 179.7, 149.7, 149.1, 135.1, 132.0, 123.6, 117.7, 116.9, 113.3, 74.0, 71.1, 56.6, 41.2.

IR (KBr): ν 3441, 3345, 2934, 2834, 1667, 1652, 1591, 1515, 1423, 1259, 1139, 1022 cm^{-1}.

HRMS (ESI) m/z: [M+Na]$^+$ Calcd for $C_{13}H_{17}NNaO_4$ 274.1050; found 274.1055.

For Compound *ent*-16 from Compound 17

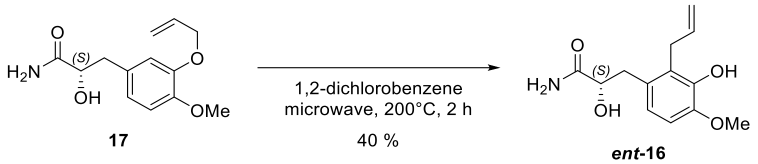

Allyl ether **17** (1.02 g, 4.05 mmol) in 1,2-dichlorobenzene (20.23 mL) was heated at 200 °C and stirred for 2 h in a microwave oven. After cooling to room temperature, the crude product was concentrated and purified with column chromatography (EtOAc/Hexane: 90:10–100:0) to afford the desired product ***ent*-16** (0.41 g, 1.62 mmol, 40%) as a colorless gum.

For Compound 18 and *ent*-18

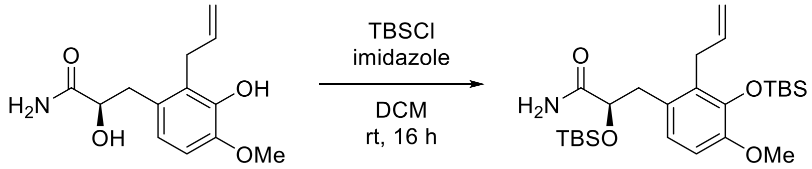

To a solution of diol ***ent*-16** (8.35 g, 33.24 mmol) and imidazole (13.57 g, 199.37 mmol, 6 equiv.) in dichloromethane (332 mL) was slowly added TBSCl (15.03 g, 99.73 mmol). After stirring at room temperature for 16 h, the suspension was extracted with water and dichloromethane (3× 100 mL). The combined organic layer was dried over $MgSO_4$, filtered, and concentrated *in vacuo*. The crude residue was purified by column chromatography with silica gel (EtOAc/Hex: 15:85) to afford silyl ether ***ent*-18** (14.10 g, 29.39 mmol, 88%) as a colourless gum.

For **18**: $[\alpha]^{20}_D$ = +35.9 (c 1.0, DCM); for ***ent*-18**: $[\alpha]^{20}_D$ = −33.0 (c 1.0, DCM).

^{1}H NMR (600 MHz, $CDCl_3$): δ 6.76 (d, J = 8.4 Hz, 1H), 6.67 (d, J = 8.4 Hz, 1H), 6.48 (br d, J = 3.9 Hz, 1H), 5.90 (ddt, J =17.1, 10.5, 5.7 Hz, 1H), 5.50 (br d, J = 3.9 Hz, 1H),

4.96 (ddt, J = 10.5, 1.8, 1.8 Hz, 1H), 4.92 (ddt, J = 17.1, 1.8, 1.8 Hz, 1H), 4.18 (dd, J = 9.9, 2.7 Hz, 1H), 3.76 (s, 3H), 3.59–3.50 (m, 2H), 3.15 (dd, J = 14.1, 2.7 Hz, 1H), 2.71 (dd, J = 14.1, 9.9 Hz, 1H), 0.98 (s, 9H), 0.83 (s, 9H), 0.17 (s, 3H), 0.15 (s, 3H), −0.18 (s, 3H), −0.39 (s, 3H).

^{13}C NMR (150 MHz, $CDCl_3$): δ 177.1, 148.6, 142.9, 136.6, 130.1, 129.3, 123.6, 114.8, 108.9, 75.3, 54.6, 38.5, 30.8, 26.2, 25.7, 18.9, 17.9, −3.8, −3.9, −5.6, −5.8.

IR (NaCl): ν 2956, 2956, 2931, 2858, 1689, 1584, 1283, 1094 cm^{-1}.

HRMS (ESI) m/z: $[M+Na]^+$ Calcd for $C_{25}H_{45}NNaO_4Si_2$ 502.2784; Found 502.2788.

For Compound 20 and *ent*-20

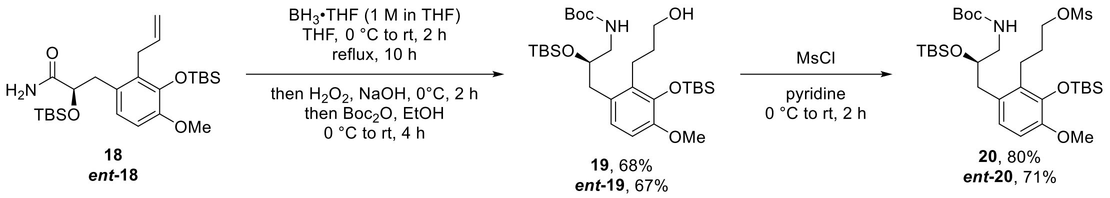

A dried Schlenk tube was charged with the amide ***ent*-18** (14.10 g, 29.39 mmol) in THF (300 mL), and 1.0 M BH_3•THF in THF (147.0 mL, 147.0 mmol) was added at 0 °C. The resulting mixture was stirred at rt for 2 h, and it was then refluxed for 12 h. After the reaction mixture was cooled to 0 °C, EtOH (44.1 mL), 2 M NaOH (aq., 44.1 mL), and H_2O_2 (44.1 mL) were added and stirred for 2 h at the same temperature. Finally, the solution of Boc_2O (9.62 g, 44.09 mmol) in ethanol (15.0 mL) was added dropwise at 0 °C and stirred at room temperature for 4 hours. After the completion, the reaction mixture was diluted with dichloromethane and washed with water, and sat. $NaHCO_3$ aqueous solution, organic layer was separated, dried with $MgSO_4$, filtered, and purified with column chromatography (EA/Hex: 5:95 to 15:85) to obtain the desired product ***ent*-19** (11.47 g, 19.63 mmol, 67%) as a colorless oil.

For **19**: $[\alpha]^{20}_{D}$ = +3.0 (c 1.0, DCM); for ***ent*-19**: $[\alpha]^{20}_{D}$ = −4.7 (c 1.0, DCM).

^{1}H NMR (400 MHz, $CDCl_3$): δ 6.71 (d, J = 8.4 Hz, 1H), 6.63 (d, J = 8.4 Hz, 1H), 4.84 (br, 1H), 3.90 (br, 1H), 3.74 (s, 3H), 3.60 (t, J = 6.4 Hz, 2H), 3.25–3.05 (m, 2H), 2.88–2.57 (m, 4H), 1.76 (tt, J = 6.4, 6.4 Hz, 2H), 1.45 (s, 9H), 0.99 (s, 9H), 0.84 (s, 9H), 0.18 (s, 3H), 0.17 (s, 3H), −0.07 (s, 3H), −0.30 (s, 3H).

^{13}C NMR (150 MHz, $CDCl_3$): δ 156.0, 147.1, 144.4, 129.2, 121.7, 121.6, 108.7, 79.2, 71.9, 66.1, 55.8, 46.2, 37.5, 31.9, 29.7, 29.4, 28.4, 25,8, 22.7, 22.2, 18.0, 14.1, −5.0, −5.0.

IR (NaCl): ν 2953, 2930, 2857, 1706, 1491, 1441, 1253, 1170 cm^{-1}.

HRMS (ESI) m/z: $[M+Na]^+$ Calcd for $C_{30}H_{57}NNaO_6Si_2$ 606.3622; Found 606.3623.

To a solution of alcohol **19** (10.4 g, 17.8 mmol) in pyridine (89 mL) was added MsCl (1.65 mL, 21.4 mmol) at 0 °C. The reaction mixture was warmed to room temperature and stirred for 2 h, then was diluted with dichloromethane (100 mL) and half brine (100 mL). The layers were separated, and the aqueous phase was extracted with CH2Cl2 (2 × 100 mL). The combined organic extracts were washed with brine (50 mL), dried over MgSO4, and concentrated in vacuo. The crude residue was purified by column chromatography with silica gel (EtOAc/Hex: 15:85 to 25:75) to afford mesylate **20** (9.37g, 14.15 mmol, 80% yield) as a colorless gum.

For **20**: $[\alpha]^{20}_{D}$ = +2.9 (*c* 1.0, DCM); for ***ent*-20**: $[\alpha]^{20}_{D}$ = −2.6 (*c* 1.0, DCM).
^{1}H NMR (400 MHz, $CDCl_3$): δ 6.71 (d, J = 8.2 Hz, 1H), 6.65 (d, J = 8.2 Hz, 1H), 4.81 (br, 1H), 4.23 (t, J = 6.6 Hz, 2H), 3.87 (br, 1H), 3.75 (s, 3H), 3.24–3.08 (m, 2H), 3.00 (s, 3H), 2.89–2.55 (m, 4H), 2.01–1.90 (m, 2H), 1.45 (s, 9H), 1.00 (s, 9H), 0.83 (s, 9H), 0.19 (s, 3H), 0.18 (s, 3H), −0.06 (s, 3H), −0.31 (s, 3H).
^{13}C NMR (100 MHz, $CDCl_3$): δ 156.0, 148.3, 143.0, 130.7, 129.4, 123.4, 109.2, 79.0, 72.7, 69.7, 54.6, 46.4, 38.1, 37.4, 29.2, 28.4, 26.2, 25.8, 23.1, 19.0, 18.0, −3.7, −3.7, −4.9, −5.0.
IR (NaCl): ν 3411, 2956, 2931, 2857, 1712, 1493, 1362, 1296, 1253, 1174, 1093 cm^{-1}.
HRMS (ESI) m/z: $[M+Na]^+$ Calcd for $C_{31}H_{59}NNaO_8SSi_2$ 684.3397; Found 684.3391.

For Compound 9 and *ent*-9

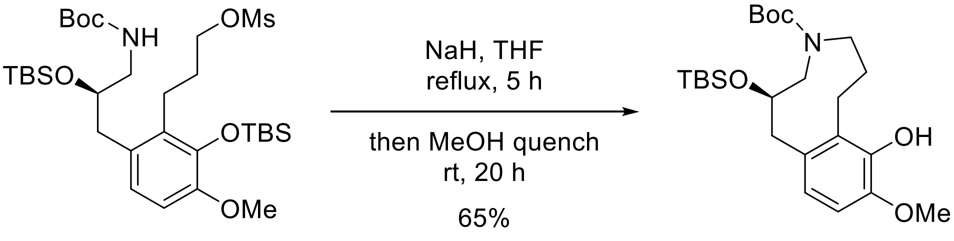

20
***ent*-20**

9, 50%
***ent*-9**, 65%

To a solution of mesylate ***ent*-20** (7.26 g, 10.97 mmol) in THF (440 mL) cooled to 0 °C was carefully added NaH (3.51 g, 41.24 mmol, 8.0 equiv.). The reaction was heated to reflux temperature and stirred for 5 h. After the reaction mixture was cooled to room temperature, MeOH (44 mL) was slowly added over 10 min and the mixture was stirred at room temperature for 20 hours. The solvent was removed in vacuo, and the residue was diluted with water (20 mL) and Et_2O (20 mL). The layers were separated and the aqueous phase was extracted with Et_2O (2 x 50 mL). The combined organic extracts were washed with brine (50 mL), dried over $MgSO_4$, and concentrated in vacuo. The crude residue was purified by silica gel column chromatography (EtOAc/Hex: 10:90 to 15:85) to afford *ortho*-methoxyphenol ***ent*-9** (3.20 g, 7.09 mmol, 65 %) as a white solid.

For **9**: [α]$^{20}_{D}$ =−60.0 (*c* 1.0, DCM); for ***ent*-9**: [α]$^{20}_{D}$ = +70.0 (*c* 1.0, DCM).
m.p.: 50.2–51.3 °C.
^{1}H NMR (400 MHz, $CDCl_3$, rotamers): *δ* 6.89 (d, *J* = 8.4 Hz, 1H), 6.70 and 6.69 (rotamers, d, *J* = 8.4 Hz, 1H), 5.66 (s, 1H), 4.58 and 4.44 (rotamers, m, 1H), 3.86 (s, 3H), 3.72–3.38 (rotamers, m, 2H), 3.06 (t, *J* = 16.6 Hz, 1H), 2.93–2.62 (m, 3H), 2.50–2.18 (m, 2H), 2.18–1.99 (m, 1H), 1.99–1.80 (m, 1H), 1.44 and 1.39 (rotamers, s, 9H), 0.94 and 0.93 (rotamers, s, 9H), 0.18 and 0.16 (rotamers, s, 3H), 0.13 and 0,09 (rotamers, s, 3H).
^{13}C NMR (150 MHz, $CDCl_3$, rotamers): *δ* 156.4 and 156.0 (rotamers), 144.4 and 144.3 (rotamers), 143.6 and 143.4 (rotamers), 129.8 and 129.3 (rotamers), 126.4, and 126.2 (rotamers), 122.3, 108.0, and 107.9 (rotamers), 79.4 and 79.0 (rotamers), 70.7 and 69.7 (rotamers), 58.2 and 57.2 (rotamers), 55.9 and 55.8 (rotamers), 53.0 and 52.7 (rotamers), 36.6 and 36.0 (rotamers), 28.6 and 28.4 (rotamers), 26.5 and 25.5 (rotamers), 25.9, 23.0 and 22.7 (rotamers), 18.1, and 18.1 (rotamers), −4.5, −4.6 and −4.6 (rotamers).
IR (KBr): *v* 3549, 2955, 2930, 2857, 1695, 1617, 1493, 1473, 1363 cm^{-1}.
HRMS (ESI) m/z: $[M-H]^-$ Calcd for $C_{24}H_{40}NO_5Si$ 450.2675; Found 450.2672.

For Compound 21 and *ent*-21

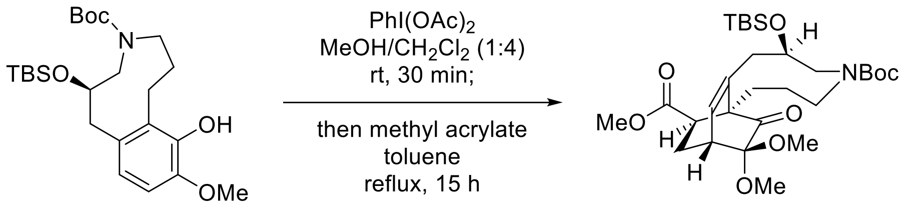

9
***ent*-9**

21, 68%
***ent*-21**, 75%

To a solution of *ortho*-methoxyphenol ***ent*-9** (1.06 g, 2.35 mmol) in $MeOH/CH_2Cl_2$ (1:4, 23.4 mL) was added $PhI(OAc)_2$ (0.91 g, 2.82 mmol). After stirring at room temperature for 30 min, methyl acrylate (3.19 mL, 35.44 mmol) and toluene (243 mL) were added. The reaction was heated to reflux and stirred for 15 h. The reaction mixture was cooled to room temperature, and the solvent was removed in vacuo. The crude residue was purified by column chromatography with SiO_2 (EtOAc/Hex: 1:9 to 2:8) to afford Diels-Alder adduct ***ent*-21** (0.99 g, 1.74 mmol, 75% yield) as a colorless liquid.

For **21**: [α]$^{20}_{D}$ = −43.0 (*c* 1.0, DCM); for ***ent*-21**: [α]$^{20}_{D}$ = +34.8 (*c* 1.0, DCM).
^{1}H NMR (400 MHz, $CDCl_3$, rotamers): *δ* 6.50 (d, *J* = 7.2 Hz, 1H), 4.33–4.14 (rotamers, m, 1H), 3.79–3.42 (rotamers, m, 2H), 3.63 and 3.62 (rotamers, s, 3H), 3.42 and 3.41 (rotamers, s, 3H), 3.24 and 3.23 (rotamers, s, 3H), 3.11 (ddd, *J* = 7.2, 3.0, 3.0 Hz, 1H), 2.69–2.61 (m, 1H), 2.58–2.31 (m, 4H), 2.28–2.18 (m, 1H), 2.18–1.76 (m, 4H), 1.58–

1.46 (m, 1H), 1.45 and 1.44 (rotamers, s, 9H), 0.91 and 0.90 (rotamers, s, 9H), 0.11 and 0.10 (rotamers, s, 3H), 0.10 and 0.07 (rotamers, s, 3H).

^{13}C NMR (100 MHz, $CDCl_3$, rotamers): δ 205.0, 173.6 and 173.6 (rotamers), 156.1 and 155.9 (rotamers), 133.5 and 133.0 (rotamers), 132.8 and 132.4 (rotamers), 93.9 and 93.9 (rotamers), 79.6 and 79.3 (rotamers), 69.2 and 68.4 (rotamers), 57.6 and 57.4 (rotamers), 53.9 and 53.3 (rotamers), 51.8, 50.1 and 49.8 (rotamers), 50.0, 49.7, 48.6 and 48.4 (rotamers), 39.0 and 38.7 (rotamers), 34.2 and 33.6 (rotamers), 28.6 and 28.5 (rotamers), 27.1, 25.9, 25.0 and 24.8 (rotamers), 24.6 and 23.7 (rotamers), 18.0 and 18.0 (rotamers), −4.6 and −4.6 (rotamers), −4.6 and −4.7 (rotamers).

IR (KBr): ν 2953, 1756, 1657, 1489 cm^{-1}.

HRMS (ESI) m/z: $[M+Na]^+$ Calcd for $C_{29}H_{49}NNaO_8Si$ 590.3125; Found 590.3122.

For Compound 7 and *ent*-7

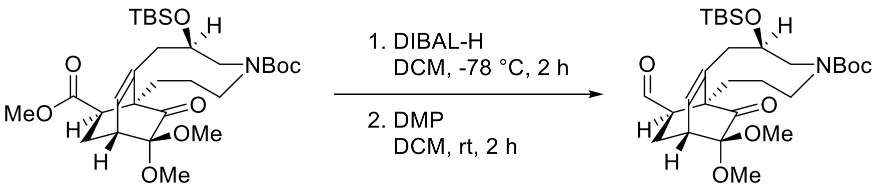

21 **ent-21** **7** **ent-7**

To a solution of methyl ester ***ent*-21** (2.75 g, 4.53 mmol) in dry DCM (45 mL) was added DIBAL-H (1.0 M in toluene, 9.1 mL, 9.1 mmol) at −78 °C. The resulting mixture was stirred for 2 h at the same temperature. The mixture was quenched by adding aqueous sodium potassium tartrate solution at −78 °C and stirred for 15 min at 0 °C. After finishing, DCM was added and extracted with a brine solution. The organic phase was washed with water, dried over $MgSO_4$, and concentrated. The crude mixture of alcohol and aldehyde was dissolved in DCM (45.0 mL) at 0 °C, and DMP (2.89 g, 6.80 mmol.) was added. The resulting mixture was stirred at room temperature for 2 h. The mixture was quenched by adding aqueous sat. $Na_2S_2O_3$ (15 mL) and sat. $NaHCO_3$ (15.0 mL) at 0 °C. After the finish, DCM was added and extracted with a brine solution. The organic phase was washed with water, dried over $MgSO_4$, and concentrated to afford the desired crude aldehyde ***ent*-7** (2.47 g, quantitative), which was directly used for the next step without further purification.

^{1}H NMR (400 MHz, $CDCl_3$, rotamers): δ 9.39 and 9.34 (rotamers, d, J = 3.0 Hz, 1H), 6.52 and 6.50 (rotamers, d, J = 6.2 Hz, 1H), 4.34–4.13 (rotamers, m, 1H), 3.81–3.46 (rotamers, m, 2H), 3.45 and 3.44 (rotamers, s, 3H), 3.23 and 3.22 (rotamers, s, 3H), 3.19–3.10 (m, 1H), 2.65–2.53 (m, 1H), 2.53–2.38 (m, 2H), 2.38–2.26 (m, 2H), 2.26–1.94 (m, 5H), 1.76–1.56 (m, 1H), 1.45 and 1.44 (rotamers, s, 9H), 0.90 and 0.90

(rotamers, s, 9H), 0.10 and 0.09 (rotamers, s, 3H), 0.09 and 0.06 (rotamers, s, 3H).
IR (KBr): *v* 2956, 2928, 2855, 1733, 1698, 1472, 1412, 1364, 1251, 1163, 1073 cm^{-1}.

For Compound 22 and *ent*-22

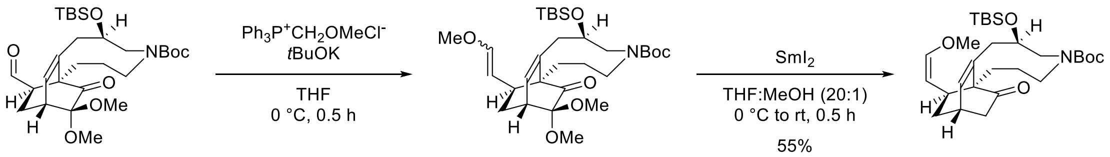

To a solution of (methoxymethyl)triphenylphosphonium chloride (6.69 g, 19.5 mmol) in THF (18.0 mL) was added 1.0 M *t*BuOK in THF (19.5 mL, 19.5 mmol) at 0 °C. After stirring at room temperature for 10 min, the mixture was cooled to 0 °C, and a solution of crude aldehyde ***ent*-7** (3.50 g, 6.50 mmol) in THF (25 mL) was added. The reaction mixture was stirred for 30 min at 0 °C, then diluted with brine (50 mL) and extracted with diethyl ether (3 × 50 mL). The combined organic extracts were dried over $MgSO_4$ and concentrated in vacuo. The crude residue was purified by silica gel column chromatography with SiO_2 (EtOAc/Hex: 1:9) to afford *E*/*Z* mixture (≈ 1:5) of enol ether ***ent*-SI-5** (1.95 g, 3.44 mmol, 53% yield) as a colorless foam.

To a solution of enol ether ***ent*-SI-5** (400 mg, 0.706 mmol) in THF/MeOH (20:1, 7 mL) was slowly added 0.1 M SmI_2 in THF (56.55 mL, 5.655 mmol, 8.0 equiv.) at 0 °C over 15 min. After stirring at room temperature for 30 min, the deep blue mixture was diluted with sat. potassium sodium bicarbonate (50 mL) and stirred for another 30 min. The resulting solution was extracted with EA (3 × 100 mL). The combined organic extracts were washed with brine (10 mL), dried over $MgSO_4$, and concentrated in vacuo. The crude residue was purified by column chromatography with SiO_2 (EtOAc/Hex: 5:95 to 15:85) to afford ketone ***ent*-22** (197 mg, 0.390 mmol, 55% yield) as a colorless foam.

^{1}H NMR (400 MHz, $CDCl_3$, rotamers): δ = 6.47 (d, *J* = 6.8 Hz, 1H), 5.85 and 5.84 (rotamers, d, *J* = 5.6 Hz, 1H), 4.31–4.11 (rotamers, m, 1H), 3.87 and 3.82 (rotamers, dd, *J* = 10.2, 6.2 Hz, 1H), 3.79–3.57 (rotamers, m, 1H), 3.68–3.38 (rotamers, m, 1H), 3.53 (s, 3H), 2.94–2.81 (m, 2H), 2.57–2.29 (m, 4H), 2.22–1.86 (m, 5H), 1.86–1.75 (m, 2H), 1.46 and 1.45 (rotamers, s, 9H), 1.14–1.00 (m, 1H), 0.90 and 0.89 (rotamers, s, 9H), 0.10 and 0.07 (rotamers, s, 6H).
HRMS (ESI) m/z: $[M+Na]^+$ Calcd for $C_{28}H_{47}NNaO_5Si$ 528.3121; Found 528.3118.

For Compound SI-6 and *ent*-SI-6

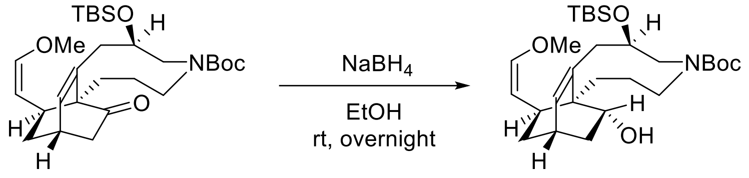

22
***ent*-22**

SI-6, 73%
***ent*-SI-6**, 79%

To a solution of ketone ***ent*-22** (0.88 g, 1.73 mmol) in ethanol (5.0 mL) was added $NaBH_4$ (198 mg, 5.21 mmol). After stirring at room temperature overnight, the reaction mixture was diluted with sat. NH_4Cl (30 mL) and extracted with CH_2Cl_2 (3 x 30 mL). The combined organic extracts were washed with brine (30 mL), dried over $MgSO_4$, and concentrated in vacuo. The crude residue was purified by silica gel column chromatography with SiO_2 (EtOAc/Hex: 1:9 to 5:5) to afford alcohol ***ent*-SI-6** (0.69 g, 1.37 mmol, 79%) as colorless foam.

^{1}H NMR (600 MHz, $CDCl_3$, rotamers): δ = 6.06 and 6.05 (rotamers, d, J = 6.2 Hz, 1H), 5.78 (d, J = 6.0 Hz, 1H), 4.33–4.14 (rotamers, m, 1H), 3.95–3.36 (m, 4H), 3.53 (s, 3H), 3.13 (ddd, J = 9.8, 4.6, 1H), 3.00–2.88 (m, 1H), 2.65–2.24 (m, 4H) 2.03–1.54 (m, 7H), 1.47 (s, 9H), 1.21–1.11 (m, 1H), 0.88 (s, 9H), 0.07 and 0.06 (rotamers, s, 3H), 0.06 and 0.05 (rotamers, s, 3H).

HRMS (ESI) m/z: $[M+Na]^+$ Calcd for $C_{28}H_{49}NNaO_5Si$ 530.3277; Found 530.3284.

For Compound 24 and *ent*-24

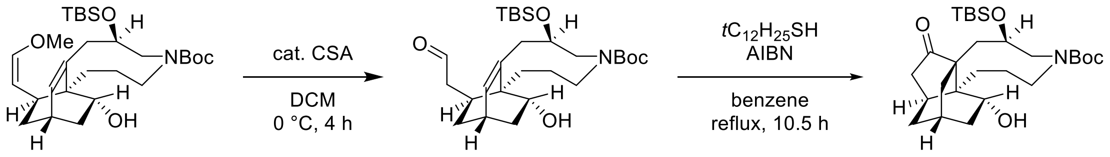

SI-6
***ent*-SI-6**

23
***ent*-23**

24, 50% (2 steps)
***ent*-24**, 52% (2 steps)

To a solution of enol ether ***ent*-SI-6** (173 mg, 0.340 mmol) in DCM (3 mL) was added camphorsulfonic acid (7.91 mg, 0.034 mmol, 0.1 equiv.). After stirring at 0 °C for 4 h, the reaction was diluted with sat. $NaHCO_3$ (5 mL). The layers were separated, and the aqueous phase was extracted with DCM (2 × 10 mL). The combined organic extracts were washed with brine (50 mL), dried over $MgSO_4$, filtered, and concentrated in vacuo to afford aldehyde ***ent*-23** as colorless oil.

A solution of crude aldehyde ***ent*-23** (170 mg, 0.344 mmol), *tert*-dodecanethiol (0.204 mL, 0.860 mmol), and AIBN (56 mg, 0.344 mmol) in benzene (5.0 mL) under argon atmosphere was heated to reflux for 10.5 h with stirring. After the reaction was cooled to room temperature, the resulting solution was purified by column chromatography with SiO_2 (EtOAc/Hex: 20:80 to 25:75) to afford 5/6/6/9 tetracyclic intermediate ***ent*-24** (89 mg, 0.18 mmol, 52%) as colourless gum.

For **24**: $[\alpha]^{20}_D = -49.9$ (*c* 1.0, DCM); for ***ent*-24**: $[\alpha]^{20}_D = +39.2$ (*c* 1.0, DCM).
^{1}H NMR (600 MHz, $CDCl_3$, rotamers): δ 4.18–3.83 (rotamers, m, 1H), 4.14 (d, *J* = 10.8 Hz, 1H), 3.45–3.30 (m, 1H), 3.27–3.16 (m, 1H), 3.11 and 3.02 (rotamers, dd, *J* = 13.2, 13.2 Hz, 1H), 3.02 and 2.82 (rotamers, dd, *J* = 12.6, 12.6 Hz, 1H), 2.52–2.39 (rotamers, m, 1H), 2.28–1.84 (m, 8H), 1.84–1.56 (m, 6H), 1.49 and 1.47 (rotamers, s, 9H), 1.19-1.11 (m, 1H), 0.90 (s, 9H), 0.21 and 0.21 (rotamers, s, 3H), 0.11 and 0.09 (rotamers, s, 3H).
^{13}C NMR (100 MHz, $CDCl_3$, rotamers): δ 220.4 and 219.7 (rotamers), 156.3, 80.1 and 79.6 (rotamers), 67.5 and 67.3 (rotamers), 65.8 and 65.3 (rotamers), 55.6 and 55.5 (rotamers), 54.9 and 54.5 (rotamers), 49.4 and 48.8 (rotamers), 45.8, 44.9, 44.6 and 44.1 (rotamers), 44.5 and 43.7 (rotamers), 40.4 and 40.0 (rotamers), 37.1, and 36.3 (rotamers), 35.3 and 35.1 (rotamers), 29.7, 28.5 and 28.4 (rotamers), 26.0 and 25.9 (rotamers), 23.3 and 22.1 (rotamers), 18.0, −4.3 and −4.6 (rotamers), −4.5 and −4.7 (rotamers).
IR (KBr): *v* 3450, 2924, 2857, 1724, 1669, 1470, 1415, 1251, 1166, 1050 cm^{-1}.
HRMS (ESI) m/z: $[M+Na]^+$ Calcd for $C_{27}H_{47}NNaO_5Si$ 516.3121; Found 516.3121.

For Compound 25 and *ent*-25

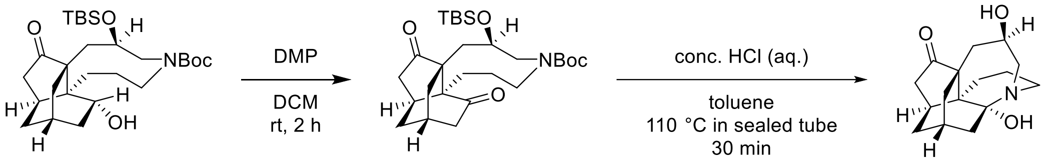

24
***ent*-24**

6, 69%
***ent*-6**, 78%

25, 48%
***ent*-25**, 44%

To a solution of alcohol ***ent*-24** (240 mg, 0.545 mmol) in DCM (1.5 mL) was added Dess-Martin periodinane (463 mg, 1.091 mmol). After stirring at room temperature for 2 h, the above mixture was added sat. $Na_2S_2O_3$ (2.5 mL) and sat. $NaHCO_3$ (2.5 mL) followed by stirring for 30 min. The resulting solution was extracted with DCM (3 x 5 mL). The combined organic extracts were washed with brine (5 mL), dried over $MgSO_4$, and concentrated in vacuo. The crude residue was purified by column chromatography with SiO_2 (EtOAc/Hex: 2:8 to 3:7) to afford dione ***ent*-6** (210 mg, 0.427 mmol, 78% yield) as white foam.
For **6**: $[\alpha]^{20}_D = -60.4$ (*c* 1.0, MeOH); for ***ent*-6**: $[\alpha]^{20}_D = +33.1$ (*c* 1.0, MeOH).
^{1}H NMR (600 MHz, $CDCl_3$, rotamers): δ 4.56–4.40 (rotamers, m, 1H), 3.73-3.44 (rotamers, m, 2H), 3.16–2.90 (rotamers, m, 1H), 2.73–2.70 (m, 1H), 2.62 (dd, *J* = 18.6, 7.8 Hz, 1H), 2.56–2.55 (m, 1H), 2.47 (dd, *J* = 14.4, 10.8 Hz, 1H), 2.33–2.29 (m, 1H), 2.20–2.07 (m, 5H), 1.91–1.67 (m, 6H), 1.46 and 1.40 (rotamers, s, 9H), 1.14-1.11 (m, 1H), 0.88 and 0.85 (rotamers, s, 9H), 0.15 and 0.12 (rotamers, s, 6H).

^{13}C NMR (100 MHz, $CDCl_3$, rotamers): δ 217.2, 216.6 and 215.4 (rotamers), 201.2, 155.6 and 155.1 (rotamers), 80.1 and 79.1 (rotamers), 67.9 and 67.8 (rotamers), 58.3 and 57.3 (rotamers), 54.4, 53.1 and 51.7(rotamers), 48.1 and 47.8 (rotamers), 46.1 and 45.9 (rotamers), 43.9 and 43.2 (rotamers), 42.4 and 42.2 (rotamers), 41.0, 39.6, 36.3 and 35.7 (rotamers), 29.6, and 29.1 (rotamers), 28.6, 28.4 and 28.1 (rotamers), 25.8 and 25.3 (rotamers), 17.9, −4.3 and −4.6 (rotamers), −5.1.
IR (KBr): v 2950, 2925, 2859, 1726, 1675, 1475, 1415, 1356, 1251, 1173, 1040 cm^{-1}.
HRMS (ESI) m/z: $[M+Na]^+$ Calcd for $C_{27}H_{45}NNaO_5Si$ 514.2964; Found 514.2965.

A solution of dione ***ent*-6** (210 mg, 0.427 mmol), conc. HCl (aq., 0.25 mL) and toluene (2.0 mL) were heated to 110 °C in a sealed tube. After stirring at 110 °C for 30 min, the reaction mixture was cooled to room temperature, diluted with sat. $NaHCO_3$ (5 mL) and extracted with CH_2Cl_2 (3 X 5 mL). The combined organic extracts were washed with brine (5 mL), dried over $MgSO_4$, filtered, and concentrated in vacuo. The crude residue was purified by column chromatography with neutral Al_2O_3 (100 % Ea to 5% MeOH/DCM) to afford product ***ent*-25** (52 mg, 0.187 mmol, 44% yield) as white foam.

For **25**: $[\alpha]^{20}_D = -39.9$ (c 1.0, MeOH); for ***ent*-25**: $[\alpha]^{20}_D = +22.9$ (c 1.0, MeOH).
^{1}H NMR (600 MHz, $CDCl_3$): δ 4.41 (ddd, J = 9.0, 9.0, 9.0 Hz, 1H), 3.82 (ddd, J =13.8, 13.8, 6.0 Hz, 1H), 3.47 (dd, J = 15.6, 10.8 Hz, 1H), 3.21 (dd, J = 15.3, 6.9 Hz, 1H), 2.79 (dd, J = 14.1, 6.9 Hz, 1H), 2.53 (d, J =15.0 Hz, 1H), 2.46 (dd, J = 19.2, 7.8 Hz, 1H), 2.28 (dd, J = 16.5, 9.3 Hz, 1H), 2.22 (dd, J = 8.7, 8.7 Hz, 1H), 2.18–2.04 (m, 2H), 2.03 (d, J = 19.2 Hz, 1H), 2.01–1.95 (m, 2H), 1.83 (d, J = 15.0 Hz, 1H), 1.74 (d, J = 15.0 Hz, 1H), 1.69–1.49 (m, 3H), 1.38–1.29 (m, 2H).
^{13}C NMR (150 MHz, $CDCl_3$): δ 221.4, 84.6, 67.9, 54.4, 50.3, 45.5, 43.9, 43.6, 43.0, 39.0, 36.9, 35.8, 34.8, 26.4, 26.3, 20.6.
IR (KBr): v 3440, 2921, 2861, 1728, 1455, 1408, 1209, 1165, 1068 cm^{-1}.
HRMS (ESI) m/z: $[M+H]^+$ Calcd for $C_{16}H_{24}NO_3$ 278.1756; Found 278.1760.

For (−)-Palhinine B (1) and (+)-Palhinine B (*ent*-1)

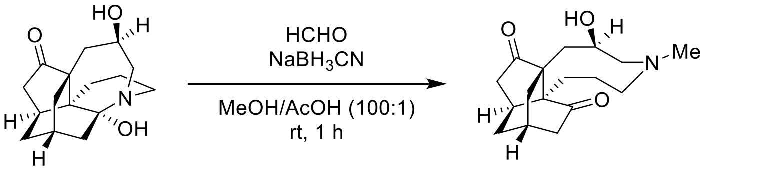

25
***ent*-25**

(-)-palhinine B (**1**, 70%) from D-tyrosine
(+)-palhinine B (***ent*-1**, 82%) from L-tyrosine

The hemiaminal **25** (15 mg, 0.054 mmol) was dissolved in AcOH/MeOH (1:100, 0.5 mL), 37 wt.% formaldehyde (0.022 mL, 0.270 mmol), and $NaBH_3CN$ (4 mg, 0.059

mmol) was added. After stirring at room temperature for 1 h, the reaction mixture was diluted with sat. Na_2CO_3 (5 mL) and extracted with CH_2Cl_2 (3 X 5 mL). The combined organic extracts were dried over $MgSO_4$, filtered, and concentrated in vacuo. The crude residue was purified by column chromatography with neutral Al_2O_3 (EtOAc/Hex: 2:8 to 4:6) to afford palhinine B (**1**, 11 mg, 0.038 mmol, 70% yield) as a white solid.

For (−)-palhinine B (**1**): $[\alpha]^{20}_D$ = −1.9 (*c* 0.13, MeOH); for (+)-palhinine B (***ent*-1**): $[\alpha]^{20}_D$ = +3.0 (*c* 0.13, MeOH).[2]

^{1}H NMR (600 MHz, $CDCl_3$): δ 4.09 (m, 1H), 2.80 (dd, *J* =15.0, 3.0 Hz, 1H), 2.60 (dd, *J* = 18.9, 8.4 Hz, 1H), 2.55–2.41 (m, 5H), 2.28–2.19 (m, 4H), 2.18 (s, 3H), 2.14 (dd, *J* = 8.4, 8.4 Hz, 1H), 2.11 (ddd, *J* = 12.3, 6.0, 2.1 Hz, 1H), 1.96–1.88 (m, 1H), 1.74 (ddd, *J* = 14.4, 3.0, 3.0 Hz, 1H), 1.69 (dd, *J* = 16.2, 2.4 Hz, 1H), 1.67 (m, 1H), 1.56–1.48 (m, 1H), 1.44–1.36 (m, 1H), 1.23 (ddd, *J* = 15.0, 4.2, 4.2 Hz, 1H).

^{13}C NMR (150 MHz, $CDCl_3$): δ 219.4, 210.9, 71.7, 62.4, 58.3, 57.0, 54.5, 47.3, 45.7, 43.5, 42.1, 39.4, 39.2, 35.9, 29.4, 28.6, 25.8.

IR (KBr): ν 3374, 2920, 2853, 1735, 1465, 1087, 1045 cm^{-1}.

HRMS (ESI) m/z: $[M+H]^+$ Calcd for $C_{17}H_{26}NO_3$ 292.1912; Found 292.1911.

For (−)-Palhinine C (2)

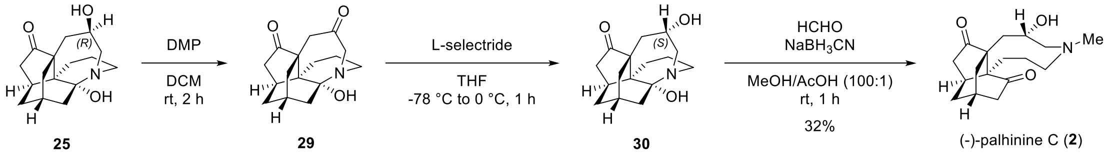

To a solution of alcohol **25** (18 mg, 0.065 mmol) in CH_2Cl_2 (1.5 mL) was added Dess-Martin periodinane (55 mg, 0.129 mmol, 2.0 equiv.). After stirring at room temperature for 2 h, the above mixture was added sat. $Na_2S_2O_3$ (5 mL) and sat. $NaHCO_3$ (5 mL) followed by stirring for 20 min. The resulting solution was extracted with CH_2Cl_2 (3 x 5 mL). The combined organic extracts were washed with brine (5 mL), dried over $MgSO_4$, and concentrated in vacuo to give crude dione **29** (16 mg, 0.058 mmol, 89%).

The crude residue **29** (16 mg, 0.058 mmol) was dissolved in dry THF (1.0 mL). The solution was dropwise added L-Selectride (1.0 M in THF, 0.174 mL, 0.174 mmol) at −78 °C. After 5 min, the reaction mixture was warmed to 0 °C and stirred at the same temperature for 1 h. An aqueous saturated solution of potassium sodium tartrate (5 mL) was added to the mixture and the resulting mixture was vigorously stirred at the same temperature for 20 min. DCM (5 mL) was added to the mixture. The organic phase was separated, and the aqueous layer was extracted with DCM (2 × 4 mL). The combined organic extracts were washed with brine (6 mL), dried over anhydrous $MgSO_4$, filtered,

and concentrated under reduced pressure to give alcohol **30** (14 mg, 0.050 mmol, 87%).

The crude alcohol **30** (14 mg, 0.050 mmol) was then dissolved in AcOH/MeOH (1:100, 1.0 mL), a 37 wt.% formaldehyde (0.020 mL, 0.252 mmol, 5 equiv.) and $NaBH_3CN$ (3.4 mg, 0.055 mmol, 1.1 equiv.) was added. After stirring at room temperature for 1 h, the reaction mixture was diluted with sat. Na_2CO_3 (5 mL) and extracted with CH_2Cl_2 (3 X 5 mL). The combined organic extracts were dried over $MgSO_4$ and concentrated in vacuo. The crude residue was purified by column chromatography with neutral Al_2O_3 (CH_2Cl_2/MeOH = 100/0 to 100/5) to afford (−)-palhinine C (**2**, 5 mg, 32% yield) as a colorless gum which crystallized in 1/5 Ea/hex..

For (−)-palhinine C (**2**): $[\alpha]^{20}_{D} = -14$ (*c* 0.12, MeOH).[2]

m.p.: 96.1–97.4 °C

^{1}H NMR (600 MHz, $CDCl_3$): *δ* 3.89 (m, 1H), 2.59 (dd, *J* = 19.2, 7.8 Hz, 1H, overlapped), 2.59–2.54 (m, 1H, overlapped), 2.51 (dd, *J* = 13.8, 10.2 Hz, 1H, overlapped), 2.48 (ddd, *J* = 14.1, 11.7, 2.1 Hz, 1H, overlapped), 2.40–2.23 (m, 4H), 2.19 (d, *J* = 19.2 Hz, 1H), 2.15–2.03 (m, 4H), 2.00 (s, 3H, overlapped), 1.99 (dd, *J* = 15.3, 9.9 Hz, 1H, overlapped), 1.95–1.87 (m, 1H), 1.69 (ddd, *J* = 14.7, 2.7, 2.7 Hz, 1H, overlapped), 1.67–1.60 (m, 1H, overlapped), 1.49–1.41 (m, 1H), 1.42–1.36 (m, 1H), 1.13 (ddd, *J* = 15.0, 4.8, 2.4 Hz, 1H).

^{13}C NMR (150 MHz, $CDCl_3$): *δ* 217.6, 207.2, 66.8, 62.6, 57.1, 55.3, 53.3, 45.7, 44.8, 43.1, 42.7, 42.4, 36.7, 35.7, 29.8, 27.4, 24.6.

IR (KBr): *ν* 3421, 2922, 2855, 1743, 1724, 1460, 1057 cm^{-1}.

HRMS (ESI) m/z: $[M+H]^+$ Calcd for $C_{17}H_{26}NO_3$ 292.1912; Found 292.1913.

3. Natural Product Spectral Comparisons

(−)-Palhinine B ^{1}H & ^{13}C NMR Spectra Comparison:

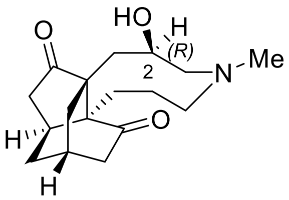

(–)-Palhinine B (**1**)

Table SI-1. Comparison of ^{1}H NMR Spectral Data of (−)-Palhinine B (1)

Natural palhinine B[3]<br>^{1}H NMR (600 MHz, CDCl$_3$)<br>δ_H (J in Hz)	Synthetic palhinine B<br>^{1}H NMR (600 MHz, CDCl$_3$)<br>δ_H (J in Hz)
4.09 (m, 1H)	4.09 (m, 1H)
2.81 (dd, J = 15.6, 2.4, 1H)	2.80 (dd, J = 15.0, 3.0, 1H)
2.60 (dd, J = 19.2, 8.4, 1H)	2.60 (dd, J = 18.9, 8.4, 1H)
2.51 (3H, overlapped)<br>2.48 (1H, overlapped)<br>2.45 (dd, J = 16.2, 6.0, 1H)	2.55–2.41 (m, 5H)
2.24 (1H, overlapped)<br>2.23 (1H, overlapped)<br>2.22 (1H, overlapped)<br>2.21 (1H, overlapped)	2.28–2.19 (m, 4H)
2.17 (s, 3H)	2.18 (s, 3H)
2.14 (t, J = 8.4, 1H)	2.14 (dd, J = 8.4, 8.4, 1H)
2.10 (ddd, J = 12.6, 6.0, 2.4, 1H)	2.11 (ddd, J = 12.3, 6.0, 2.1, 1H)
1.92 (ddd, J = 13.8, 8.4, 1.2, 1H)	1.96–1.88 (m, 1H)
1.73 (dt, J = 14.4, 2.4, 1H)	1.74 (ddd, J = 14.4, 3.0, 3.0, 1H)
1.68 (dd, J = 16.2, 1.8, 1H)	1.69 (dd, J = 16.2, 2.4, 1H)
1.68 (m, 1H)	1.67 (m, 1H)
1.51 (m, 1H)	1.56–1.48 (m, 1H)
1.40 (dd, J = 13.8, 4.2, 1H)	1.44–1.36 (m, 1H)
1.22, (dt J = 15.0, 4.2, 1H)	1.23 (ddd, J = 15.0, 4.2, 4.2, 1H)

Table SI-2. Comparison of ^{13}C NMR Spectral Data of (−)-Palhinine B (1)

Natural palhinine B[3] ^{13}C NMR (150 MHz, $CDCl_3$) δ_C	Synthetic palhinine B ^{13}C NMR (150 MHz, $CDCl_3$) δ_C	$\Delta\delta$ (ppm)
219.5	219.4	0.1
210.9	210.9	0.0
71.7	71.7	0.0
62.4	62.4	0.0
58.3	58.3	0.0
56.9	57.0	−0.1
54.5	54.5	0.0
47.3	47.3	0.0
45.6	45.7	−0.1
43.4	43.5	−0.1
42.0	42.1	−0.1
39.3	39.4	−0.1
39.1	39.2	−0.1
35.9	35.9	0.0
29.4	29.4	0.0
28.5	28.6	−0.1
25.7	25.8	−0.1

Figure SI-1. Comparison of ^{1}H NMR Spectral of (−)-Palhinine B (1)

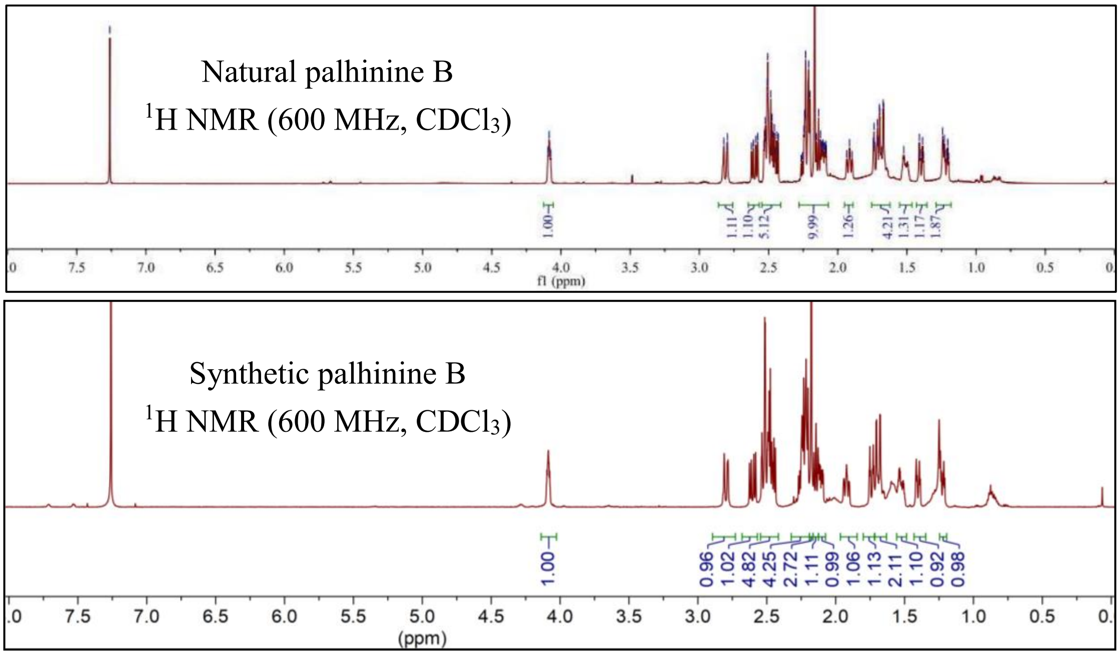

Figure SI-2. Comparison of ^{13}C NMR Spectral of (−)-Palhinine B (1)

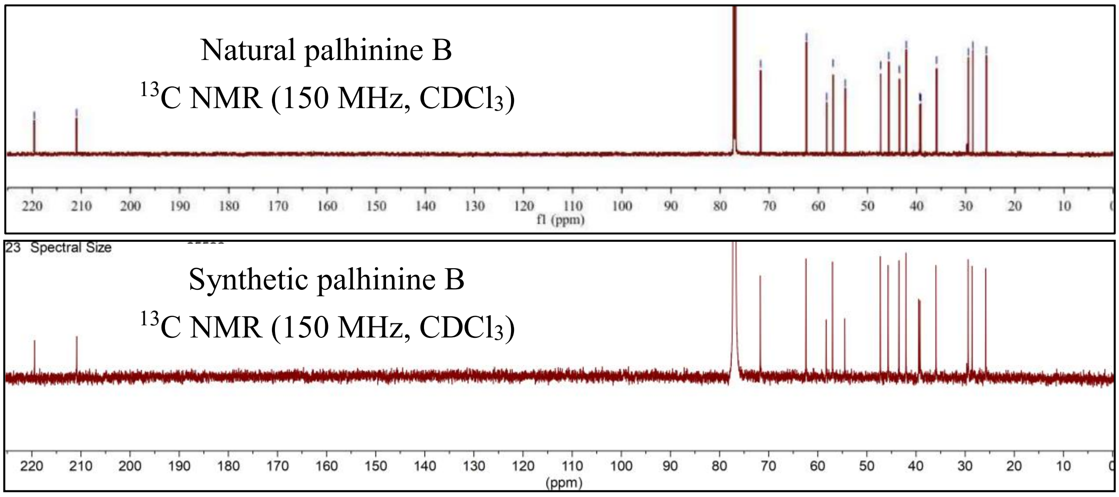

(−)-Palhinine C (2) ^{1}H & ^{13}C NMR Spectra Comparison:

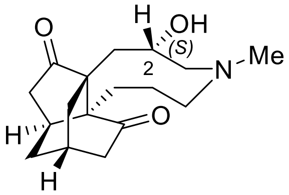

(−)-Palhinine C (**2**)

Table SI-3. Comparison of ^{1}H NMR Spectral Data of (−)-Palhinine C (2)

Natural palhinine C[3]<br>^{1}H NMR (600 MHz, CDCl$_3$)<br>δ_H (*J* in Hz)	Synthetic palhinine C<br>^{1}H NMR (600 MHz, CDCl$_3$)<br>δ_H (*J* in Hz)
3.89 (td, *J* = 10.2, 4.2, 1H)	3.89 (m, 1H)
2.60 (dd, *J* = 19.2, 8.4, 1H)	2.59 (dd, *J* = 19.2, 7.8, 1H, overlapped)
2.57 (ddd, *J* = 13.2, 4.2, 1.2, 1H)	2.59–2.54 (m, 1H, overlapped)
2.51 (dd, *J* = 13.8, 10.8, 1H)	2.51 (dd, *J* = 13.8, 10.2, 1H, overlapped)
2.47 (ddd, *J* = 13.8, 11.4, 2.4, 1H)	2.48 (ddd, *J* = 14.1, 11.7, 2.1, 1H, overlapped)
2.34 (1H, overlapped)<br>2.32 (1H, overlapped)<br>2.28 (1H, overlapped)<br>2.26 (1H, overlapped)	2.40–2.23 (m, 4H)
2.19 (d, *J* = 18.6, 1H)	2.19 (d, *J* = 19.2, 1H)
2.11 (1H, overlapped)<br>2.10 (1H, overlapped)<br>2.09 (1H, overlapped)<br>2.06 (dd, *J* = 16.8, 1.2, 1H)	2.15–2.03 (m, 4H)
2.00 (s, 3H)	2.00 (s, 3H, overlapped)
1.98 (dd, *J* = 16.2, 10.2, 1H)	1.99 (dd, *J* = 15.3, 9.9, 1H, overlapped)
1.91 (ddd, *J* = 13.8, 10.2, 3.0, 1H)	1.95–1.87 (m, 1H)
1.69 (dt, *J* = 15.0, 2.4, 1H)	1.69 (ddd, *J* = 14.7, 2.7, 2.7, 1H, overlapped)
1.65 (m, 1H)	1.67–1.60 (m, 1H, overlapped)
1.44 (m, 1H)	1.49–1.41 (m, 1H)
1.39 (dt, *J* = 13.8, 4.2, 1H)	1.42–1.36 (m, 1H)
1.12 (ddd, *J* = 15.0, 4.8, 2.4, 1H)	1.13 (ddd, *J* = 15.0, 4.8, 2.4 Hz, 1H)

Table SI-4. Comparison of ^{13}C NMR Spectral Data of (−)-Palhinine C (2)

Natural palhinine C[3] ^{13}C NMR (150 MHz, $CDCl_3$) δ_C	Synthetic palhinine C ^{13}C NMR (150 MHz, $CDCl_3$) δ_C	$\Delta\delta$ (ppm)
217.7	217.6	0.1
207.2	207.2	0.0
66.7	66.8	−0.1
62.5	62.6	−0.1
57.1	57.1	0.0
55.3	55.3	0.0
53.3	53.3	0.0
45.6	45.7	−0.1
44.8	44.8	0.0
43.0	43.1	−0.1
42.7	42.7	0.0
42.3	42.4	−0.1
36.6	36.7	−0.1
35.7	35.7	0.0
29.8	29.8	0.0
27.3	27.4	−0.1
24.6	24.6	0.0

Figure SI-3. Comparison of ^{1}H NMR Spectral of (−)-Palhinine C (2)

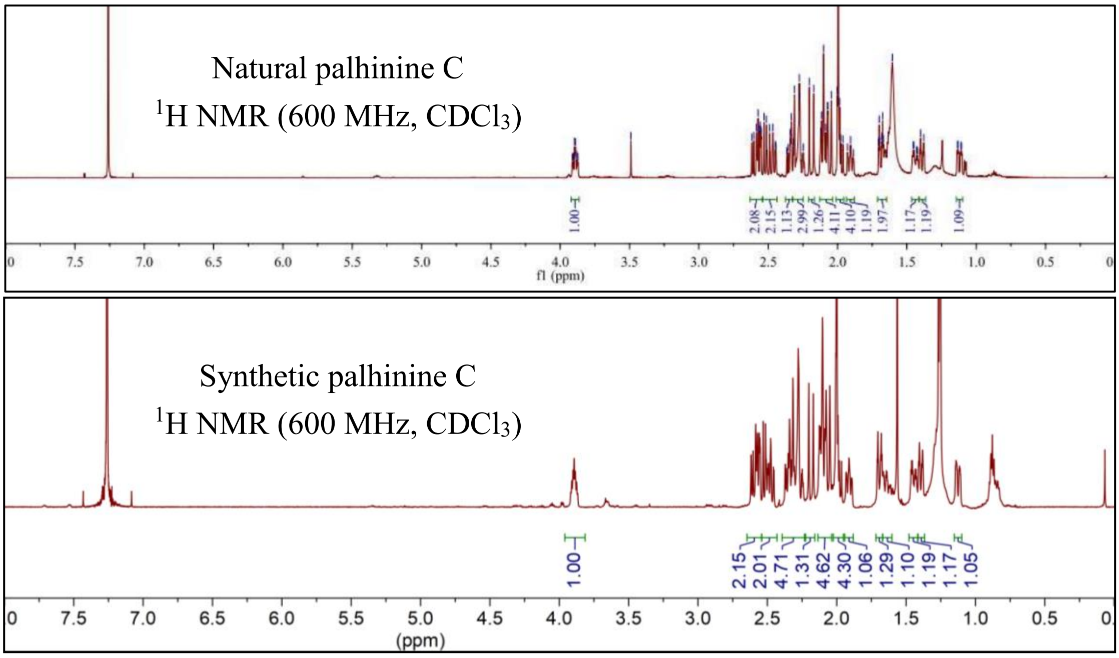

Figure SI-4. Comparison of ^{13}C NMR Spectral of (−)-Palhinine C (2)

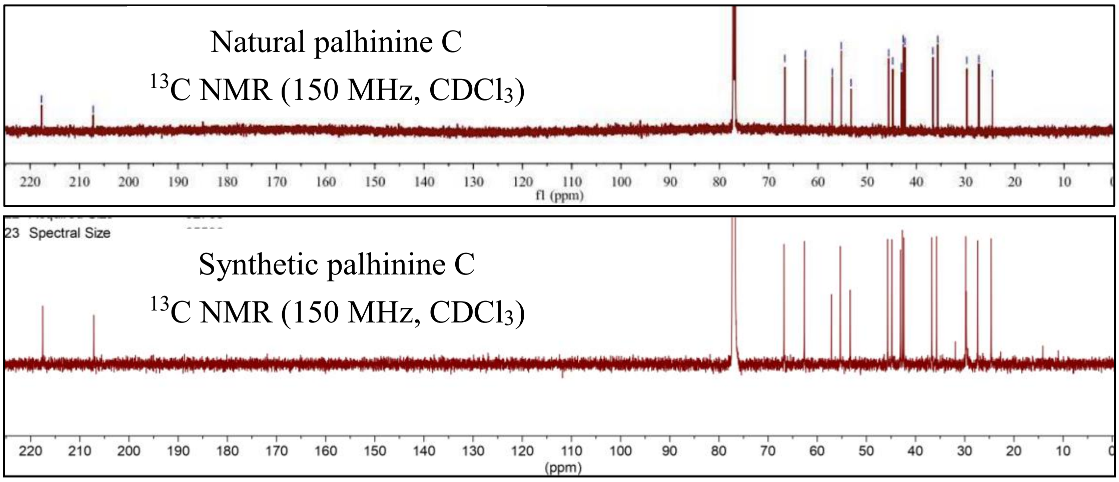

4. Computational Details

All DFT calculations were performed using the Gaussian 16 software package. Geometry optimizations and frequency calculations were carried out using the B3LYP functional with the 6-31G** basis set. Solvation free energies (G_{solv}) were computed in tetrahydrofuran with the CPCM continuum model. The electronic energies (E_{elec}) were evaluated using the same functional and the 6-311++G** basis set with Grimme's D3 empirical dispersion correction. The Gibbs free energy (G) of each species was calculated according to the following equation:

$$G = E_{elec} + ZPE + 4RT + H_{vib} - T(S_{trans} + S_{rot} + S_{vib}) + G_{solv}$$

where ZPE and H_{vib} denote the zero-point energy correction and vibrational enthalpy, respectively. All thermodynamic quantities were evaluated at T = 298 K. The calculated energies are summarized in the table below and are reported in Hartree units.

Species	E_{elec}	ZPE + 4RT + H_{vib} – TS	G_{solv}	G
31	-942.37305	0.33656	-0.01035	-942.04684
32	-942.99374	0.34644	-0.06748	-942.71479
33	-942.98365	0.34559	-0.07173	-942.70978
32a-TS	-1441.60795	0.69363	-0.05425	-1440.96856
33a-TS	-1441.58340	0.69292	-0.05706	-1440.94755
L-Selectride_rea	-499.22278	0.33247	-0.05649	-498.94680
L-Selectride_pro	-498.58876	0.32376	-0.00051	-498.26551

Species	Imaginary frequency (cm^{-1})
32a-TS	-222.29
33a-TS	-522.03

Coordinates of optimized structures:

31

O	-0.80428200	-2.67757400	-0.93867300
O	-0.84438700	3.19167800	0.25686700
N	3.17929500	0.32677100	-0.15299200
C	-1.20115600	-1.72111500	-0.29871700
C	-0.83407200	-0.27626200	-0.67041000
C	0.36677400	-0.23095900	-1.65572700
H	0.74384000	0.79649100	-1.70808800
H	-0.01619100	-0.46358700	-2.65485900
C	1.55694300	-1.19296900	-1.36084500
H	1.38600500	-1.76500600	-0.44873100
H	1.58726500	-1.95680600	-2.14242200
C	2.94947900	-0.55844900	-1.30193900

H	3.12945100	0.03436600	-2.20809600
H	3.69417000	-1.37938000	-1.31153700
C	2.90666900	-0.30562900	1.13462200
H	3.26771900	-1.34400100	1.21868700
H	3.42449500	0.27276500	1.91265700
C	1.42392800	-0.28343700	1.54006300
C	0.65828900	0.97387600	1.17394700
H	1.29010400	1.58470200	0.52968400
C	-0.72038900	0.75842600	0.51797300
C	-1.11711400	2.09091600	-0.16891000
C	-1.90204700	1.78628600	-1.43926200
H	-2.79934400	2.40738400	-1.51278300
H	-1.26773300	2.03128400	-2.29954900
C	-2.17220000	0.28139400	-1.33009600
H	-2.33033900	-0.18320400	-2.30678000
C	-3.38564000	-0.00169500	-0.38266600
H	-4.03627400	-0.76675100	-0.82094100
H	-3.99681200	0.90241900	-0.28055800
C	-2.90006400	-0.48650000	1.00102600
H	-3.74529400	-0.56740000	1.69321700
C	-2.23420300	-1.85370600	0.80384700
H	-1.71923700	-2.18359000	1.71432600
H	-2.94170100	-2.63946700	0.51858700
C	-1.83987000	0.46560000	1.57842000
H	-2.28505300	1.41061000	1.91175000
H	-1.37791900	0.00202900	2.45418600
H	0.50380900	1.54817900	2.09640900
O	0.93170900	-1.20770300	2.16553800
C	4.50893200	0.92514100	-0.21500400
H	4.63342900	1.64735100	0.59746300
H	5.32801700	0.18506200	-0.14777900
H	4.62266400	1.46244300	-1.16168100

32

O	0.74048500	-2.65491300	0.90516700
O	0.99652000	3.24738000	-0.14687200
N	-3.14747100	0.35446500	0.10046700
C	1.03501700	-1.70200700	0.18749000
C	0.78531600	-0.25825100	0.64224700

C	-0.41113300	-0.19522700	1.64165200
H	-0.78863300	0.83441900	1.68028100
H	-0.01471200	-0.40825200	2.64264200
C	-1.59620400	-1.16633600	1.36711900
H	-1.39970600	-1.71141500	0.44008900
H	-1.64358400	-1.90837500	2.17156800
C	-2.97548700	-0.52212000	1.25766100
H	-3.19975000	0.07878200	2.15400700
H	-3.73025700	-1.34129500	1.24212400
C	-2.83241900	-0.24786300	-1.21217300
H	-3.21133900	-1.28343600	-1.28845700
H	-3.38423900	0.35170900	-1.94965600
C	-1.31296600	-0.28126000	-1.68693600
C	-0.62621900	1.05256400	-1.19032800
H	-1.29192400	1.57333700	-0.49637100
C	0.72103500	0.82413900	-0.50355200
C	1.17824700	2.10203100	0.22271600
C	1.94437700	1.71119700	1.49035600
H	2.86748700	2.29325900	1.59069700
H	1.31209700	1.94988200	2.35482600
C	2.13593500	0.19811100	1.33082900
H	2.26917100	-0.30628300	2.29282800
C	3.33739400	-0.12295400	0.38015400
H	3.93241200	-0.94819700	0.79129800
H	4.00972400	0.74415400	0.31861900
C	2.83606900	-0.52141200	-1.02743100
H	3.68993300	-0.61559300	-1.71314600
C	2.08609200	-1.85378300	-0.90709700
H	1.56955300	-2.11715100	-1.83324200
H	2.75680800	-2.67233000	-0.61501500
C	1.84594900	0.52267600	-1.56087700
H	2.35630000	1.45481100	-1.84074600
H	1.36387100	0.13126500	-2.46021600
H	-0.45783300	1.74439300	-2.02586700
O	-0.63658100	-1.41942700	-1.39646900
C	-4.46325800	0.96469000	0.12447400
H	-4.55778300	1.69591700	-0.68573000
H	-5.29909300	0.23829800	0.01929400

H	-4.61445700	1.49498000	1.07390100
H	-1.43161500	-0.16363300	-2.79942000

33

O	-0.70083900	-2.31688300	-1.63163600
O	-1.07808700	3.15990600	0.72608000
N	3.16691900	0.37087100	-0.03155100
C	-0.93716400	-1.59496400	-0.66208000
C	-0.84201300	-0.06660000	-0.75974700
C	0.25549200	0.36755600	-1.78894800
H	0.52865600	1.41402500	-1.60567100
H	-0.21986700	0.34963000	-2.77694600
C	1.55778000	-0.49520700	-1.90850300
H	1.38937900	-1.48619800	-1.49590500
H	1.71526200	-0.67158400	-2.98185400
C	2.92690300	0.03525400	-1.43495600
H	3.16459200	0.94440600	-2.01010400
H	3.66097100	-0.73019100	-1.77959400
C	2.77432900	-0.60369100	1.00864800
H	3.15993200	-1.62067400	0.80160800
H	3.24129200	-0.27635000	1.94597900
C	1.23341600	-0.68991000	1.29663400
C	0.63372100	0.76111700	1.22278500
H	1.27079300	1.43113700	0.64063400
C	-0.75968000	0.72201000	0.61180100
C	-1.32736500	2.08410400	0.21694200
C	-2.30823900	1.88142200	-0.94977700
H	-3.29283500	2.30258600	-0.71572000
H	-1.92103500	2.43541200	-1.81386300
C	-2.30059500	0.36341100	-1.19380800
H	-2.47951800	0.11864500	-2.24570800
C	-3.32242200	-0.40670900	-0.28960100
H	-3.76380100	-1.23669900	-0.85594900
H	-4.15012400	0.25983900	-0.01169900
C	-2.62839700	-0.96521200	0.97922600
H	-3.37946200	-1.35731400	1.67738600
C	-1.63174300	-2.07991300	0.58117100
H	-0.87387200	-2.20557700	1.38845400
H	-2.11453200	-3.03855900	0.36196600

C	-1.80159700	0.13343900	1.64600000
H	-2.44280000	0.93047400	2.04786600
H	-1.21590500	-0.29416000	2.46547700
H	0.58589300	1.15286200	2.24484000
O	0.90979300	-1.37973300	2.39072000
C	4.53567200	0.82110700	0.12337100
H	4.69941600	1.17570900	1.14605200
H	5.29442100	0.03411300	-0.08439900
H	4.74275100	1.65542900	-0.56107900
H	0.83643800	-1.19265700	0.36281900

32a-TS

O	-3.36363900	0.39938000	-2.62787700
O	-2.38167300	-0.47248600	3.22991100
N	-0.41258700	3.05618300	0.42586200
C	-2.98648600	-0.29170500	-1.69618300
C	-3.05901900	0.19426300	-0.24292900
C	-3.16000500	1.74192700	-0.17665900
H	-2.92902600	2.06275400	0.84555600
H	-4.20628100	2.01793800	-0.35684700
C	-2.26298700	2.54052600	-1.16282100
H	-1.60896500	1.86439100	-1.71324300
H	-2.89289300	3.02879400	-1.91305200
C	-1.40392200	3.60892500	-0.49287000
H	-2.03847200	4.29937900	0.08294700
H	-0.91718800	4.21900800	-1.28426300
C	0.55214500	2.13191800	-0.19962800
H	0.77478800	2.42469500	-1.23682900
H	1.47838200	2.22197300	0.36834800
C	0.14093100	0.59902200	-0.24079600
C	-0.65691400	0.20009600	1.04341600
H	-0.78851200	1.09567900	1.65516700
C	-2.04016800	-0.44220100	0.79182100
C	-2.84427000	-0.38568000	2.11030700
C	-4.33278100	-0.23831100	1.79389900
H	-4.93729800	-0.91870000	2.40244300
H	-4.63474800	0.78489400	2.04871800
C	-4.39527200	-0.48988700	0.28499900
H	-5.26625600	-0.01693200	-0.17815400

C	-4.37219000	-2.02093500	-0.03623600
H	-5.10615700	-2.25415800	-0.81759800
H	-4.67017100	-2.59419000	0.85108000
C	-2.96847500	-2.45183800	-0.51330700
H	-2.92049500	-3.54297700	-0.61646500
C	-2.69747100	-1.77094500	-1.86172200
H	-1.64824400	-1.87032900	-2.15639200
H	-3.33123900	-2.15569200	-2.66912900
C	-1.88883900	-1.97213800	0.46624500
H	-1.90122700	-2.55387800	1.39685200
H	-0.91158600	-2.11518300	0.00740800
H	-0.09085600	-0.50590100	1.65804200
O	-0.27858800	0.13075700	-1.36268300
C	0.24701800	4.11466800	1.17519600
H	0.91741000	3.68440500	1.92547000
H	0.84524900	4.80295800	0.54322800
H	-0.50194400	4.71928200	1.70111300
B	2.74471900	-0.51315200	-0.04044500
H	1.37026700	0.08663100	0.01872700
C	3.52387200	0.64752200	-0.91317100
H	3.33591300	1.60452700	-0.40027500
C	3.10238400	-0.62692400	1.56434400
H	2.26941400	-1.17501300	2.03434900
C	2.35276300	-1.90810600	-0.81579600
H	1.56969600	-1.61517500	-1.53725100
C	4.36665200	-1.48126300	1.83796000
H	5.28244400	-0.95466400	1.54568700
H	4.47226100	-1.74201200	2.89980400
H	4.34870800	-2.42227300	1.28095800
C	3.19160500	0.72264300	2.31155000
H	2.28915200	1.30572800	2.09294800
H	4.03202700	1.31180200	1.91723000
C	3.32904700	0.62800800	3.83907800
H	4.26703900	0.14941500	4.14137100
H	3.30588200	1.62141500	4.30450300
H	2.50740100	0.04312600	4.27050500
C	5.06657700	0.50233400	-0.91601600
H	5.39042200	-0.46950500	-1.30469200

H	5.55029200	1.27200100	-1.53371500
H	5.48416200	0.60156500	0.09099200
C	2.99162500	0.83257200	-2.35988500
H	3.40014100	0.04467500	-3.00751200
H	1.90537100	0.69278700	-2.37523500
C	3.32184800	2.19287700	-2.99350800
H	2.92621000	2.26504500	-4.01438700
H	2.88109500	3.01309100	-2.41343400
H	4.40158200	2.37749500	-3.04925600
C	3.50501900	-2.53447400	-1.63732000
H	4.32333800	-2.87728000	-0.98969100
H	3.17549000	-3.40619100	-2.21918800
H	3.93458400	-1.82796600	-2.35065800
C	1.72225400	-2.99461900	0.08913500
H	2.48993700	-3.42476700	0.74792000
H	0.99395100	-2.52998400	0.76193800
C	1.02455200	-4.14662600	-0.65142600
H	1.72603200	-4.73643100	-1.25153700
H	0.53775600	-4.83696700	0.04936100
H	0.25391700	-3.76791100	-1.33355300

33a-TS

O	2.48117200	-0.01718700	2.80030000
O	2.97924600	-0.41681400	-3.19538000
N	0.75268600	3.11511400	-0.25375200
C	2.34340000	-0.56651700	1.71919100
C	2.81461200	0.04588700	0.39251700
C	3.21543000	1.55699600	0.50186300
H	3.16716500	1.98799600	-0.50292300
H	4.27201000	1.57780800	0.79445200
C	2.45705500	2.51752800	1.46464400
H	1.82599300	1.96291200	2.15275300
H	3.19186800	3.01509600	2.10976700
C	1.64617900	3.61746900	0.77203700
H	2.33189300	4.32379900	0.27918000
H	1.11823600	4.20459200	1.55617600
C	-0.19397800	2.07869800	0.17956000
H	0.23458100	1.52241500	1.01444600
H	-1.14095200	2.51745500	0.53014700

C	-0.47719000	1.11958500	-1.00210200
C	0.85430600	0.47284200	-1.57293900
H	1.31742300	1.35970300	-2.01258600
C	2.00303900	-0.36477600	-0.92562800
C	3.14082400	-0.37452300	-1.99444300
C	4.50483300	-0.39354400	-1.30968000
H	5.19215100	-1.07169300	-1.82386700
H	4.93647300	0.61293100	-1.35333200
C	4.14754500	-0.80480900	0.11727200
H	4.91523000	-0.52009300	0.84253700
C	3.86545200	-2.33982900	0.18855300
H	4.37474200	-2.77926000	1.05499800
H	4.27640800	-2.83714000	-0.69848500
C	2.35157000	-2.59981500	0.30256900
H	2.14189600	-3.67535000	0.26068600
C	1.89394900	-2.01721900	1.63977600
H	0.80334500	-2.05457200	1.73585500
H	2.31461800	-2.53969100	2.50656500
C	1.57601200	-1.86803400	-0.80230900
H	1.70158500	-2.35781200	-1.77457200
H	0.51026400	-1.90404900	-0.57396200
H	0.52402600	-0.12105900	-2.42902100
O	-1.17324300	1.57682800	-1.96628300
C	0.10716700	4.17973100	-1.00616300
H	-0.52370400	3.72536800	-1.77218500
H	-0.52586400	4.83197900	-0.36901100
H	0.86766600	4.81292900	-1.48168200
B	-2.54627100	-0.51516500	0.16525200
H	-1.22458300	0.13934900	-0.31396900
C	-3.58408000	0.21014900	-0.87258700
H	-3.03437300	0.26535400	-1.82581300
C	-2.18618400	-2.10242800	-0.12026200
H	-1.22604600	-2.30785700	0.38452800
C	-2.60840200	-0.02323600	1.73680400
H	-2.49216400	1.07115300	1.72070100
C	-3.18305900	-3.09825500	0.52265200
H	-4.19953200	-2.97275000	0.13426600
H	-2.89743000	-4.14477400	0.34775700

H	-3.23420000	-2.96588400	1.60726500
C	-1.96342500	-2.42782800	-1.61766500
H	-1.28465400	-1.68033500	-2.04342700
H	-2.90338600	-2.30229600	-2.16806900
C	-1.41154500	-3.82884100	-1.92729400
H	-2.11912000	-4.62150600	-1.65957500
H	-1.19012900	-3.93974300	-2.99600400
H	-0.48234000	-4.02654000	-1.37801800
C	-4.88944300	-0.57978100	-1.14019000
H	-5.48160500	-0.71165200	-0.22493300
H	-5.53733500	-0.06293500	-1.86202300
H	-4.70529100	-1.57813000	-1.54653000
C	-3.95467400	1.67917400	-0.54124900
H	-4.75375800	1.70386000	0.21617400
H	-3.09639500	2.18699300	-0.09582400
C	-4.38300700	2.50731300	-1.76053400
H	-4.67283600	3.52752800	-1.47410100
H	-3.54206000	2.57209600	-2.45664700
H	-5.23709600	2.06496700	-2.28843200
C	-3.98485800	-0.27273300	2.40264400
H	-4.80217900	0.11699700	1.79007600
H	-4.18011100	-1.33994000	2.56000800
H	-4.06314300	0.21338000	3.38497400
C	-1.46780400	-0.55405000	2.62858200
H	-1.55813600	-1.64469800	2.74492500
H	-0.52056100	-0.38527700	2.10505300
C	-1.34817100	0.08102900	4.02213900
H	-2.20573100	-0.15676400	4.66145700
H	-0.44323700	-0.26246400	4.53721600
H	-1.28779500	1.17391200	3.94738700

L-Selectride_rea

B	-0.00007300	-0.00006900	0.95494100
H	-0.00019900	0.00003400	2.19578000
C	0.62491000	-1.50308200	0.53275900
H	1.72474200	-1.43858700	0.64290700
C	0.98911000	1.29267600	0.53256200
H	0.38336700	2.21296200	0.64271300
C	-1.61411900	0.21017800	0.53249100

H	-2.10824300	-0.77447600	0.64302100
C	2.15669600	1.42690900	1.53238700
H	2.79393300	0.53081900	1.50921200
H	2.81604700	2.29158700	1.35153800
H	1.77368200	1.51192500	2.55471600
C	1.53299600	1.31189700	-0.91590600
H	0.71573400	1.11301400	-1.61863400
H	2.24544900	0.48305400	-1.04656200
C	2.21806000	2.61642500	-1.36224800
H	3.10624200	2.84436400	-0.76205600
H	2.53794400	2.57222300	-2.41291200
H	1.53324400	3.46898500	-1.26293400
C	0.15709400	-2.58114500	1.53262900
H	-0.93762800	-2.68424900	1.50979800
H	0.57559000	-3.58475600	1.35163700
H	0.42272100	-2.29215000	2.55489000
C	0.36965600	-1.98366200	-0.91571900
H	-0.70424900	-2.18683300	-1.04633900
H	0.60551300	-1.17620200	-1.61831400
C	1.15750500	-3.22869100	-1.36242200
H	0.95897000	-3.48373100	-2.41300100
H	2.23819200	-3.06123300	-1.26360100
H	0.91162500	-4.11199800	-0.76212600
C	-2.31371300	1.15465100	1.53222400
H	-1.85567000	2.15428200	1.50904900
H	-3.39214000	1.29397100	1.35137800
H	-2.19607400	0.78040900	2.55456800
C	-1.90290200	0.67130400	-0.91603000
H	-1.54127300	1.70265700	-1.04694900
H	-1.32228500	0.06283400	-1.61882700
C	-3.37528100	0.61240100	-1.36211500
H	-4.01661800	1.26764200	-0.76179600
H	-3.49709800	0.91156600	-2.41275400
H	-3.77123000	-0.40693300	-1.26277600

L-Selectride_pro

B	-0.00072600	-0.00066900	0.35008600
C	-1.48820900	-0.57526500	0.34892000
H	-1.46793100	-1.67542300	0.34793200

C	1.24088300	-1.00117500	0.35176600
H	2.18310000	-0.43288600	0.35394800
C	0.24504700	1.57499100	0.35049400
H	-0.71797000	2.10734800	0.34839200
C	1.22984700	-1.87267300	1.63031900
H	0.33696000	-2.50665000	1.67558500
H	2.10337300	-2.53133400	1.68109100
H	1.24296800	-1.25790000	2.53756600
C	1.24126300	-1.86755700	-0.93761600
H	1.13570600	-1.21380300	-1.81446800
H	0.35679300	-2.51821900	-0.93982600
C	2.49973800	-2.72375400	-1.13052600
H	2.62019400	-3.46204600	-0.33180800
H	2.45877400	-3.27155100	-2.07811200
H	3.40140000	-2.10085200	-1.14540300
C	-2.23630000	-0.13304600	1.62927500
H	-2.33799600	0.95714700	1.67742600
H	-3.24373700	-0.55962700	1.67968000
H	-1.71004700	-0.45451400	2.53533300
C	-2.23885700	-0.13806900	-0.93875400
H	-2.35795900	0.95345700	-0.93826800
H	-1.62098600	-0.37253400	-1.81663100
C	-3.61100800	-0.79673300	-1.13237600
H	-4.06500900	-0.48415500	-2.07887000
H	-3.52455700	-1.88918900	-1.15002400
H	-4.30956600	-0.53252600	-0.33253500
C	1.00027300	2.00112600	1.63209800
H	1.99554000	1.54473800	1.68105300
H	1.13374900	3.08693100	1.68359300
H	0.45789700	1.70496500	2.53726300
C	1.00095900	2.00727600	-0.93581100
H	2.00595900	1.56494400	-0.93399400
H	0.49041700	1.58974400	-1.81469600
C	1.11649000	3.52504400	-1.12863200
H	1.69359200	3.99771700	-0.32796300
H	1.61515500	3.76244600	-2.07449100
H	0.12705700	3.99613500	-1.14714200

5. References

(1) (a) Yu, K.; Zhang, Z.; Huo, X.; Wang, X.; Liu, D.; Zhang, W. Synthesis of D-(*R*)-Tyrosine by Catalytic Asymmetric Hydrogenation and its Practical Application. *Chin. J. Org. Chem.* **2013**, *33*, 1932–1938. (b) Bogucki D. E.; Charlton. J. L. A Non-Enzymatic Synthesis of (*S*)-(−)-Rosmarinic Acid and a Study of a Biomimetic Route to (+)-Rabdosiin. *Can. J. Chem*. **1997**, *75*, 1783–1794.

(2) Ranganathan, S.; Tamilarasu, N. The Crafting of Peptide Segments with Cu^{II} Uptake Potential. *Tetrahedron Lett.* **1994**, *35*, 447–450.

(3) Dong, L.-B.; Gao, X.; Liu, F.; He, J.; Wu, X.-D.; Li, Y.; Zhao, Q.-S. Isopalhinine A, a Unique Pentacyclic *Lycopodium* Alkaloid from *Palhinhaea cernua*. *Org. Lett.* **2013**, *15*, 3570–3573.

6. X-Ray Crystal Data

The sample of (−)-palhinine B (**1**) for X-ray analysis was obtained via slow evaporation in 1/5:EA/Hex. The thermal ellipsoids are shown at the 30% probability level.

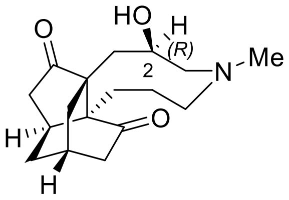

(–)-Palhinine B (**1**)

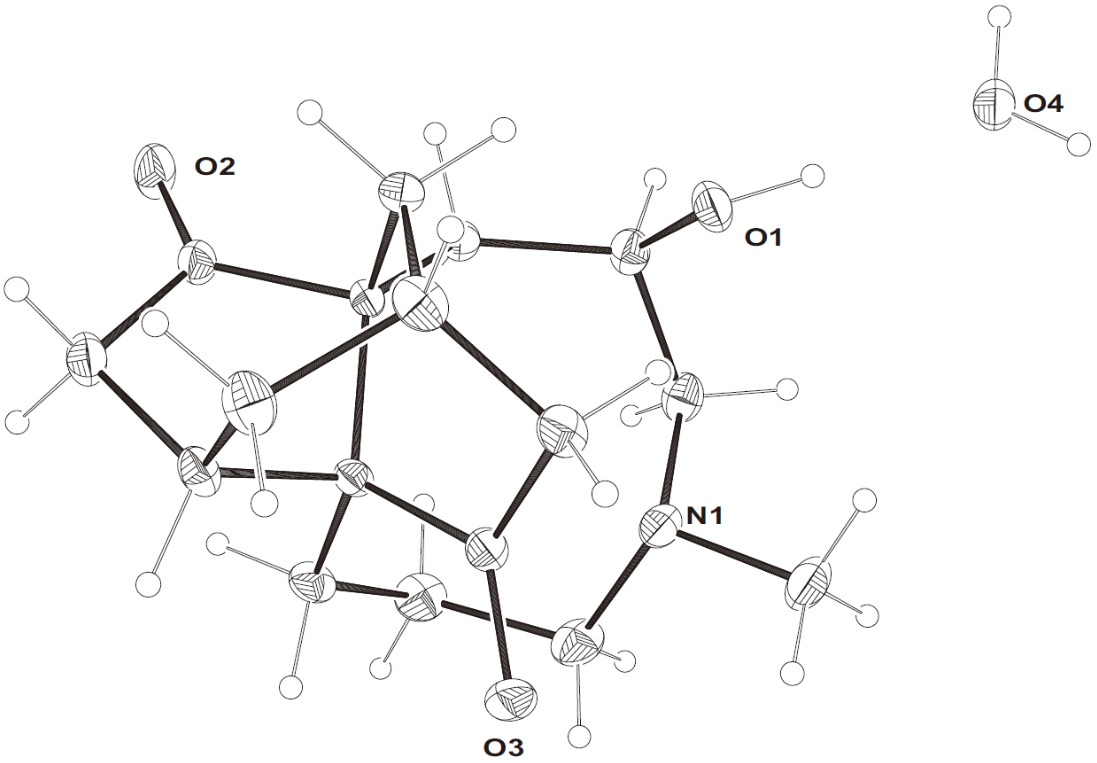

Table SI-5. Crystal data and structure refinement for (−)-palhinine B (**1**) (CCDC 2387713).

Identification code	d24954	
Empirical formula	C17 H27 N O4	
Formula weight	309.40	
Temperature	200(2) K	
Wavelength	0.71073 Å	
Crystal system	Orthorhombic	
Space group	P 21 21 21	
Unit cell dimensions	a = 7.9113(4) Å	α= 90°.
	b = 10.7548(5) Å	β= 90°.
	c = 18.7538(8) Å	γ = 90°.
Volume	1595.66(13) $Å^3$	
Z	4	
Density (calculated)	1.288 Mg/m^3	
Absorption coefficient	0.091 mm^{-1}	
F(000)	672	
Crystal size	0.13 x 0.11 x 0.04 mm^3	
Theta range for data collection	2.17 to 25.37°.	
Index ranges	-9<=h<=9, -12<=k<=12, -22<=l<=22	
Reflections collected	31297	
Independent reflections	2930 [R(int) = 0.0674]	
Completeness to theta = 25.37°	99.9 %	
Absorption correction	None	
Max. and min. transmission	0.9964 and 0.9883	
Refinement method	Full-matrix least-squares on F^2	
Data / restraints / parameters	2930 / 0 / 201	
Goodness-of-fit on F^2	1.087	
Final R indices [I>2sigma(I)]	R1 = 0.0435, wR2 = 0.1132	
R indices (all data)	R1 = 0.0483, wR2 = 0.1176	
Absolute structure parameter	0.5(14)	

The sample of (−)-palhinine C (**2**) for X-ray analysis was obtained via slow evaporation in 1/5:EA/Hex. The thermal ellipsoids are shown at the 30% probability level.

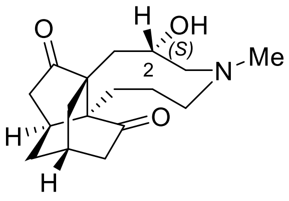

(–)-Palhinine C (**2**)

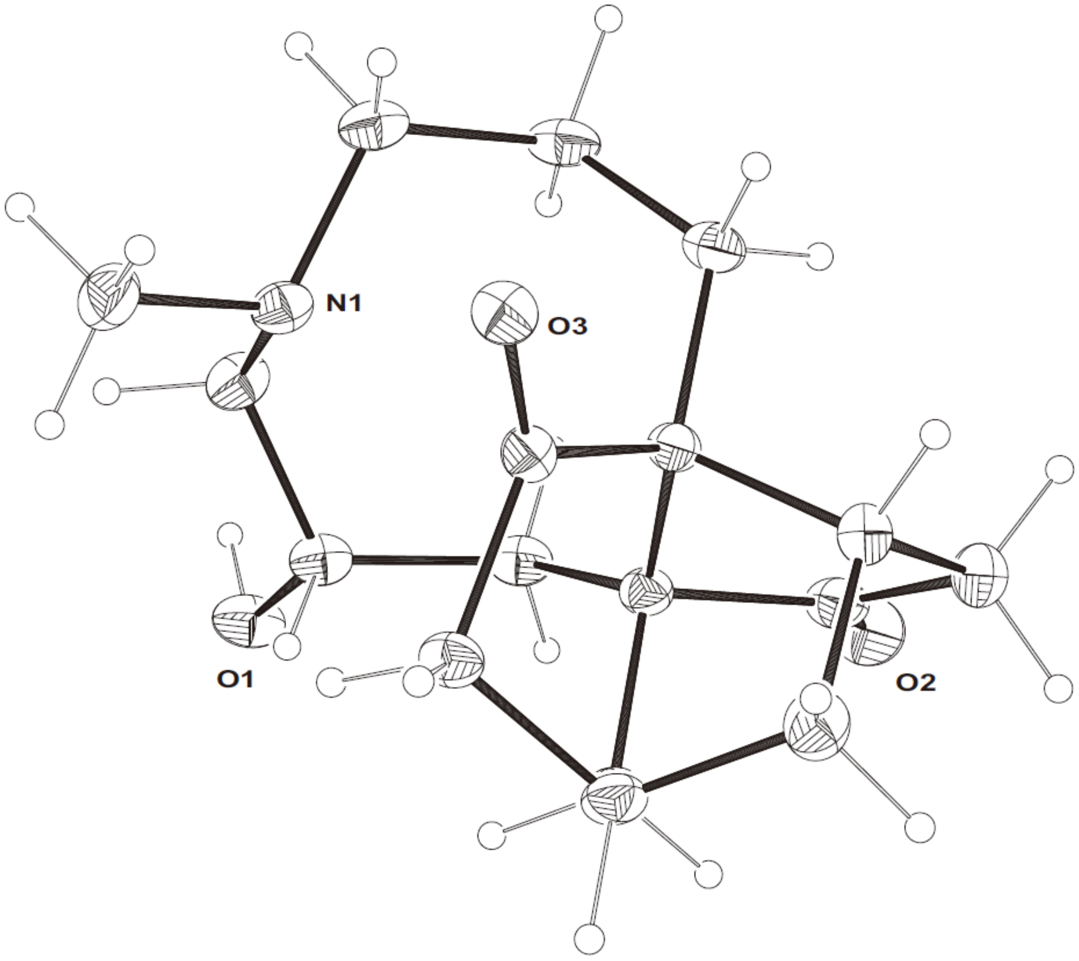

Table SI-6. Crystal data and structure refinement for (−)-palhinine C (**2**) (CCDC 2387714).

Identification code	d25118	
Empirical formula	C17 H25 N O3	
Formula weight	291.38	
Temperature	200(2) K	
Wavelength	0.71073 Å	
Crystal system	Orthorhombic	
Space group	P 21 21 21	
Unit cell dimensions	a = 9.0081(7) Å	α= 90°.
	b = 11.8063(8) Å	β= 90°.
	c = 14.3775(11) Å	γ = 90°.
Volume	1529.1(2) $Å^3$	
Z	4	
Density (calculated)	1.266 Mg/m^3	
Absorption coefficient	0.086 mm^{-1}	
F(000)	632	
Crystal size	0.63 x 0.03 x 0.01 mm^3	
Theta range for data collection	2.23 to 25.05°.	
Index ranges	-7<=h<=10, -14<=k<=14, -17<=l<=17	
Reflections collected	15750	
Independent reflections	2713 [R(int) = 0.0843]	
Completeness to theta = 25.05°	99.9 %	
Absorption correction	None	
Max. and min. transmission	0.9991 and 0.9479	
Refinement method	Full-matrix least-squares on F^2	
Data / restraints / parameters	2713 / 0 / 192	
Goodness-of-fit on F^2	1.055	
Final R indices [I>2sigma(I)]	R1 = 0.0456, wR2 = 0.1022	
R indices (all data)	R1 = 0.0696, wR2 = 0.1187	
Absolute structure parameter	-1.2(19)	

7. ^{1}H- and ^{13}C-NMR Spectra

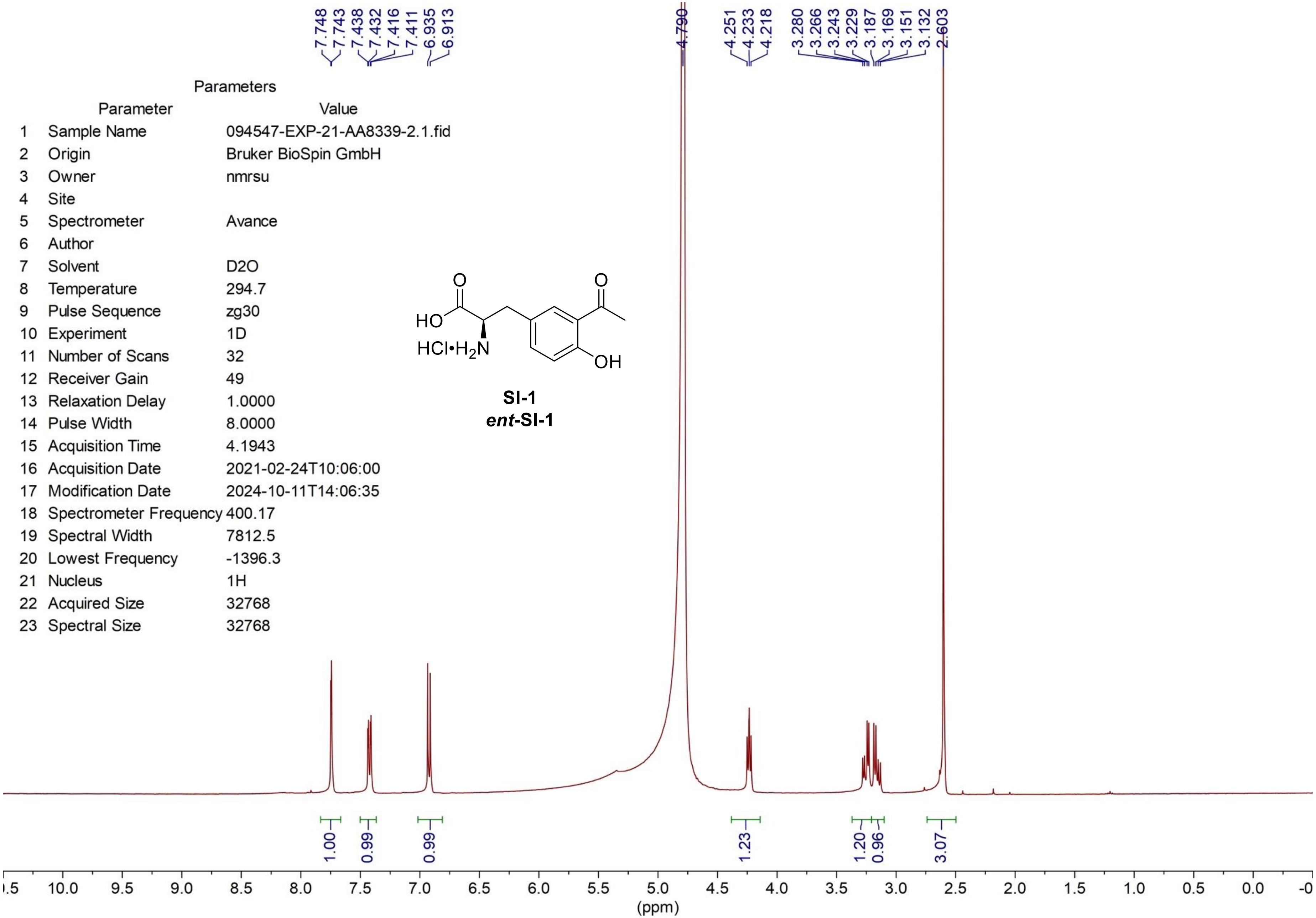
7.748
7.743
7.438
7.432
7.416
7.411
6.935
6.913
4.790
4.251
4.233
4.218
3.280
3.266
3.243
3.229
3.187
3.169
3.151
3.132
2.603
Parameters
Parameter Value
1 Sample Name 094547-EXP-21-AA8339-2.1.fid
2 Origin Bruker BioSpin GmbH
3 Owner nmrsu
4 Site
5 Spectrometer Avance
6 Author
7 Solvent D2O
8 Temperature 294.7
9 Pulse Sequence zg30
10 Experiment 1D
11 Number of Scans 32
12 Receiver Gain 49
13 Relaxation Delay 1.0000
14 Pulse Width 8.0000
15 Acquisition Time 4.1943
16 Acquisition Date 2021-02-24T10:06:00
17 Modification Date 2024-10-11T14:06:35
18 Spectrometer Frequency 400.17
19 Spectral Width 7812.5
20 Lowest Frequency -1396.3
21 Nucleus 1H
22 Acquired Size 32768
23 Spectral Size 32768
O
O
HO
HCl•H2N
OH
SI-1
ent-SI-1
1.00
0.99
0.99
1.23
1.20
0.96
3.07
10.0
9.5
9.0
8.5
8.0
7.5
7.0
6.5
6.0
5.5
5.0
4.5
4.0
3.5
3.0
2.5
2.0
1.5
1.0
0.5
0.0
(ppm)

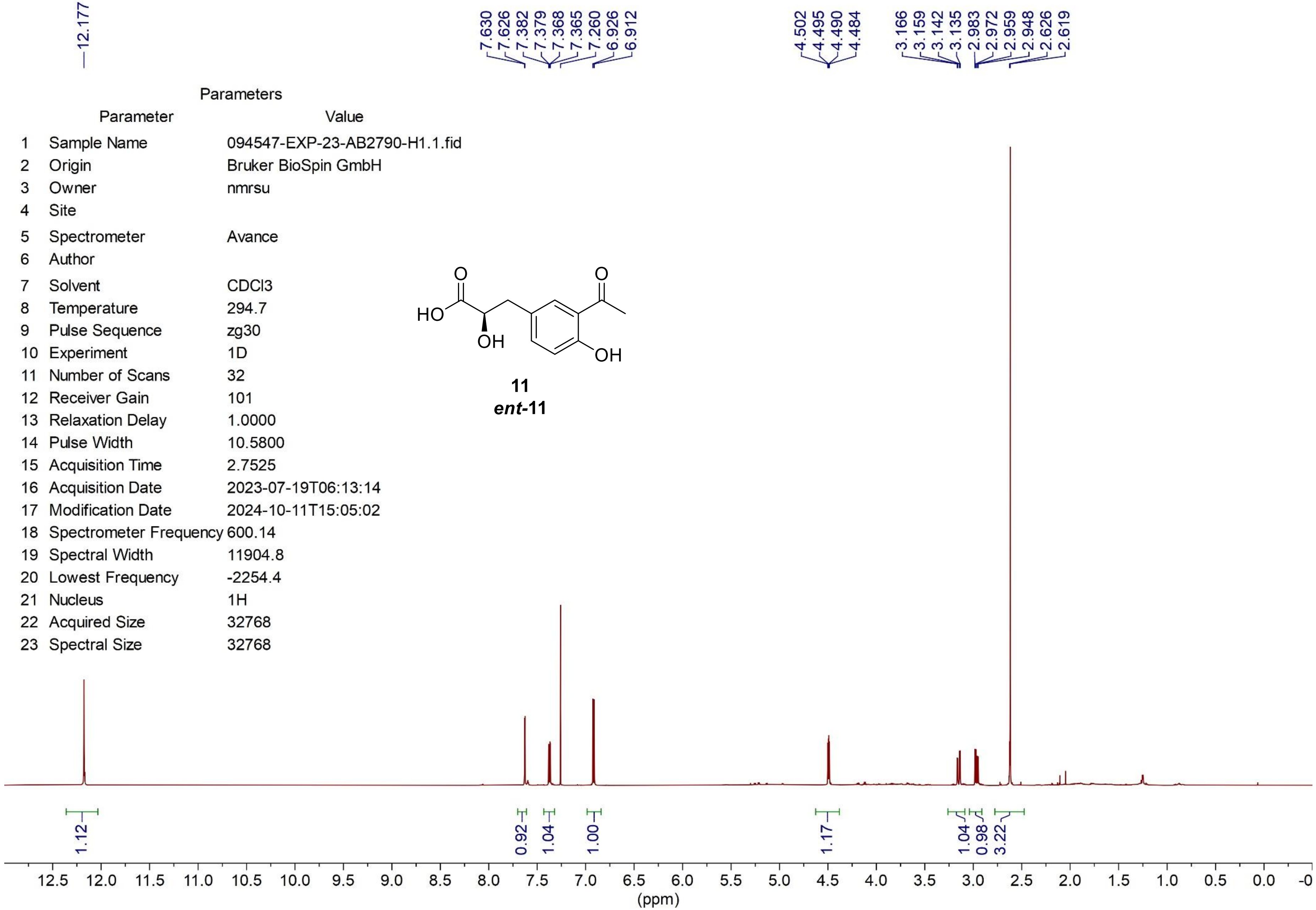
12.177
7.630
7.626
7.382
7.379
7.368
7.365
7.260
6.926
6.912
4.502
4.495
4.490
4.484
3.166
3.159
3.142
3.135
2.983
2.972
2.959
2.948
2.626
2.619
Parameters
Parameter Value
1 Sample Name 094547-EXP-23-AB2790-H1.1.fid
2 Origin Bruker BioSpin GmbH
3 Owner nmrsu
4 Site
5 Spectrometer Avance
6 Author
7 Solvent CDCl3
8 Temperature 294.7
9 Pulse Sequence zg30
10 Experiment 1D
11 Number of Scans 32
12 Receiver Gain 101
13 Relaxation Delay 1.0000
14 Pulse Width 10.5800
15 Acquisition Time 2.7525
16 Acquisition Date 2023-07-19T06:13:14
17 Modification Date 2024-10-11T15:05:02
18 Spectrometer Frequency 600.14
19 Spectral Width 11904.8
20 Lowest Frequency -2254.4
21 Nucleus 1H
22 Acquired Size 32768
23 Spectral Size 32768
HO
O
OH
O
OH
11
ent-11
1.12
0.92
1.04
1.00
1.17
1.04
0.98
3.22
12.5 12.0 11.5 11.0 10.5 10.0 9.5 9.0 8.5 8.0 7.5 7.0 6.5 6.0 5.5 5.0 4.5 4.0 3.5 3.0 2.5 2.0 1.5 1.0 0.5 0.0 -0
(ppm)

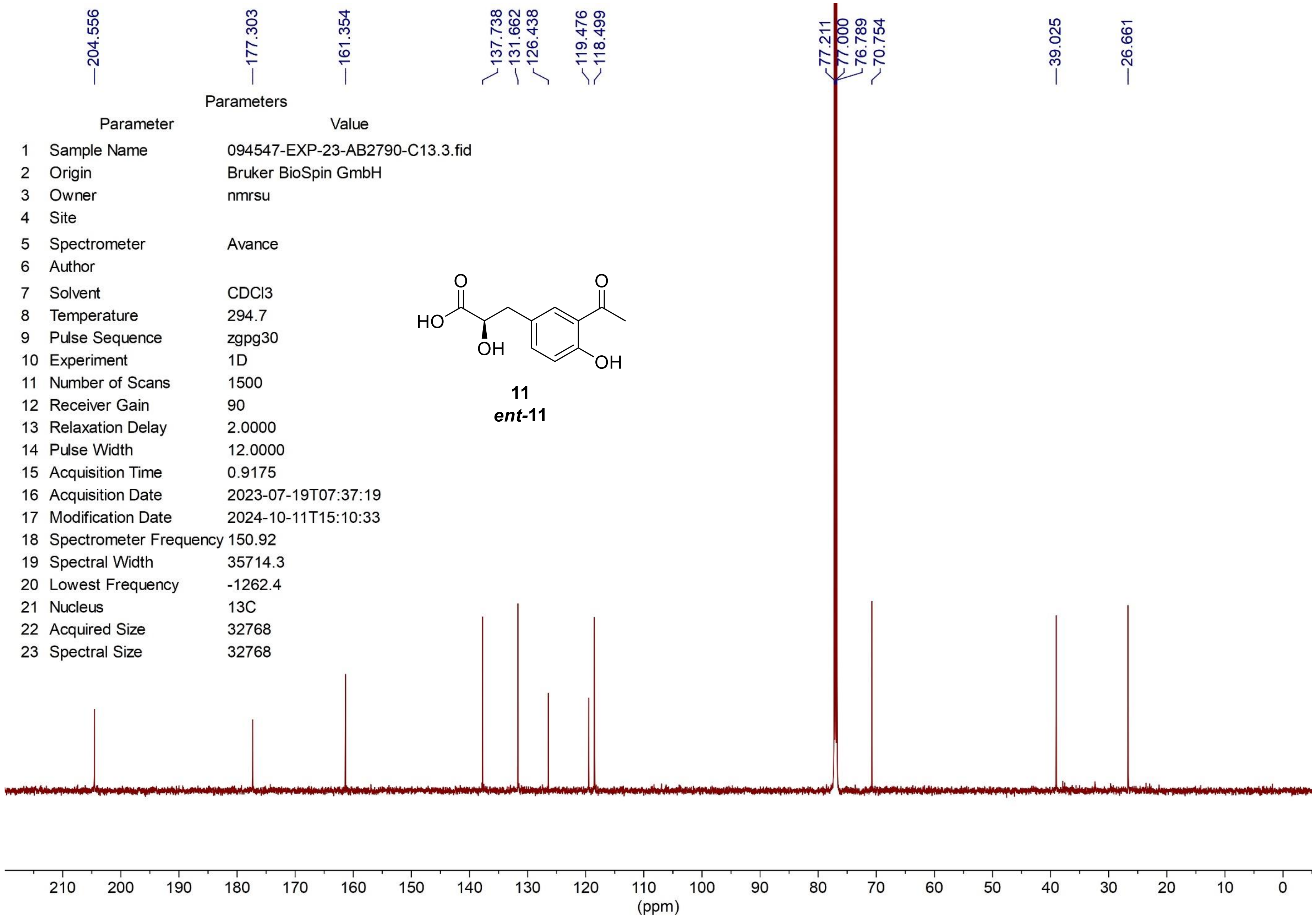
204.556
177.303
161.354
137.738
131.662
126.438
119.476
118.499
77.211
77.000
76.789
70.754
39.025
26.661
Parameters
Parameter Value
1 Sample Name 094547-EXP-23-AB2790-C13.3.fid
2 Origin Bruker BioSpin GmbH
3 Owner nmrsu
4 Site
5 Spectrometer Avance
6 Author
7 Solvent CDCl3
8 Temperature 294.7
9 Pulse Sequence zgpg30
10 Experiment 1D
11 Number of Scans 1500
12 Receiver Gain 90
13 Relaxation Delay 2.0000
14 Pulse Width 12.0000
15 Acquisition Time 0.9175
16 Acquisition Date 2023-07-19T07:37:19
17 Modification Date 2024-10-11T15:10:33
18 Spectrometer Frequency 150.92
19 Spectral Width 35714.3
20 Lowest Frequency -1262.4
21 Nucleus 13C
22 Acquired Size 32768
23 Spectral Size 32768
HO
O
OH
O
OH
11
ent-11
210 200 190 180 170 160 150 140 130 120 110 100 90 80 70 60 50 40 30 20 10 0
(ppm)

Parameters

	Parameter	Value
1	Sample Name	hYpfNUK6Qq2PXIOS8ixDdw.2.fid
2	Origin	Bruker BioSpin GmbH
3	Owner	nmrsu
4	Site	
5	Spectrometer	Avance
6	Author	
7	Solvent	CDCl3
8	Temperature	294.8
9	Pulse Sequence	zg30
10	Experiment	1D
11	Number of Scans	16
12	Receiver Gain	101
13	Relaxation Delay	1.0000
14	Pulse Width	8.0000
15	Acquisition Time	4.1943
16	Acquisition Date	2021-03-22T17:06:46
17	Modification Date	2021-03-22T17:20:18
18	Spectrometer Frequency	400.17
19	Spectral Width	7812.5
20	Lowest Frequency	-1445.4
21	Nucleus	1H
22	Acquired Size	32768
23	Spectral Size	65536

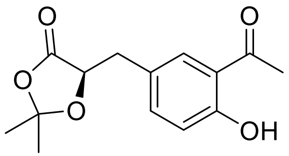

SI-2

***ent*-SI-2**

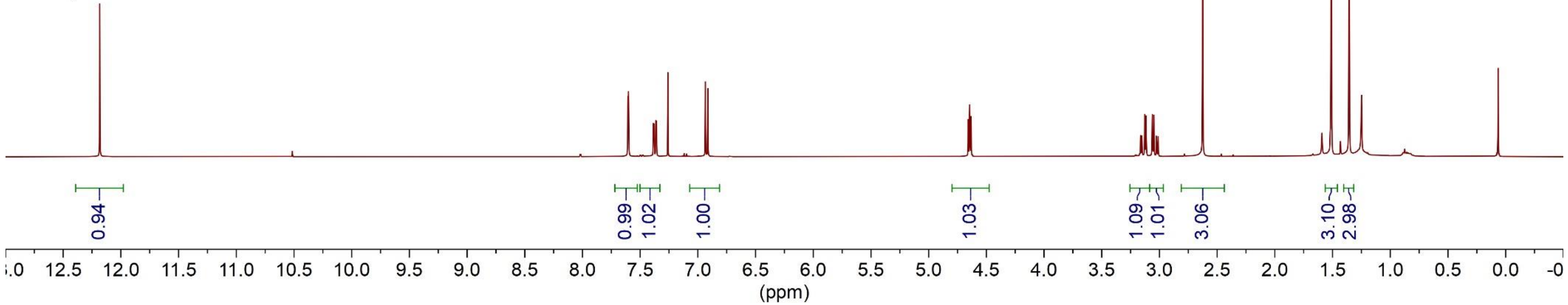

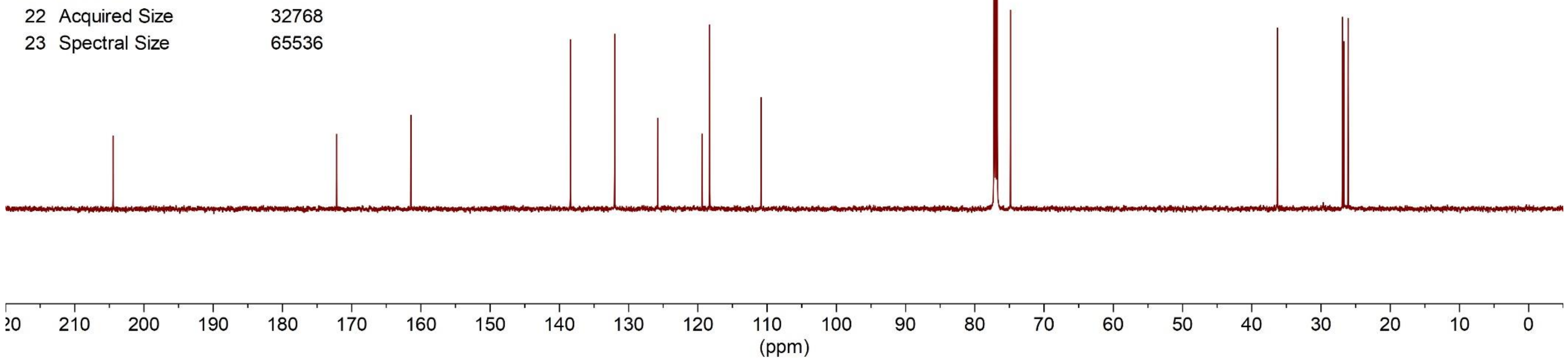

Parameters

	Parameter	Value
1	Sample Name	094547-exp-23-ab41-s3-c13-2.3.fid
2	Origin	Bruker BioSpin GmbH
3	Owner	nmrsu
4	Site	
5	Spectrometer	Avance
6	Author	
7	Solvent	CDCl3
8	Temperature	294.7
9	Pulse Sequence	zgpg30
10	Experiment	1D
11	Number of Scans	1200
12	Receiver Gain	90
13	Relaxation Delay	2.0000
14	Pulse Width	12.0000
15	Acquisition Time	0.9175
16	Acquisition Date	2023-07-18T02:45:46
17	Modification Date	2024-10-10T03:42:01
18	Spectrometer Frequency	150.92
19	Spectral Width	35714.3
20	Lowest Frequency	-1262.7
21	Nucleus	13C
22	Acquired Size	32768
23	Spectral Size	65536

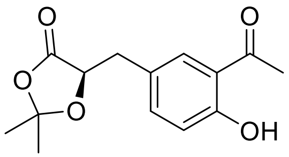

SI-2
***ent*-SI-2**

Parameters

	Parameter	Value
1	Sample Name	fBEmb3VTQba6CzqpimfVbg.1.fid
2	Origin	Bruker BioSpin GmbH
3	Owner	nmrsu
4	Site	
5	Spectrometer	Avance
6	Author	
7	Solvent	CDCl3
8	Temperature	295.3
9	Pulse Sequence	zg30
10	Experiment	1D
11	Number of Scans	32
12	Receiver Gain	101
13	Relaxation Delay	1.0000
14	Pulse Width	8.0000
15	Acquisition Time	4.1943
16	Acquisition Date	2021-05-17T18:38:45
17	Modification Date	2021-05-17T18:47:11
18	Spectrometer Frequency	400.17
19	Spectral Width	7812.5
20	Lowest Frequency	-1445.4
21	Nucleus	1H
22	Acquired Size	32768
23	Spectral Size	65536

12
***ent*-12**

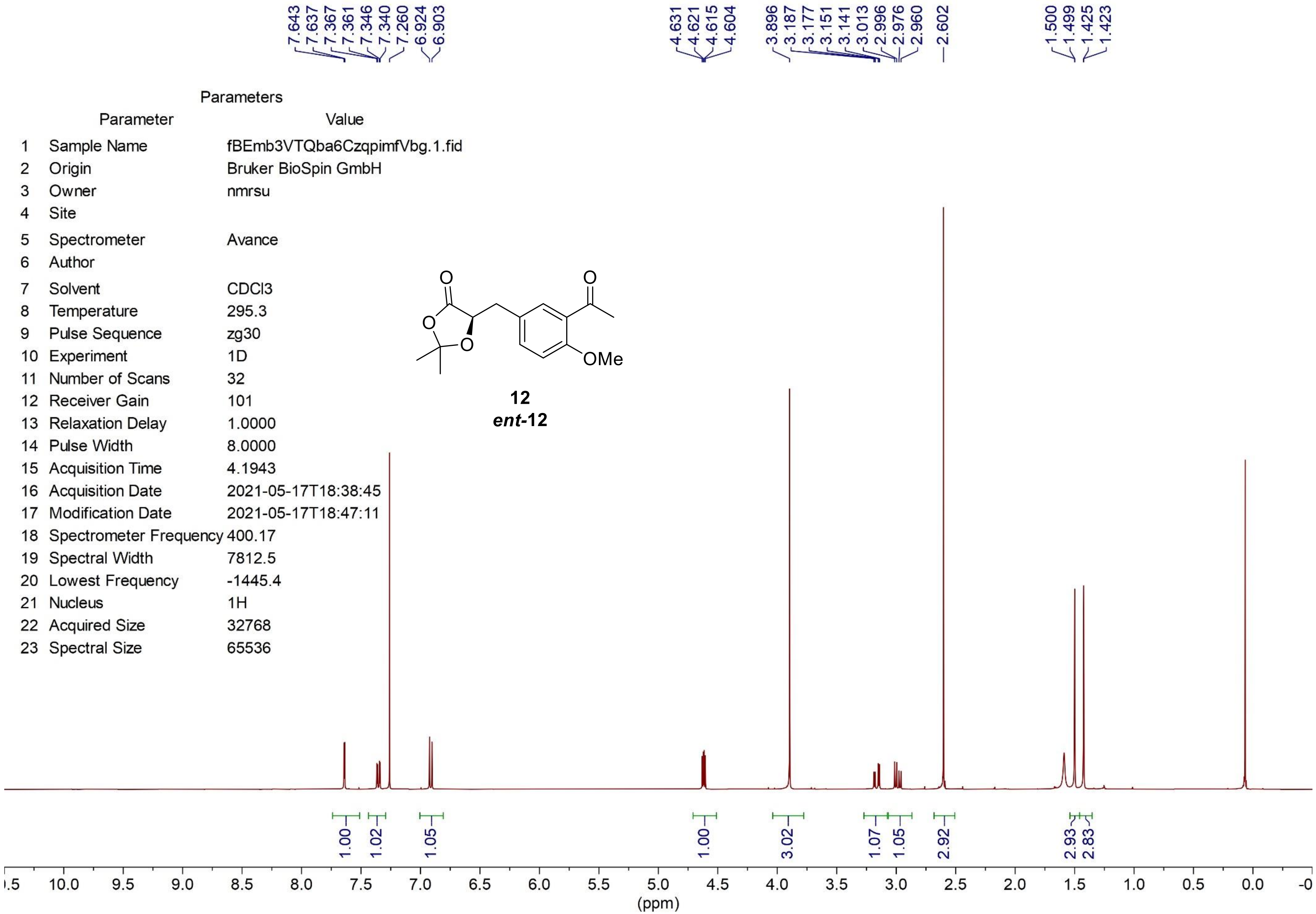

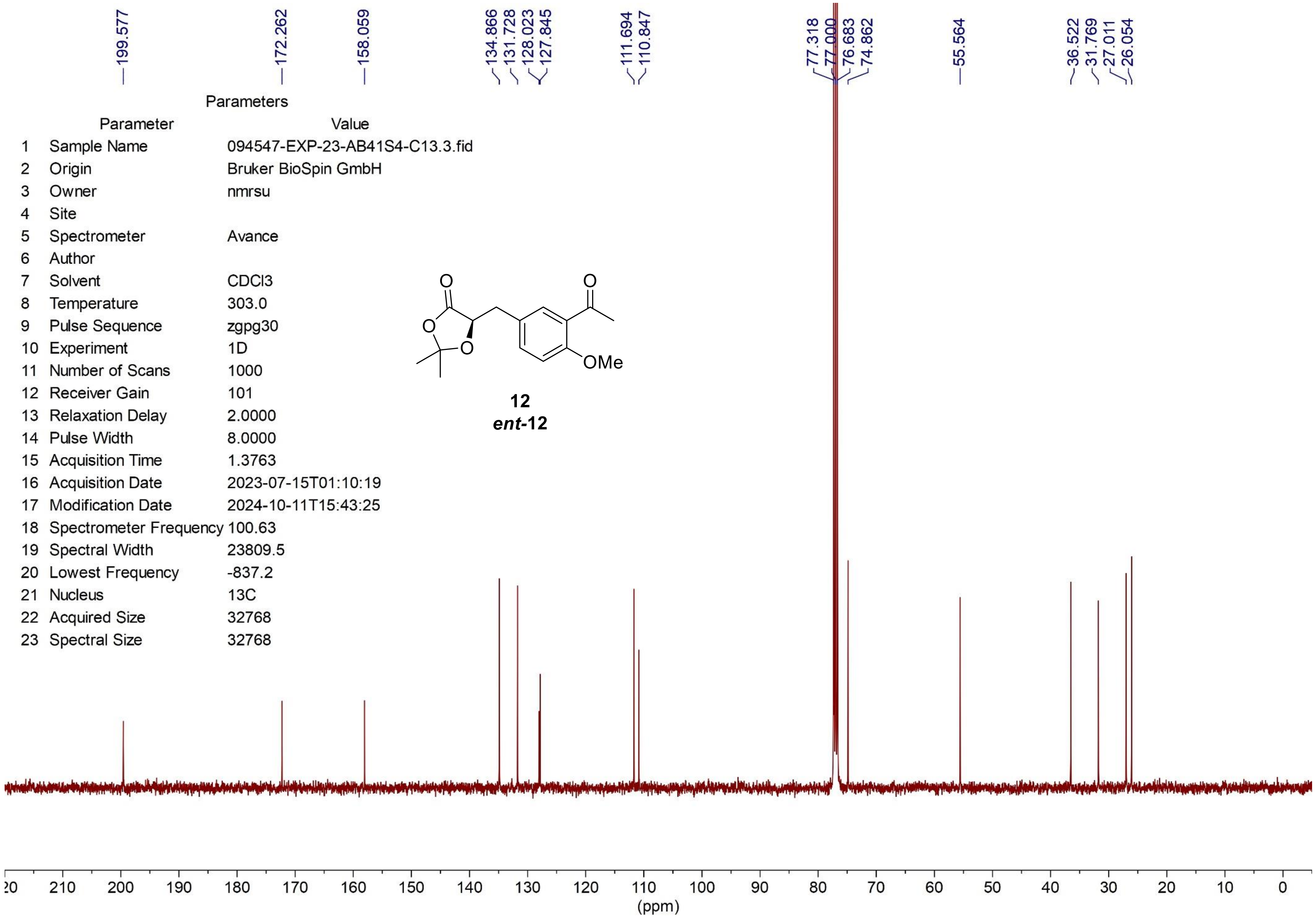

Parameters

	Parameter	Value
1	Sample Name	094547-EXP-23-AB41S4-C13.3.fid
2	Origin	Bruker BioSpin GmbH
3	Owner	nmrsu
4	Site	
5	Spectrometer	Avance
6	Author	
7	Solvent	CDCl3
8	Temperature	303.0
9	Pulse Sequence	zgpg30
10	Experiment	1D
11	Number of Scans	1000
12	Receiver Gain	101
13	Relaxation Delay	2.0000
14	Pulse Width	8.0000
15	Acquisition Time	1.3763
16	Acquisition Date	2023-07-15T01:10:19
17	Modification Date	2024-10-11T15:43:25
18	Spectrometer Frequency	100.63
19	Spectral Width	23809.5
20	Lowest Frequency	-837.2
21	Nucleus	13C
22	Acquired Size	32768
23	Spectral Size	32768

12
***ent*-12**

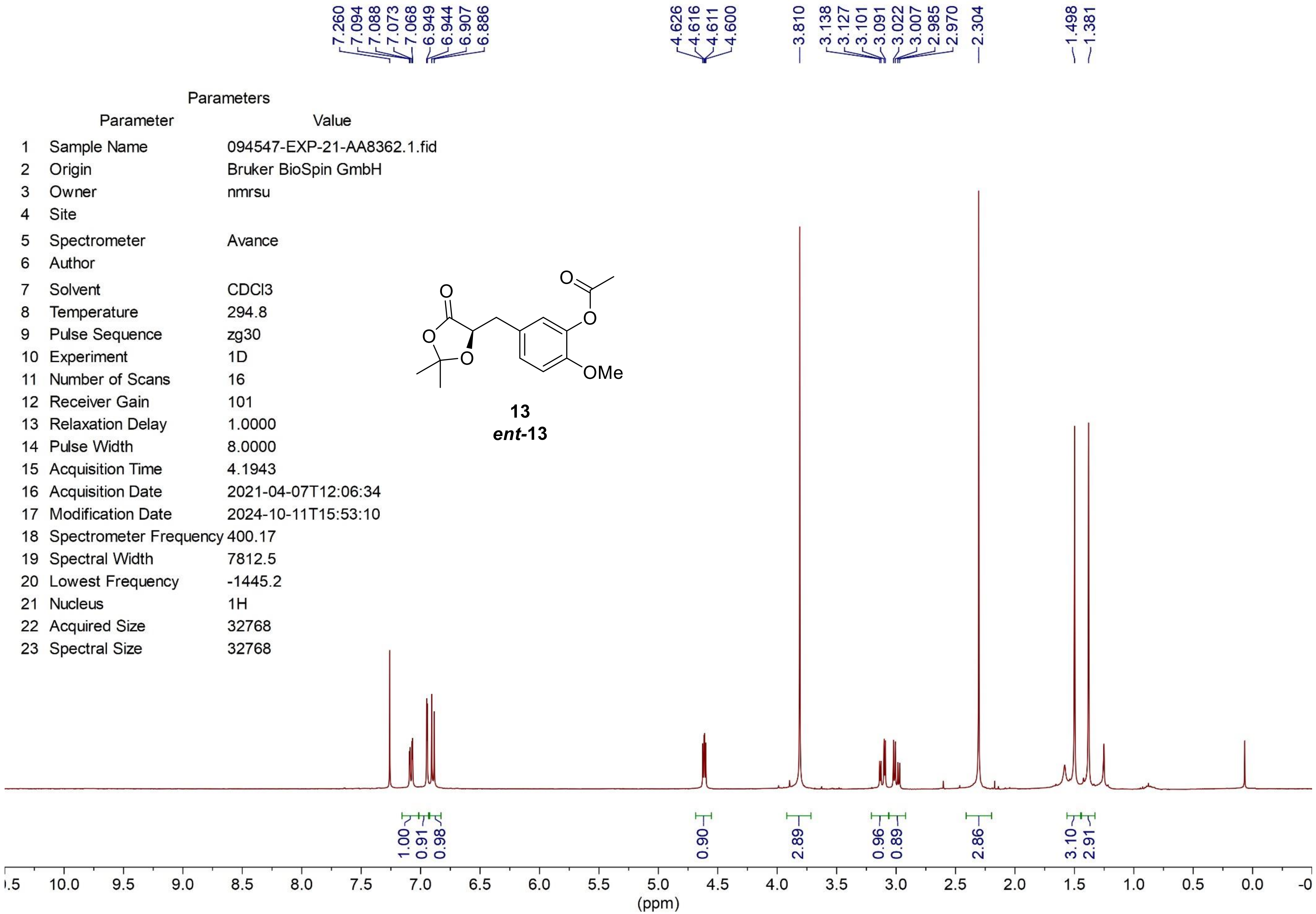

13

***ent*-13**

Parameters

	Parameter	Value
1	Sample Name	094547-EXP-21-AA8362.1.fid
2	Origin	Bruker BioSpin GmbH
3	Owner	nmrsu
4	Site	
5	Spectrometer	Avance
6	Author	
7	Solvent	CDCl3
8	Temperature	294.8
9	Pulse Sequence	zg30
10	Experiment	1D
11	Number of Scans	16
12	Receiver Gain	101
13	Relaxation Delay	1.0000
14	Pulse Width	8.0000
15	Acquisition Time	4.1943
16	Acquisition Date	2021-04-07T12:06:34
17	Modification Date	2024-10-11T15:53:10
18	Spectrometer Frequency	400.17
19	Spectral Width	7812.5
20	Lowest Frequency	-1445.2
21	Nucleus	1H
22	Acquired Size	32768
23	Spectral Size	32768

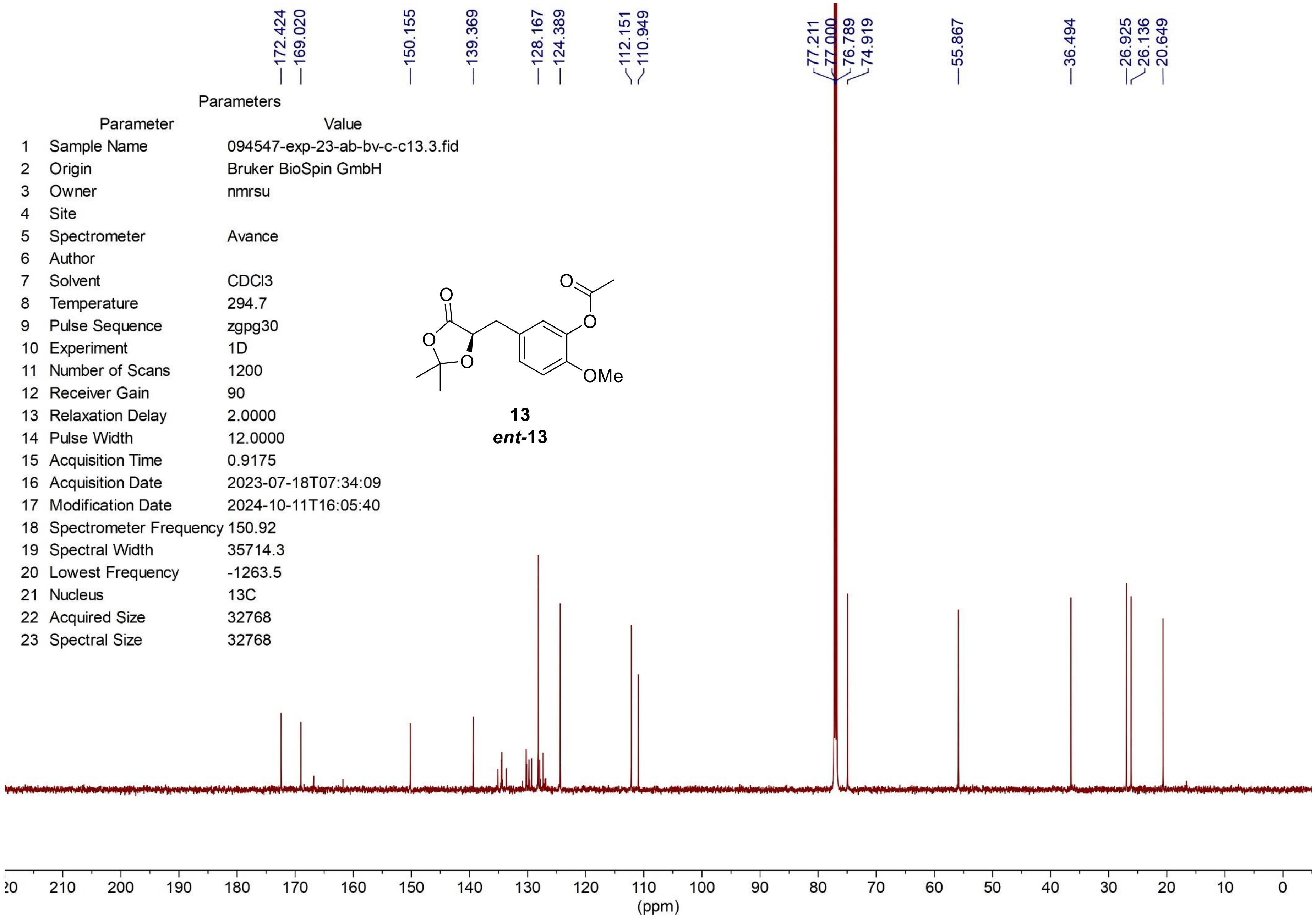
172.424
169.020
150.155
139.369
128.167
124.389
112.151
110.949
77.211
77.000
76.789
74.919
55.867
36.494
26.925
26.136
20.649
Parameters
Parameter
Value
1 Sample Name 094547-exp-23-ab-bv-c-c13.3.fid
2 Origin Bruker BioSpin GmbH
3 Owner nmrsu
4 Site
5 Spectrometer Avance
6 Author
7 Solvent CDCl3
8 Temperature 294.7
9 Pulse Sequence zgpg30
10 Experiment 1D
11 Number of Scans 1200
12 Receiver Gain 90
13 Relaxation Delay 2.0000
14 Pulse Width 12.0000
15 Acquisition Time 0.9175
16 Acquisition Date 2023-07-18T07:34:09
17 Modification Date 2024-10-11T16:05:40
18 Spectrometer Frequency 150.92
19 Spectral Width 35714.3
20 Lowest Frequency -1263.5
21 Nucleus 13C
22 Acquired Size 32768
23 Spectral Size 32768
O
O
O
O
O
O
OMe
13
ent-13
(ppm)

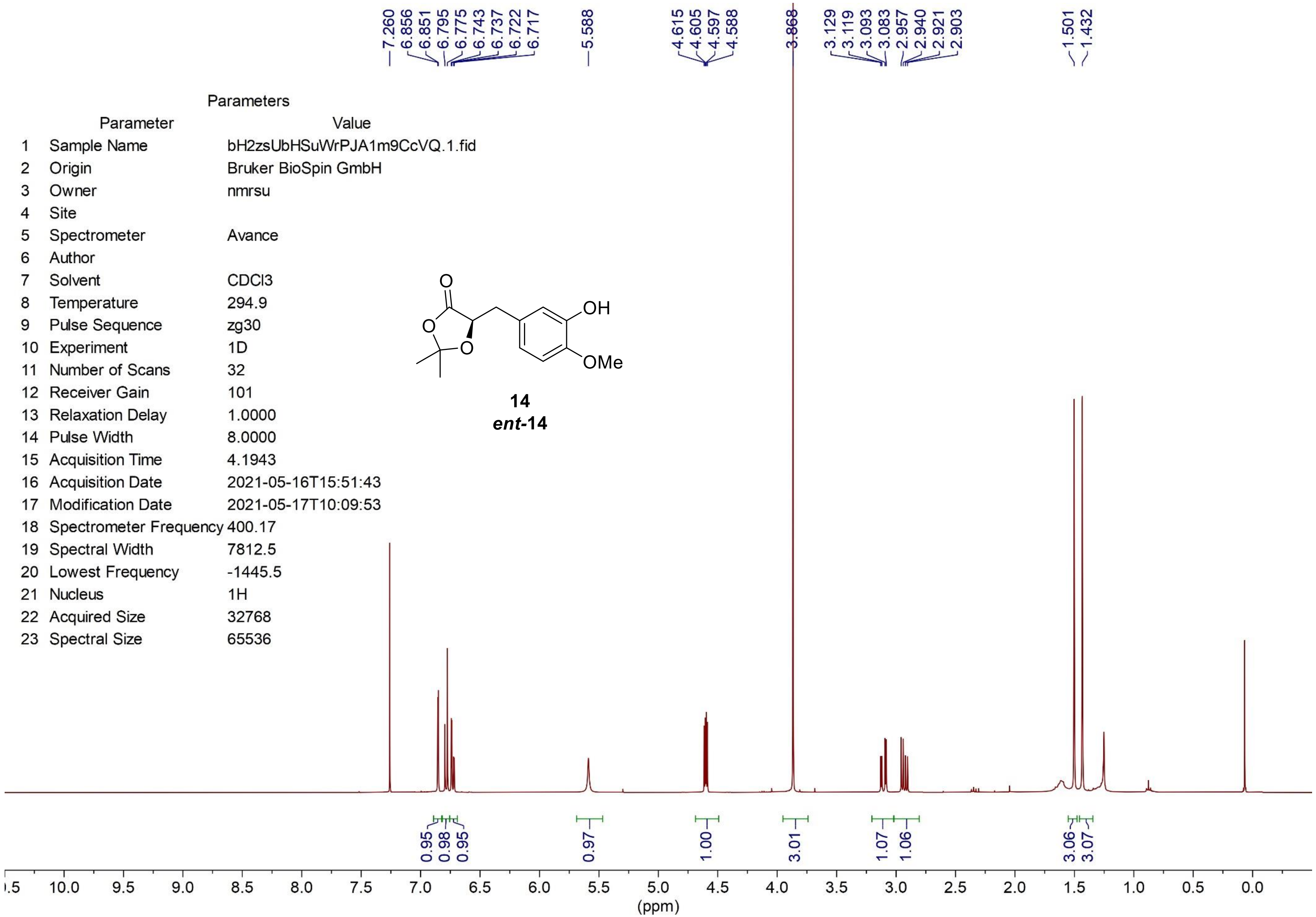
7.260
6.856
6.851
6.795
6.775
6.743
6.737
6.722
6.717
5.588
4.615
4.605
4.597
4.588
3.868
3.129
3.119
3.093
3.083
2.957
2.940
2.921
2.903
1.501
1.432
Parameters
Parameter Value
1 Sample Name bH2zsUbHSuWrPJA1m9CcVQ.1.fid
2 Origin Bruker BioSpin GmbH
3 Owner nmrsu
4 Site
5 Spectrometer Avance
6 Author
7 Solvent CDCl3
8 Temperature 294.9
9 Pulse Sequence zg30
10 Experiment 1D
11 Number of Scans 32
12 Receiver Gain 101
13 Relaxation Delay 1.0000
14 Pulse Width 8.0000
15 Acquisition Time 4.1943
16 Acquisition Date 2021-05-16T15:51:43
17 Modification Date 2021-05-17T10:09:53
18 Spectrometer Frequency 400.17
19 Spectral Width 7812.5
20 Lowest Frequency -1445.5
21 Nucleus 1H
22 Acquired Size 32768
23 Spectral Size 65536
O
OH
O
O
OMe
14
ent-14
0.95
0.98
0.95
0.97
1.00
3.01
1.07
1.06
3.06
3.07
10.0 9.5 9.0 8.5 8.0 7.5 7.0 6.5 6.0 5.5 5.0 4.5 4.0 3.5 3.0 2.5 2.0 1.5 1.0 0.5 0.0
(ppm)

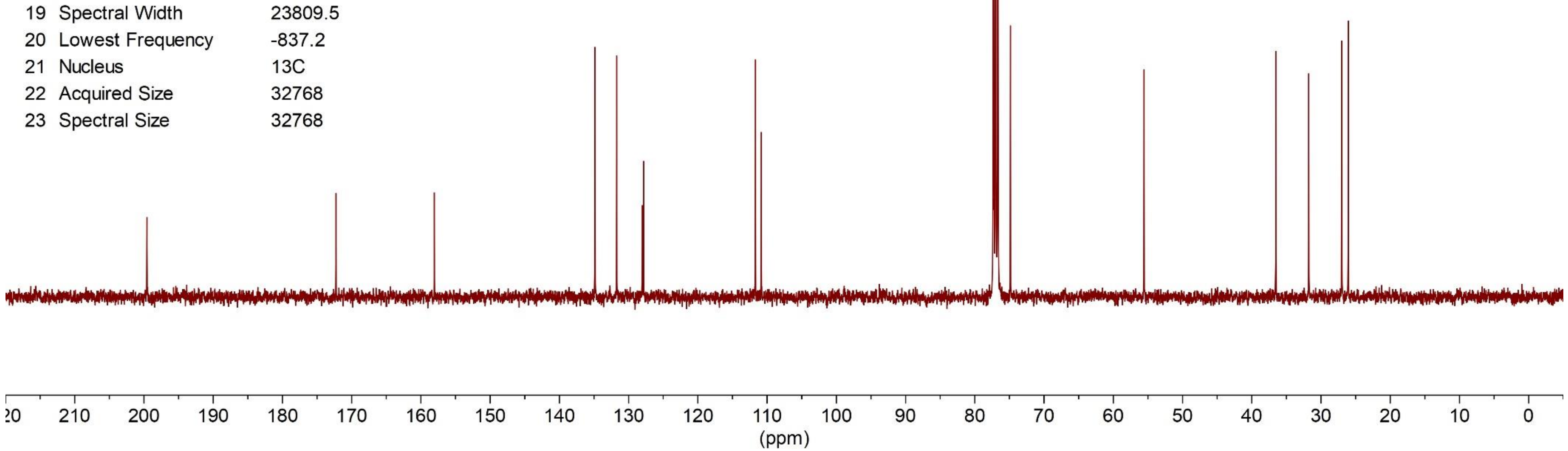

Parameters

	Parameter	Value
1	Sample Name	094547-EXP-23-AB41S4-C13.3.fid
2	Origin	Bruker BioSpin GmbH
3	Owner	nmrsu
4	Site	
5	Spectrometer	Avance
6	Author	
7	Solvent	CDCl3
8	Temperature	303.0
9	Pulse Sequence	zgpg30
10	Experiment	1D
11	Number of Scans	1000
12	Receiver Gain	101
13	Relaxation Delay	2.0000
14	Pulse Width	8.0000
15	Acquisition Time	1.3763
16	Acquisition Date	2023-07-15T01:10:19
17	Modification Date	2024-10-11T15:43:25
18	Spectrometer Frequency	100.63
19	Spectral Width	23809.5
20	Lowest Frequency	-837.2
21	Nucleus	13C
22	Acquired Size	32768
23	Spectral Size	32768

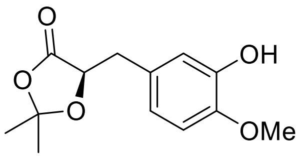

14
***ent*-14**

Parameters

	Parameter	Value
1	Sample Name	mt6lm5VWR0eJTrAQN2nbmA.1.fid
2	Origin	Bruker BioSpin GmbH
3	Owner	nmrsu
4	Site	
5	Spectrometer	Avance
6	Author	
7	Solvent	CDCl3
8	Temperature	294.7
9	Pulse Sequence	zg30
10	Experiment	1D
11	Number of Scans	16
12	Receiver Gain	101
13	Relaxation Delay	1.0000
14	Pulse Width	8.0000
15	Acquisition Time	4.1943
16	Acquisition Date	2021-04-24T21:38:49
17	Modification Date	2021-05-17T01:30:33
18	Spectrometer Frequency	400.17
19	Spectral Width	7812.5
20	Lowest Frequency	-1445.3
21	Nucleus	1H
22	Acquired Size	32768
23	Spectral Size	65536

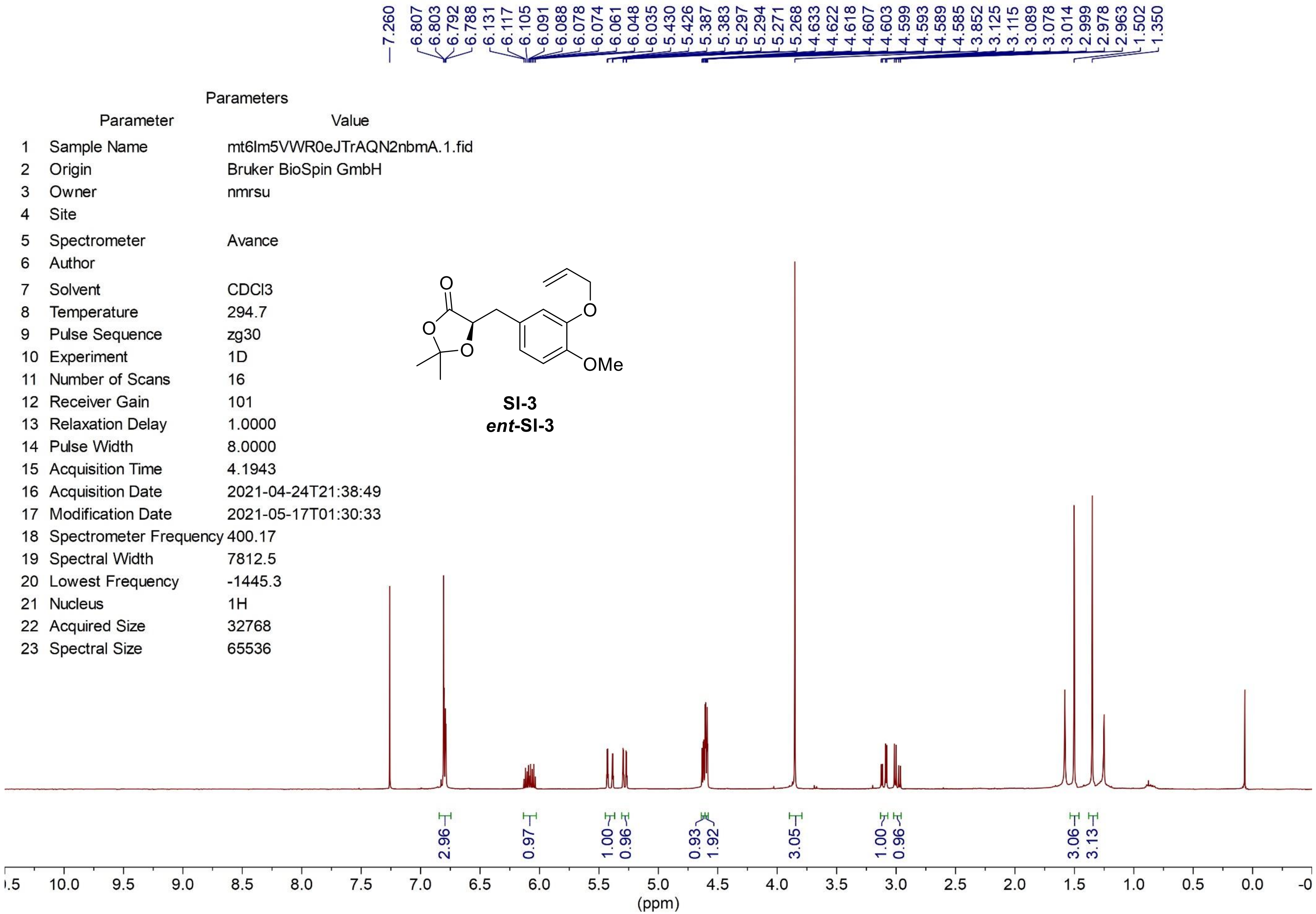

SI-3
***ent*-SI-3**

Parameters

	Parameter	Value
1	Sample Name	094547-EXP-22-AB27ALLYL-C13.1.fid
2	Origin	Bruker BioSpin GmbH
3	Owner	nmrsu
4	Site	
5	Spectrometer	Avance
6	Author	
7	Solvent	CDCl3
8	Temperature	294.7
9	Pulse Sequence	zgpg30
10	Experiment	1D
11	Number of Scans	1500
12	Receiver Gain	90
13	Relaxation Delay	2.0000
14	Pulse Width	11.5000
15	Acquisition Time	0.9175
16	Acquisition Date	2022-07-09T17:45:11
17	Modification Date	2024-10-09T14:18:26
18	Spectrometer Frequency	150.92
19	Spectral Width	35714.3
20	Lowest Frequency	-1263.8
21	Nucleus	13C
22	Acquired Size	32768
23	Spectral Size	65536

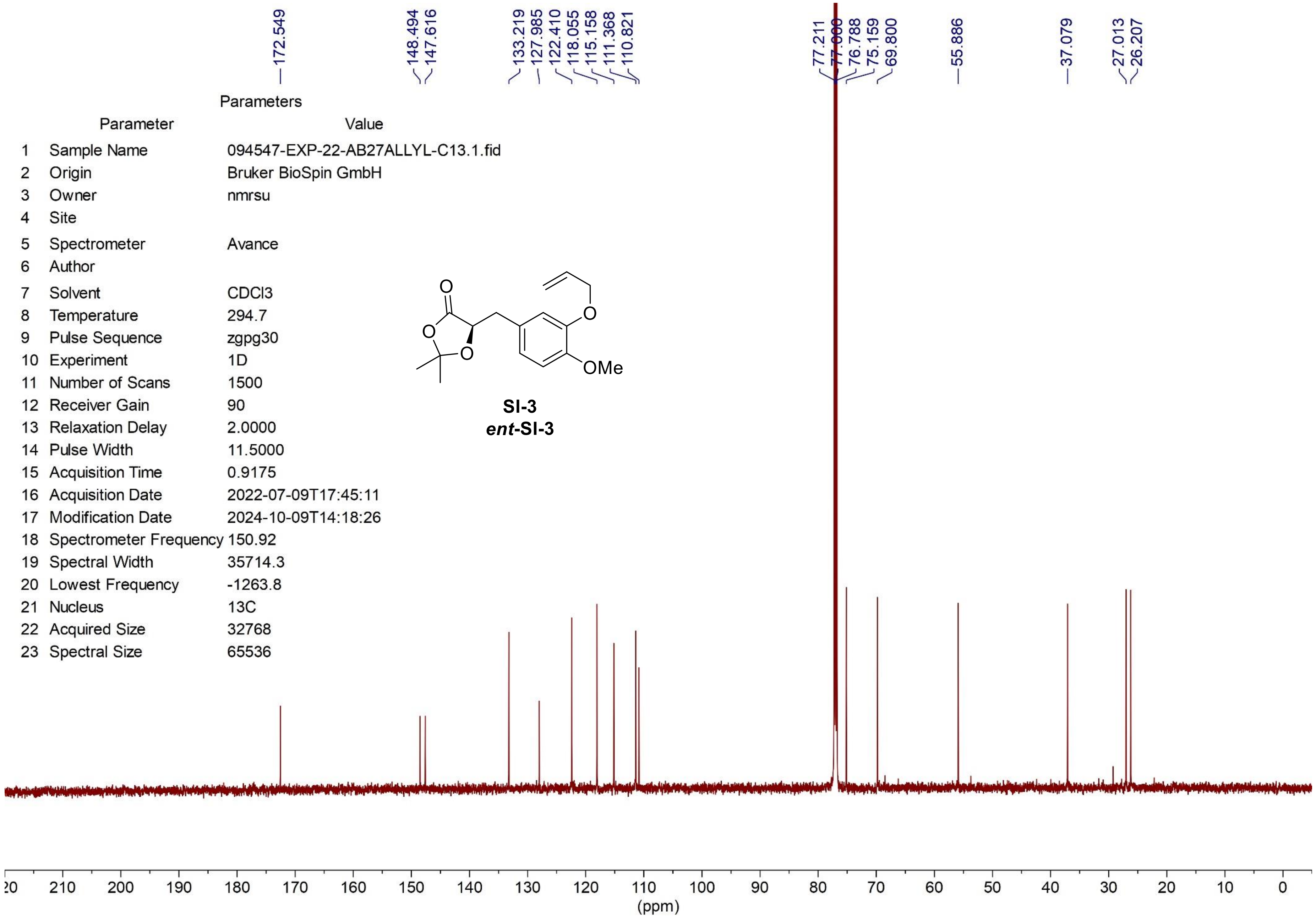

Parameters

	Parameter	Value
1	Sample Name	$6UfstkVRhSIj58LUIJdTA.1.fid
2	Origin	Bruker BioSpin GmbH
3	Owner	nmrsu
4	Site	
5	Spectrometer	Avance
6	Author	
7	Solvent	CDCl3
8	Temperature	294.8
9	Pulse Sequence	zg30
10	Experiment	1D
11	Number of Scans	16
12	Receiver Gain	101
13	Relaxation Delay	1.0000
14	Pulse Width	8.0000
15	Acquisition Time	4.1943
16	Acquisition Date	2021-05-14T16:09:25
17	Modification Date	2021-05-17T01:37:57
18	Spectrometer Frequency	400.17
19	Spectral Width	7812.5
20	Lowest Frequency	-1445.7
21	Nucleus	1H
22	Acquired Size	32768
23	Spectral Size	65536

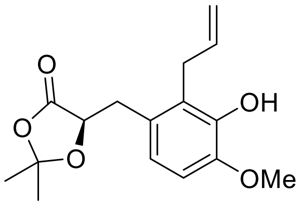

15
***ent*-15**

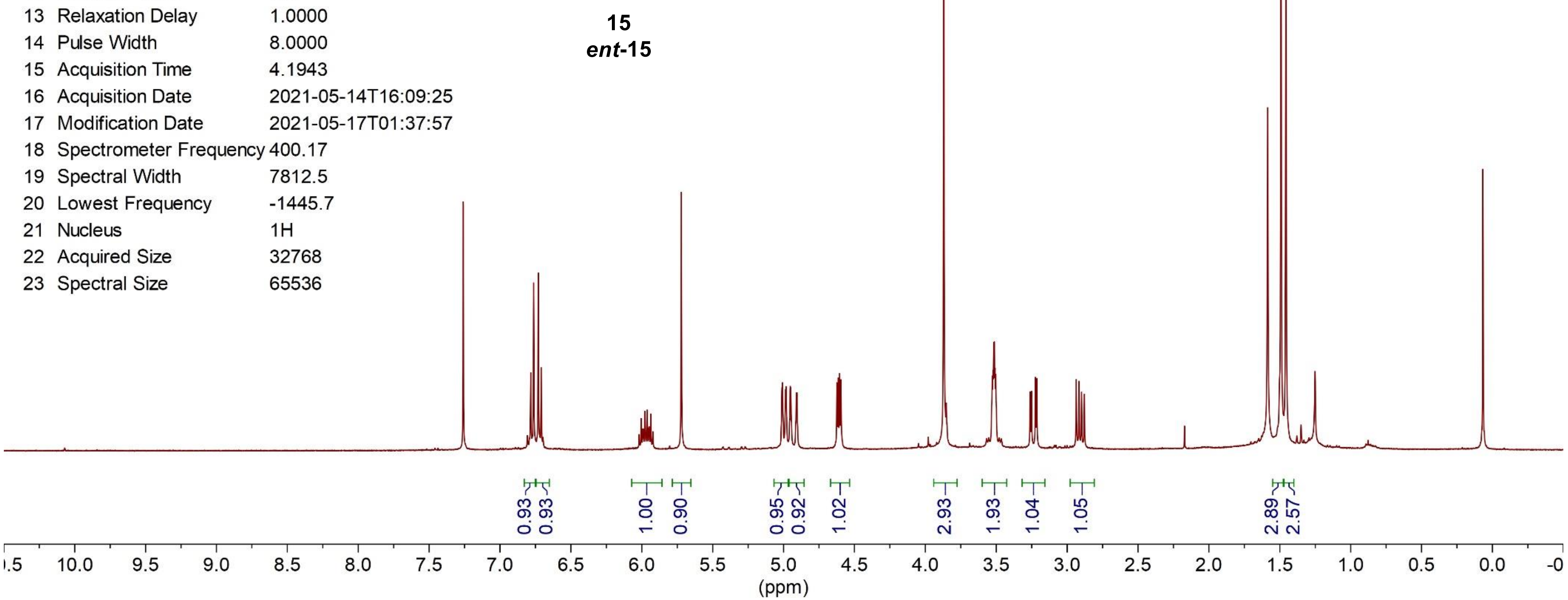

	Parameter	Value
1	Sample Name	094547-exp-23-ab4100-c13.7.fid
2	Origin	Bruker BioSpin GmbH
3	Owner	nmrsu
4	Site	
5	Spectrometer	Avance
6	Author	
7	Solvent	CDCl3
8	Temperature	294.7
9	Pulse Sequence	zgpg30
10	Experiment	1D
11	Number of Scans	1200
12	Receiver Gain	90
13	Relaxation Delay	2.0000
14	Pulse Width	12.0000
15	Acquisition Time	0.9175
16	Acquisition Date	2023-07-18T05:09:23
17	Modification Date	2024-10-09T14:42:47
18	Spectrometer Frequency	150.92
19	Spectral Width	35714.3
20	Lowest Frequency	-1264.1
21	Nucleus	13C
22	Acquired Size	32768
23	Spectral Size	65536

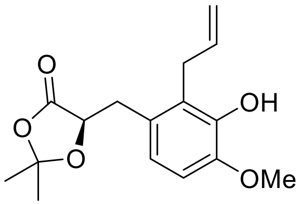

15
***ent*-15**

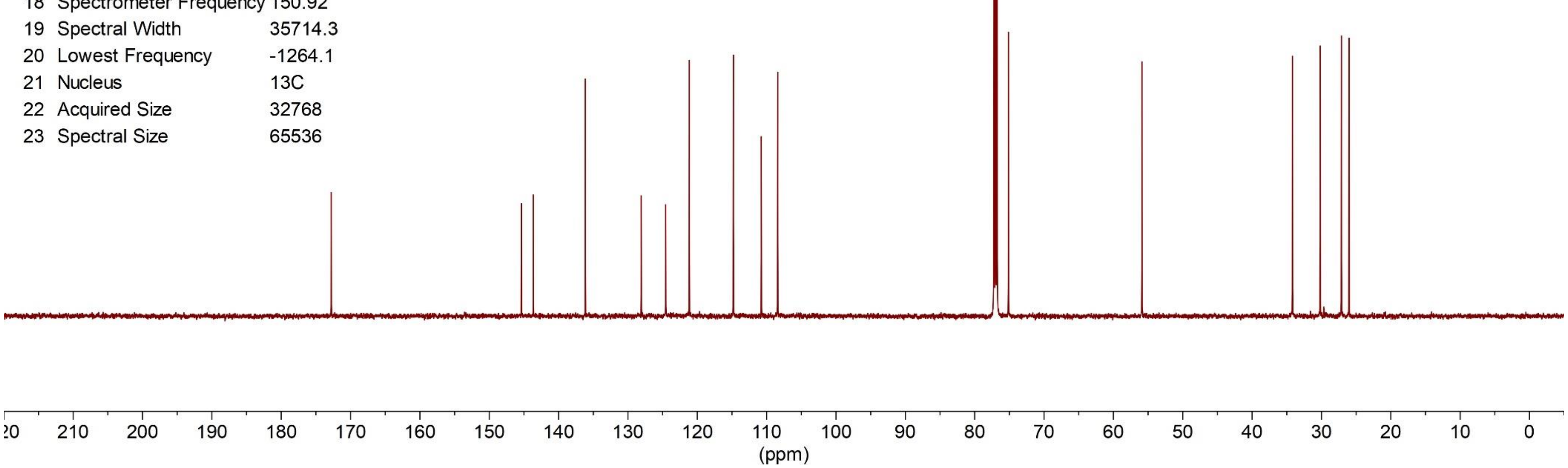

Parameters

	Parameter	Value
1	Sample Name	094547-EXP-21-AA9844.1.fid
2	Origin	Bruker BioSpin GmbH
3	Owner	nmrsu
4	Site	
5	Spectrometer	Avance
6	Author	
7	Solvent	CDCl3
8	Temperature	294.7
9	Pulse Sequence	zg30
10	Experiment	1D
11	Number of Scans	64
12	Receiver Gain	101
13	Relaxation Delay	1.0000
14	Pulse Width	7.5400
15	Acquisition Time	4.1943
16	Acquisition Date	2021-12-19T22:01:30
17	Modification Date	2021-12-22T11:27:31
18	Spectrometer Frequency	400.17
19	Spectral Width	7812.5
20	Lowest Frequency	-1444.7
21	Nucleus	1H
22	Acquired Size	32768
23	Spectral Size	65536

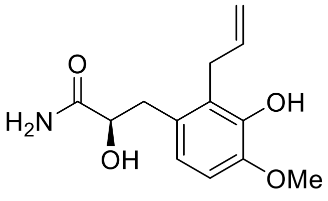

16
***ent*-16**

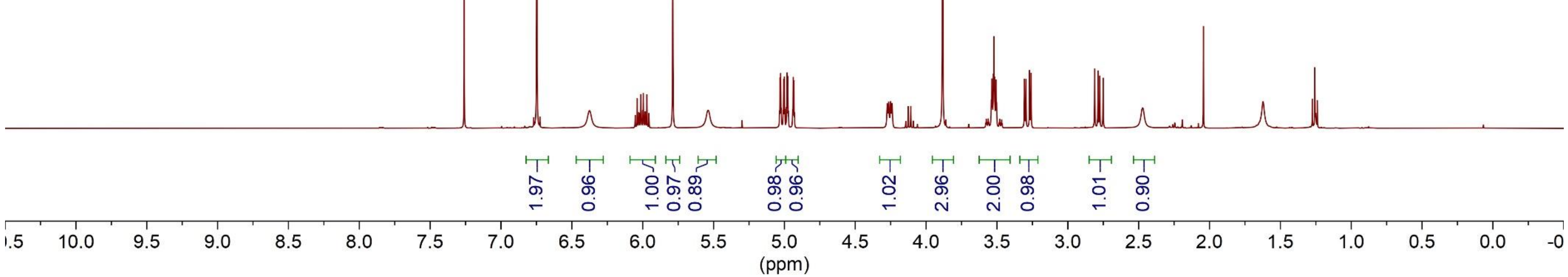

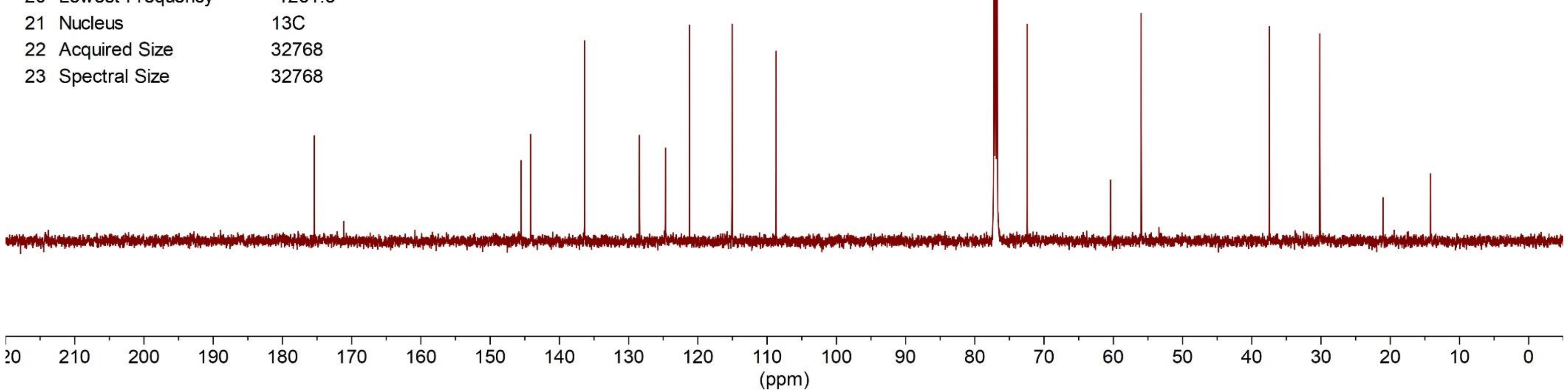

Parameters

	Parameter	Value
1	Sample Name	R40kvrZkQ0GEfNycRtBHiw.1.fid
2	Origin	Bruker BioSpin GmbH
3	Owner	nmrsu
4	Site	
5	Spectrometer	Avance
6	Author	
7	Solvent	CDCl3
8	Temperature	298.2
9	Pulse Sequence	zgpg30
10	Experiment	1D
11	Number of Scans	1200
12	Receiver Gain	101
13	Relaxation Delay	2.0000
14	Pulse Width	12.0000
15	Acquisition Time	0.9175
16	Acquisition Date	2024-02-21T11:38:59
17	Modification Date	2024-10-11T17:21:16
18	Spectrometer Frequency	150.92
19	Spectral Width	35714.3
20	Lowest Frequency	-1261.3
21	Nucleus	13C
22	Acquired Size	32768
23	Spectral Size	32768

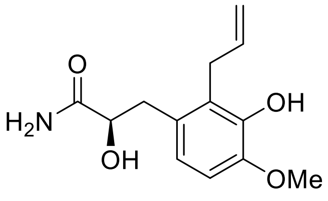

16
***ent*-16**

Parameters

	Parameter	Value
1	Sample Name	hZQMI3UsSYqxqLgNEpTmHQ.1.fid
2	Origin	Bruker BioSpin GmbH
3	Owner	nmrsu
4	Site	
5	Spectrometer	Avance
6	Author	
7	Solvent	MeOD
8	Temperature	298.0
9	Pulse Sequence	zg30
10	Experiment	1D
11	Number of Scans	16
12	Receiver Gain	90
13	Relaxation Delay	1.0000
14	Pulse Width	8.0000
15	Acquisition Time	4.1943
16	Acquisition Date	2023-11-28T01:02:44
17	Modification Date	2024-10-14T09:59:09
18	Spectrometer Frequency	400.17
19	Spectral Width	7812.5
20	Lowest Frequency	-1442.5
21	Nucleus	1H
22	Acquired Size	32768
23	Spectral Size	32768

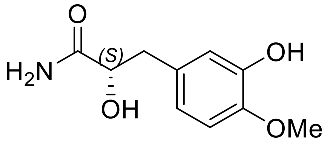

SI-4

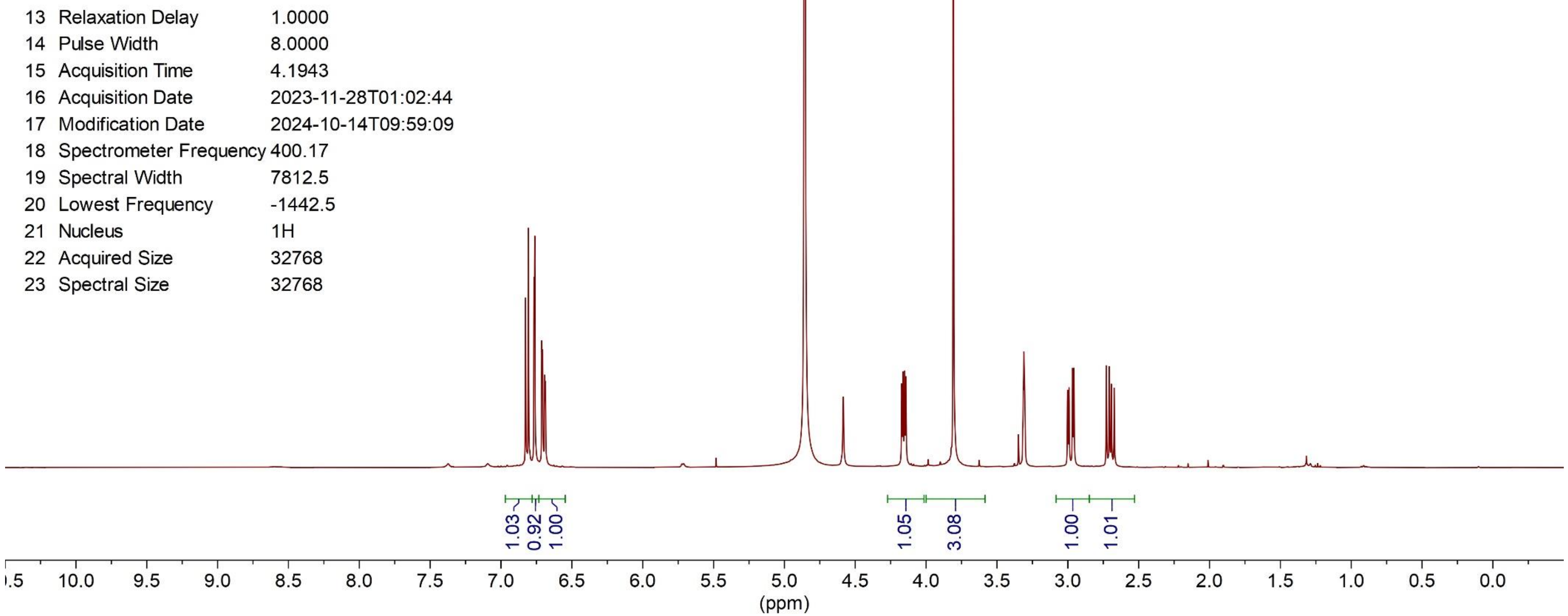

Parameters

	Parameter	Value
1	Sample Name	hZQMI3UsSYqxqLgNEpTmHQ.2.fid
2	Origin	Bruker BioSpin GmbH
3	Owner	nmrsu
4	Site	
5	Spectrometer	Avance
6	Author	
7	Solvent	MeOD
8	Temperature	298.0
9	Pulse Sequence	zgpg30
10	Experiment	1D
11	Number of Scans	3000
12	Receiver Gain	101
13	Relaxation Delay	2.0000
14	Pulse Width	8.0000
15	Acquisition Time	1.3763
16	Acquisition Date	2023-11-28T03:56:41
17	Modification Date	2024-10-14T09:59:09
18	Spectrometer Frequency	100.63
19	Spectral Width	23809.5
20	Lowest Frequency	-696.8
21	Nucleus	13C
22	Acquired Size	32768
23	Spectral Size	32768

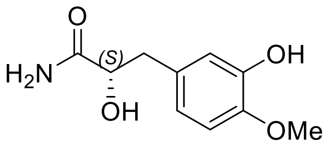

SI-4

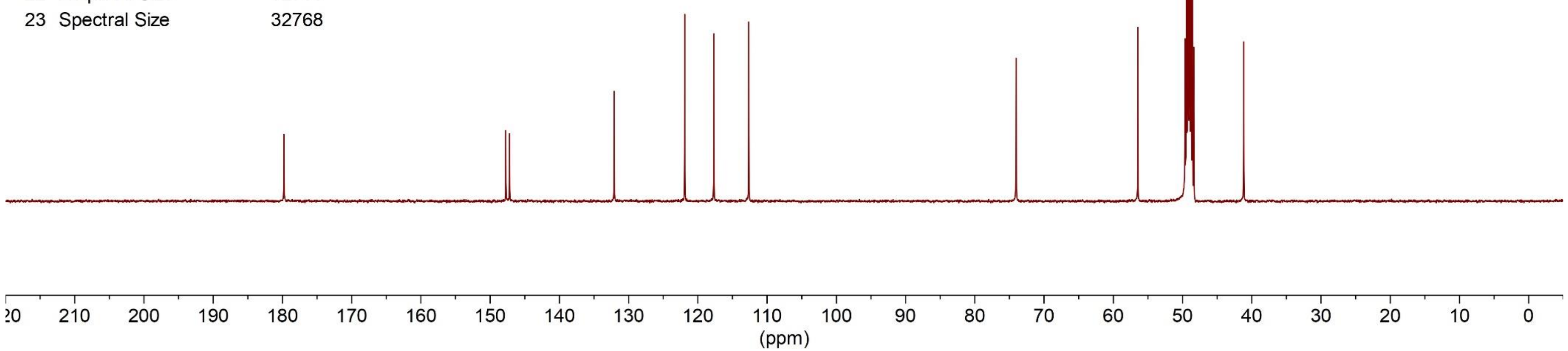

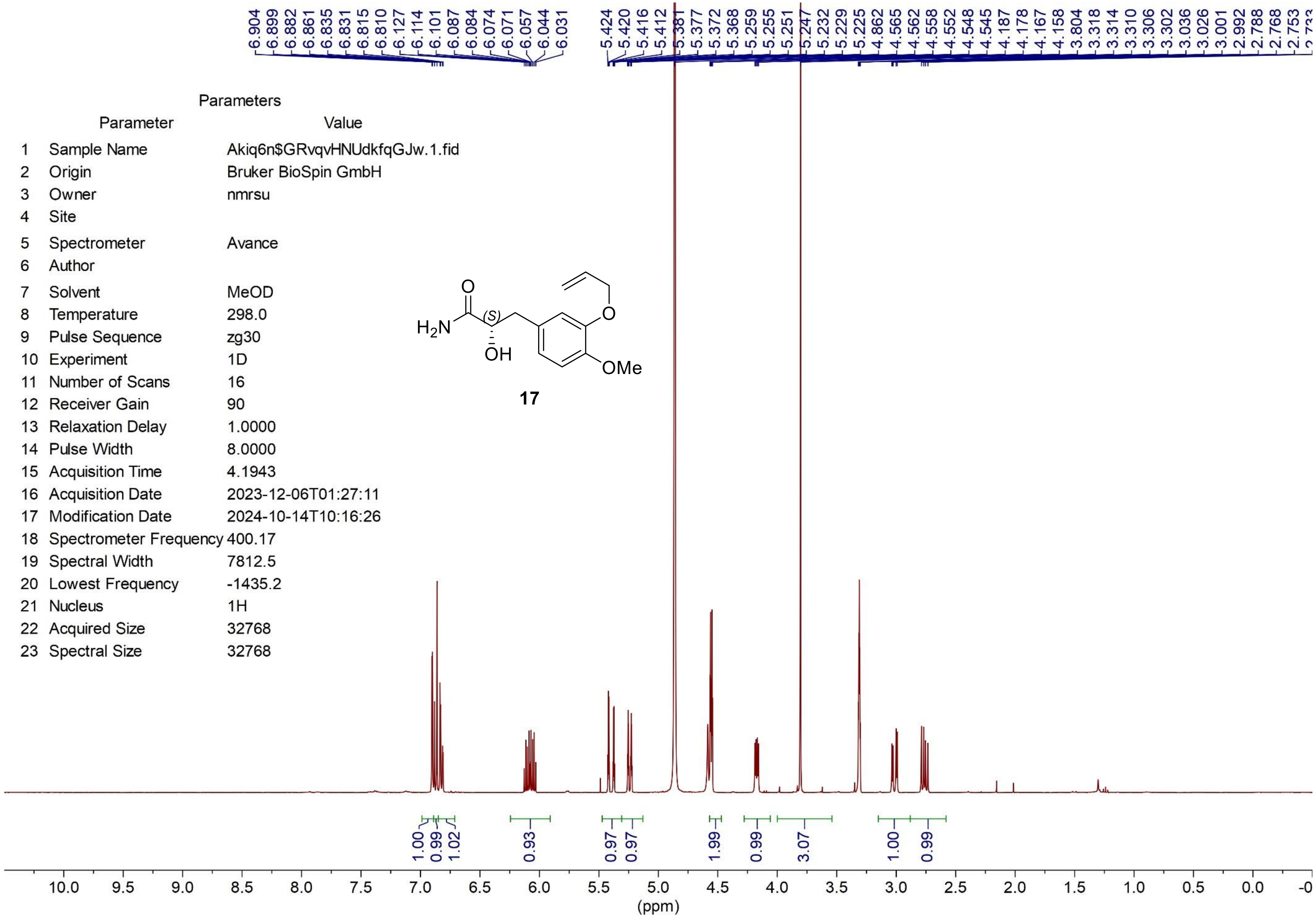
6.904
6.899
6.882
6.861
6.835
6.831
6.815
6.810
6.127
6.114
6.101
6.087
6.084
6.074
6.071
6.057
6.044
6.031
5.424
5.420
5.416
5.412
5.381
5.377
5.372
5.368
5.259
5.255
5.251
5.247
5.232
5.229
5.225
4.862
4.565
4.562
4.558
4.552
4.548
4.545
4.187
4.178
4.167
4.158
3.804
3.318
3.314
3.310
3.306
3.302
3.036
3.026
3.001
2.992
2.788
2.768
2.753
Parameters
Parameter Value
1 Sample Name Akiq6n$GRvqvHNUdkfqGJw.1.fid
2 Origin Bruker BioSpin GmbH
3 Owner nmrsu
4 Site
5 Spectrometer Avance
6 Author
7 Solvent MeOD
8 Temperature 298.0
9 Pulse Sequence zg30
10 Experiment 1D
11 Number of Scans 16
12 Receiver Gain 90
13 Relaxation Delay 1.0000
14 Pulse Width 8.0000
15 Acquisition Time 4.1943
16 Acquisition Date 2023-12-06T01:27:11
17 Modification Date 2024-10-14T10:16:26
18 Spectrometer Frequency 400.17
19 Spectral Width 7812.5
20 Lowest Frequency -1435.2
21 Nucleus 1H
22 Acquired Size 32768
23 Spectral Size 32768
O
H2N
(S)
OH
O
OMe
17
1.00
0.99
1.02
0.93
0.97
0.97
1.99
0.99
3.07
1.00
0.99
10.0 9.5 9.0 8.5 8.0 7.5 7.0 6.5 6.0 5.5 5.0 4.5 4.0 3.5 3.0 2.5 2.0 1.5 1.0 0.5 0.0 -0
(ppm)

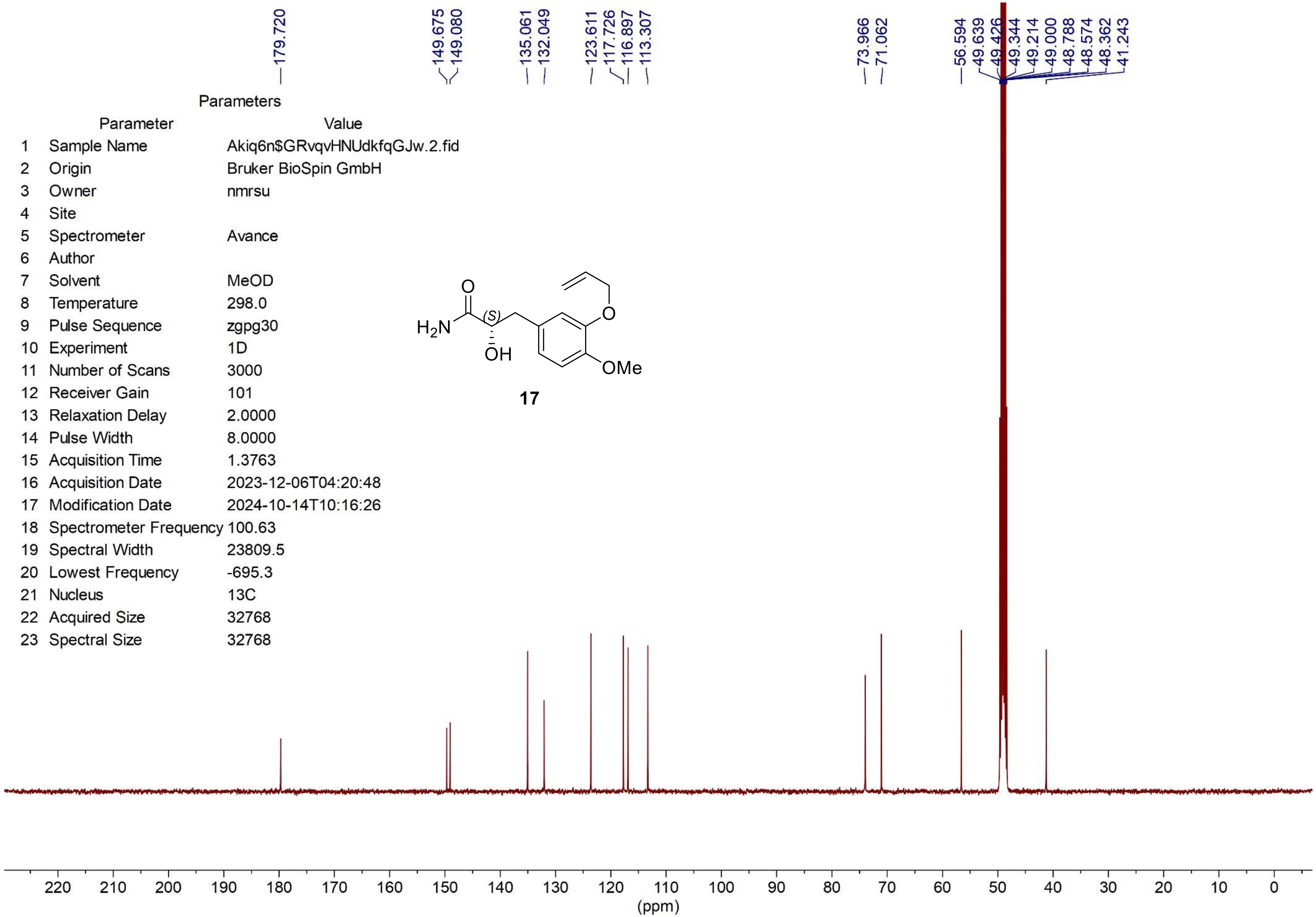

Parameters

	Parameter	Value
1	Sample Name	Akiq6n$GRvqvHNUdkfqGJw.2.fid
2	Origin	Bruker BioSpin GmbH
3	Owner	nmrsu
4	Site	
5	Spectrometer	Avance
6	Author	
7	Solvent	MeOD
8	Temperature	298.0
9	Pulse Sequence	zgpg30
10	Experiment	1D
11	Number of Scans	3000
12	Receiver Gain	101
13	Relaxation Delay	2.0000
14	Pulse Width	8.0000
15	Acquisition Time	1.3763
16	Acquisition Date	2023-12-06T04:20:48
17	Modification Date	2024-10-14T10:16:26
18	Spectrometer Frequency	100.63
19	Spectral Width	23809.5
20	Lowest Frequency	-695.3
21	Nucleus	13C
22	Acquired Size	32768
23	Spectral Size	32768

17

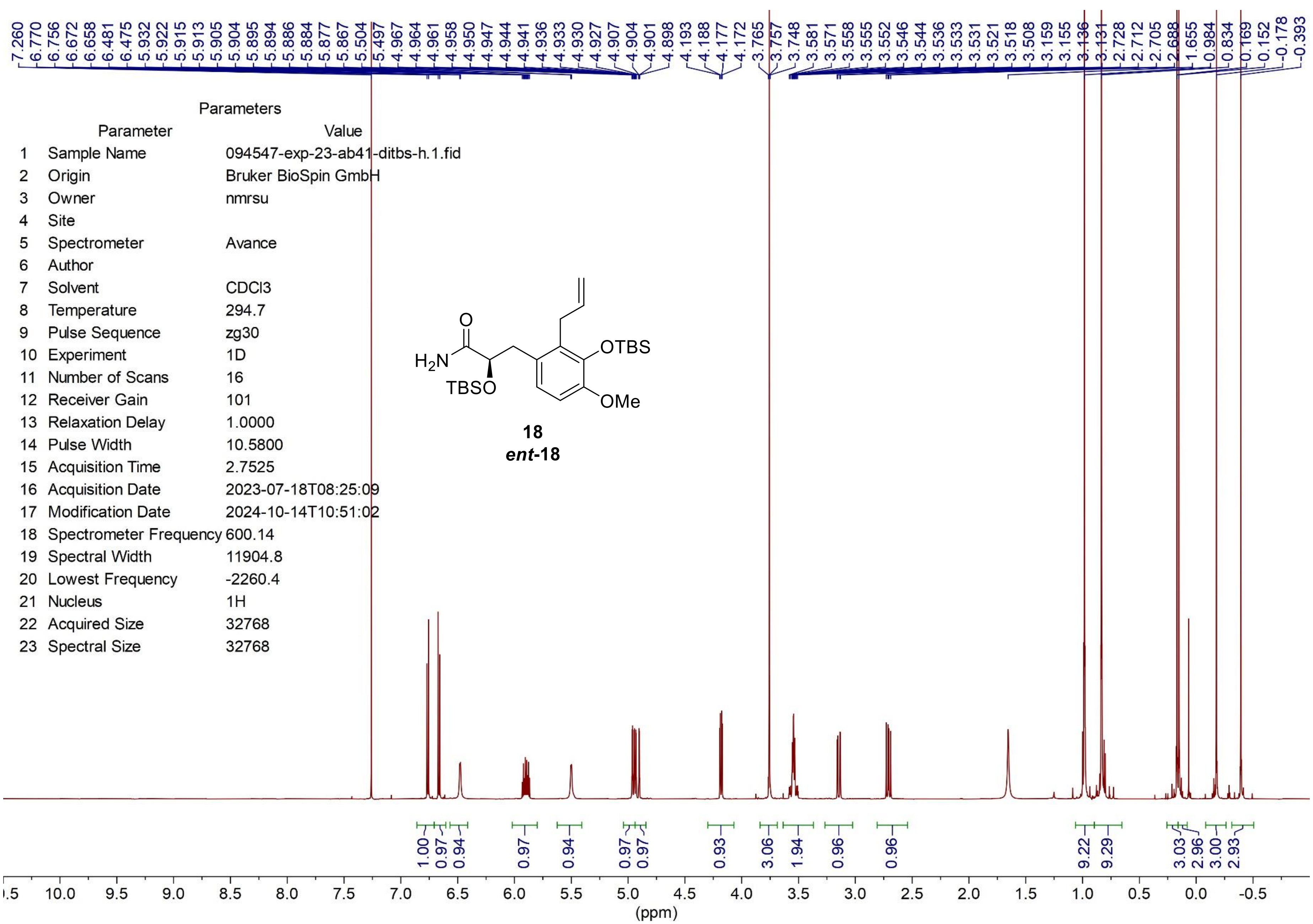
7.260 6.770 6.756 6.672 6.658 6.481 6.475 5.932 5.922 5.915 5.913 5.905 5.904 5.895 5.894 5.886 5.884 5.877 5.867 5.504 5.497 4.967 4.964 4.961 4.958 4.950 4.947 4.944 4.941 4.936 4.933 4.930 4.927 4.907 4.904 4.901 4.898 4.193 4.188 4.177 4.172 3.765 3.757 3.748 3.581 3.571 3.558 3.555 3.552 3.546 3.544 3.536 3.533 3.531 3.521 3.518 3.508 3.159 3.155 3.136 3.131 2.728 2.712 2.705 2.688 1.655 0.984 0.834 0.169 0.152 -0.178 -0.393
Parameters
Parameter Value
1 Sample Name 094547-exp-23-ab41-ditbs-h.1.fid
2 Origin Bruker BioSpin GmbH
3 Owner nmrsu
4 Site
5 Spectrometer Avance
6 Author
7 Solvent CDCl3
8 Temperature 294.7
9 Pulse Sequence zg30
10 Experiment 1D
11 Number of Scans 16
12 Receiver Gain 101
13 Relaxation Delay 1.0000
14 Pulse Width 10.5800
15 Acquisition Time 2.7525
16 Acquisition Date 2023-07-18T08:25:09
17 Modification Date 2024-10-14T10:51:02
18 Spectrometer Frequency 600.14
19 Spectral Width 11904.8
20 Lowest Frequency -2260.4
21 Nucleus 1H
22 Acquired Size 32768
23 Spectral Size 32768
O
H2N
TBSO
OTBS
OMe
18
ent-18
1.00 0.97 0.94 0.97 0.94 0.97 0.97 0.93 3.06 1.94 0.96 0.96 9.22 9.29 3.03 2.96 3.00 2.93
10.0 9.5 9.0 8.5 8.0 7.5 7.0 6.5 6.0 5.5 5.0 4.5 4.0 3.5 3.0 2.5 2.0 1.5 1.0 0.5 0.0 -0.5
(ppm)

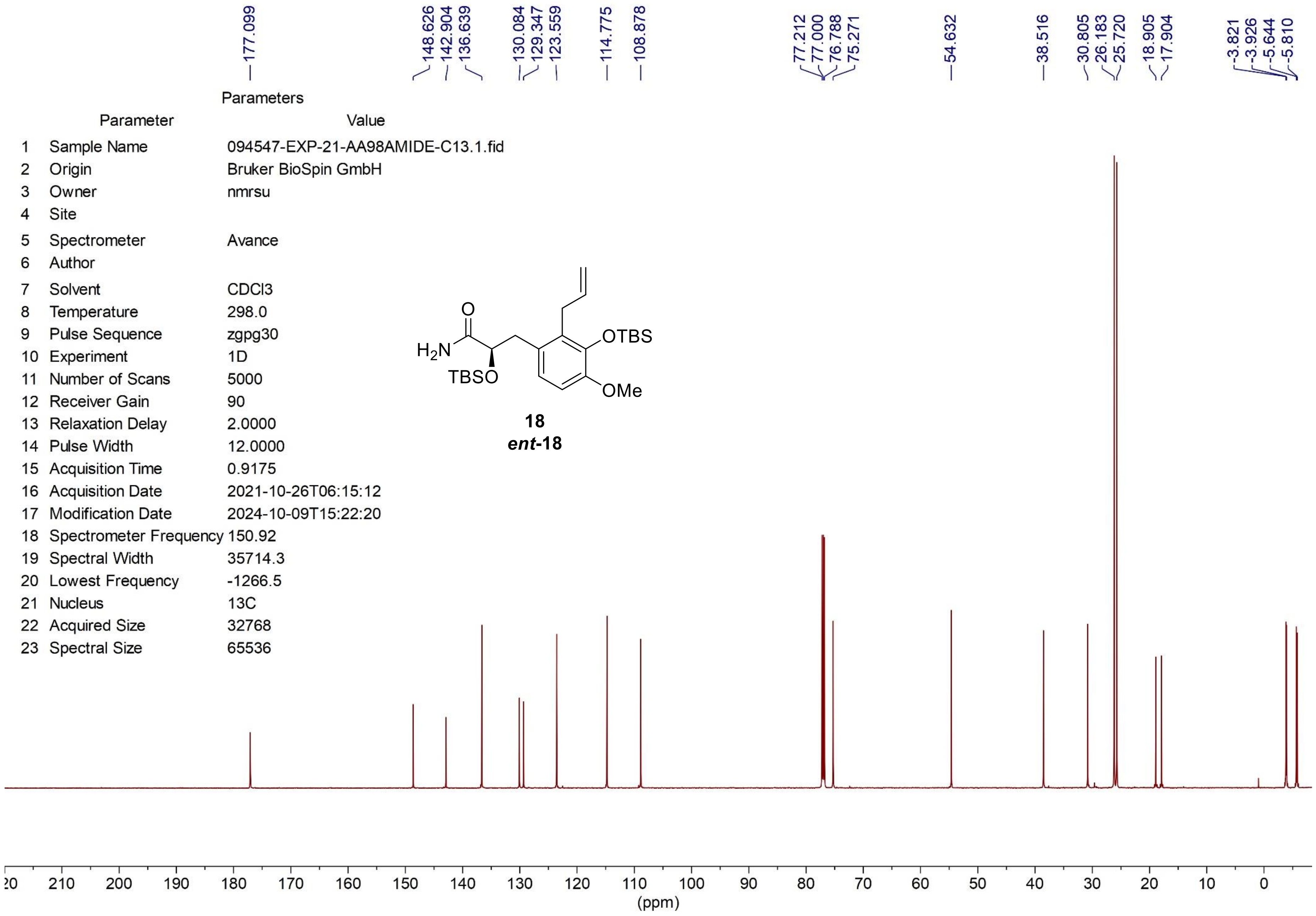

Parameters

	Parameter	Value
1	Sample Name	094547-EXP-21-AA98AMIDE-C13.1.fid
2	Origin	Bruker BioSpin GmbH
3	Owner	nmrsu
4	Site	
5	Spectrometer	Avance
6	Author	
7	Solvent	CDCl3
8	Temperature	298.0
9	Pulse Sequence	zgpg30
10	Experiment	1D
11	Number of Scans	5000
12	Receiver Gain	90
13	Relaxation Delay	2.0000
14	Pulse Width	12.0000
15	Acquisition Time	0.9175
16	Acquisition Date	2021-10-26T06:15:12
17	Modification Date	2024-10-09T15:22:20
18	Spectrometer Frequency	150.92
19	Spectral Width	35714.3
20	Lowest Frequency	-1266.5
21	Nucleus	13C
22	Acquired Size	32768
23	Spectral Size	65536

18
***ent*-18**

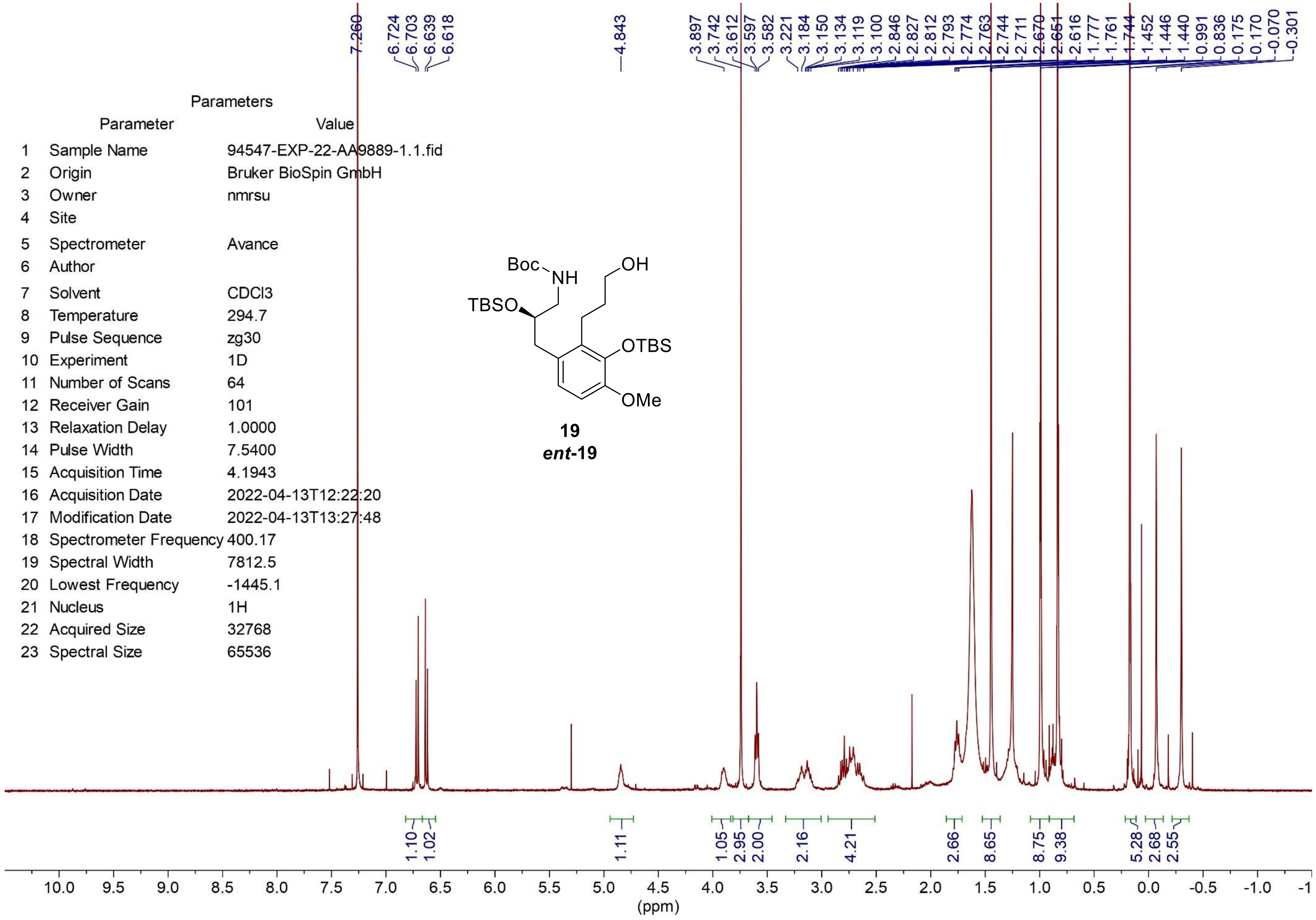
7.260
6.724
6.703
6.639
6.618
4.843
3.897
3.742
3.612
3.597
3.582
3.221
3.184
3.150
3.134
3.119
3.100
2.846
2.827
2.812
2.793
2.774
2.763
2.744
2.711
2.670
2.651
2.616
1.777
1.761
1.744
1.452
1.446
1.440
0.991
0.836
0.175
0.170
-0.070
-0.301
Parameters
Parameter Value
1 Sample Name 94547-EXP-22-AA9889-1.1.fid
2 Origin Bruker BioSpin GmbH
3 Owner nmrsu
4 Site
5 Spectrometer Avance
6 Author
7 Solvent CDCl3
8 Temperature 294.7
9 Pulse Sequence zg30
10 Experiment 1D
11 Number of Scans 64
12 Receiver Gain 101
13 Relaxation Delay 1.0000
14 Pulse Width 7.5400
15 Acquisition Time 4.1943
16 Acquisition Date 2022-04-13T12:22:20
17 Modification Date 2022-04-13T13:27:48
18 Spectrometer Frequency 400.17
19 Spectral Width 7812.5
20 Lowest Frequency -1445.1
21 Nucleus 1H
22 Acquired Size 32768
23 Spectral Size 65536
Boc
NH
OH
TBSO
OTBS
OMe
19
ent-19
1.10
1.02
1.11
1.05
2.95
2.00
2.16
4.21
2.66
8.65
8.75
9.38
5.28
2.68
2.55
10.0 9.5 9.0 8.5 8.0 7.5 7.0 6.5 6.0 5.5 5.0 4.5 4.0 3.5 3.0 2.5 2.0 1.5 1.0 0.5 0.0 -0.5 -1.0 -1
(ppm)

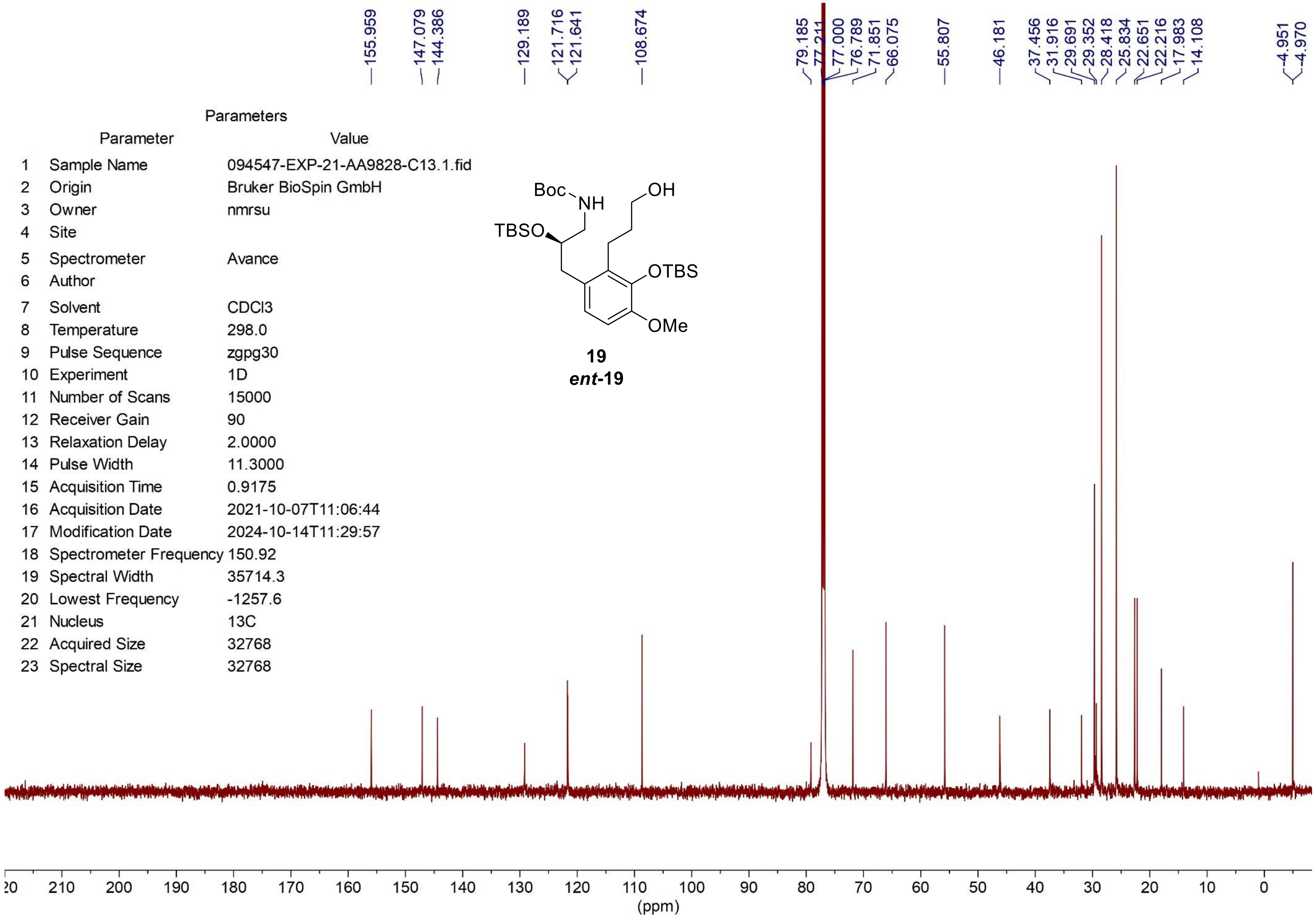

Parameters

	Parameter	Value
1	Sample Name	094547-EXP-21-AA9828-C13.1.fid
2	Origin	Bruker BioSpin GmbH
3	Owner	nmrsu
4	Site	
5	Spectrometer	Avance
6	Author	
7	Solvent	CDCl3
8	Temperature	298.0
9	Pulse Sequence	zgpg30
10	Experiment	1D
11	Number of Scans	15000
12	Receiver Gain	90
13	Relaxation Delay	2.0000
14	Pulse Width	11.3000
15	Acquisition Time	0.9175
16	Acquisition Date	2021-10-07T11:06:44
17	Modification Date	2024-10-14T11:29:57
18	Spectrometer Frequency	150.92
19	Spectral Width	35714.3
20	Lowest Frequency	-1257.6
21	Nucleus	13C
22	Acquired Size	32768
23	Spectral Size	32768

19
***ent*-19**

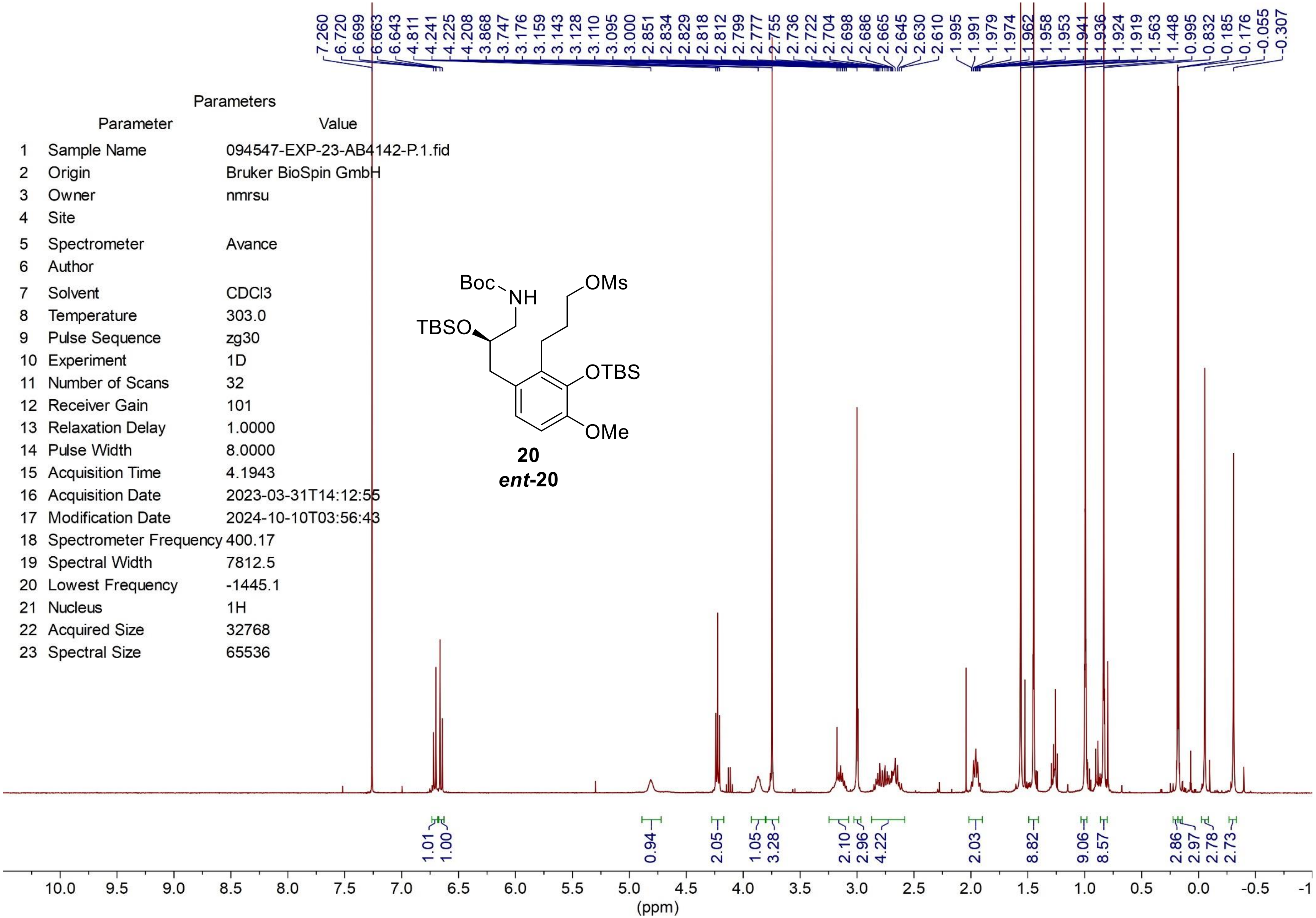
7.260 6.720 6.699 6.663 6.643 4.811 4.241 4.225 4.208 3.868 3.747 3.176 3.159 3.143 3.128 3.110 3.095 3.000 2.851 2.834 2.829 2.818 2.812 2.799 2.777 2.755 2.736 2.722 2.704 2.698 2.686 2.665 2.645 2.630 2.610 1.995 1.991 1.979 1.974 1.962 1.958 1.953 1.941 1.936 1.924 1.919 1.563 1.448 0.995 0.832 0.185 0.176 -0.055 -0.307
Parameters
Parameter Value
1 Sample Name 094547-EXP-23-AB4142-P.1.fid
2 Origin Bruker BioSpin GmbH
3 Owner nmrsu
4 Site
5 Spectrometer Avance
6 Author
7 Solvent CDCl3
8 Temperature 303.0
9 Pulse Sequence zg30
10 Experiment 1D
11 Number of Scans 32
12 Receiver Gain 101
13 Relaxation Delay 1.0000
14 Pulse Width 8.0000
15 Acquisition Time 4.1943
16 Acquisition Date 2023-03-31T14:12:55
17 Modification Date 2024-10-10T03:56:43
18 Spectrometer Frequency 400.17
19 Spectral Width 7812.5
20 Lowest Frequency -1445.1
21 Nucleus 1H
22 Acquired Size 32768
23 Spectral Size 65536
Boc NH OMs
TBSO
OTBS
OMe
20
ent-20
1.01 1.00 0.94 2.05 1.05 3.28 2.10 2.96 4.22 2.03 8.82 9.06 8.57 2.86 2.97 2.78 2.73
10.0 9.5 9.0 8.5 8.0 7.5 7.0 6.5 6.0 5.5 5.0 4.5 4.0 3.5 3.0 2.5 2.0 1.5 1.0 0.5 0.0 -0.5 -1
(ppm)

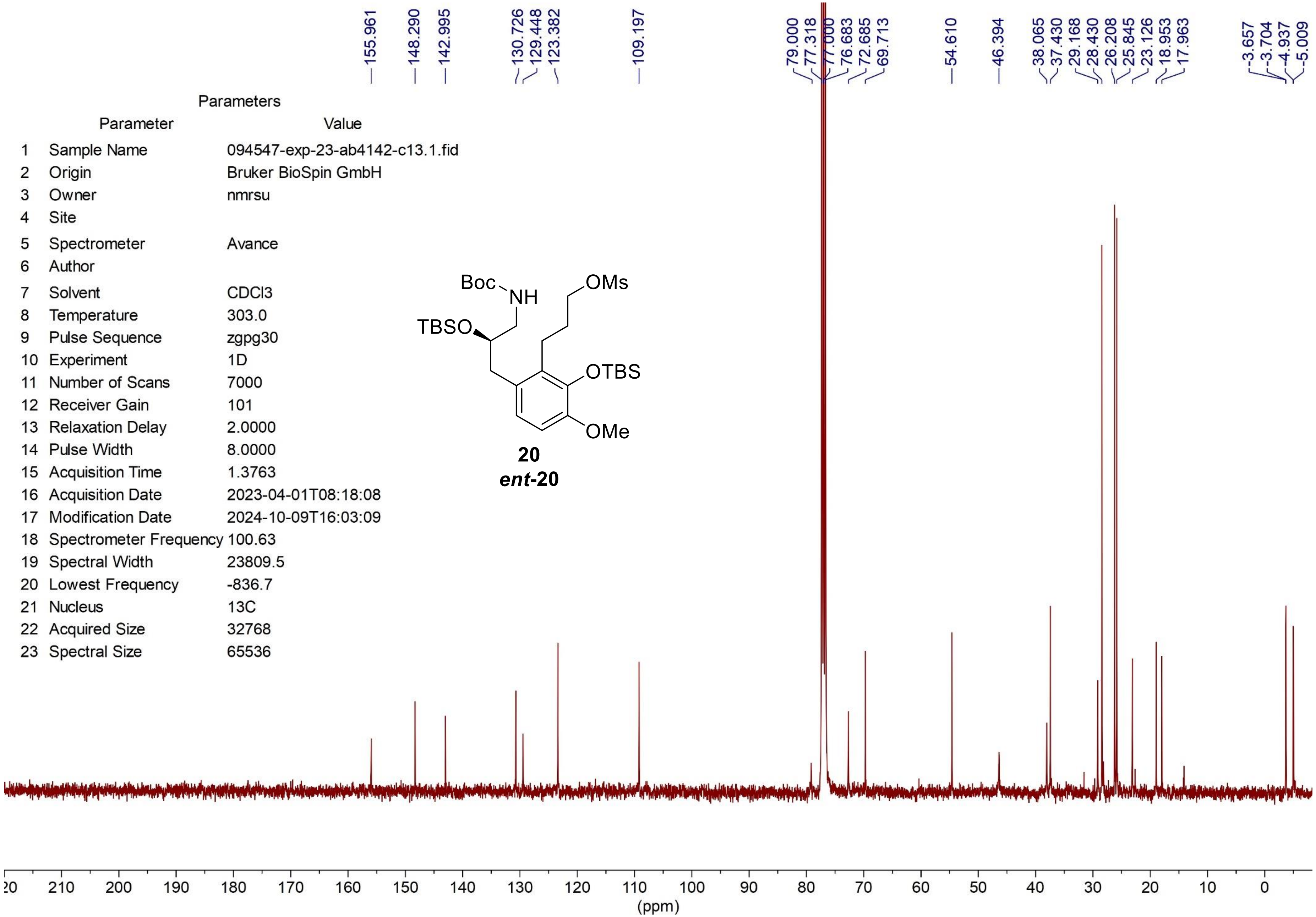

Parameters

	Parameter	Value
1	Sample Name	094547-exp-23-ab4142-c13.1.fid
2	Origin	Bruker BioSpin GmbH
3	Owner	nmrsu
4	Site	
5	Spectrometer	Avance
6	Author	
7	Solvent	CDCl3
8	Temperature	303.0
9	Pulse Sequence	zgpg30
10	Experiment	1D
11	Number of Scans	7000
12	Receiver Gain	101
13	Relaxation Delay	2.0000
14	Pulse Width	8.0000
15	Acquisition Time	1.3763
16	Acquisition Date	2023-04-01T08:18:08
17	Modification Date	2024-10-09T16:03:09
18	Spectrometer Frequency	100.63
19	Spectral Width	23809.5
20	Lowest Frequency	-836.7
21	Nucleus	13C
22	Acquired Size	32768
23	Spectral Size	65536

20

***ent*-20**

Parameters

	Parameter	Value
1	Sample Name	094547-EXP-22-AA9870.1.fid
2	Origin	Bruker BioSpin GmbH
3	Owner	nmrsu
4	Site	
5	Spectrometer	Avance
6	Author	
7	Solvent	CDCl3
8	Temperature	294.7
9	Pulse Sequence	zg30
10	Experiment	1D
11	Number of Scans	32
12	Receiver Gain	101
13	Relaxation Delay	1.0000
14	Pulse Width	7.5400
15	Acquisition Time	4.1943
16	Acquisition Date	2022-03-09T17:26:57
17	Modification Date	2022-03-09T17:34:31
18	Spectrometer Frequency	400.17
19	Spectral Width	7812.5
20	Lowest Frequency	-1445.3
21	Nucleus	1H
22	Acquired Size	32768
23	Spectral Size	65536

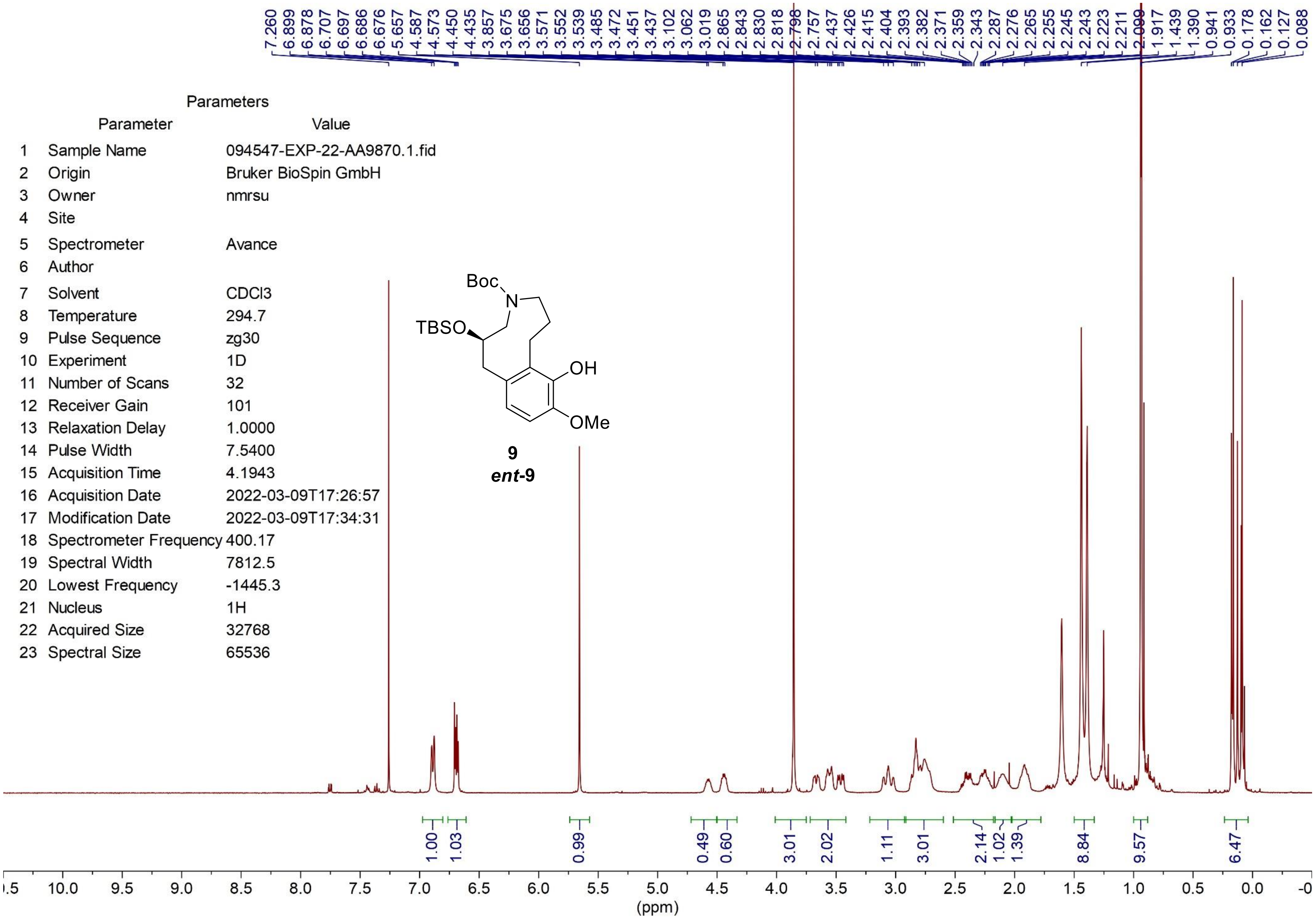

Parameters

	Parameter	Value
1	Sample Name	v1PVTqApQ8qTtTibDHP6hg.2.fid
2	Origin	Bruker BioSpin GmbH
3	Owner	nmrsu
4	Site	
5	Spectrometer	Avance
6	Author	
7	Solvent	CDCl3
8	Temperature	298.0
9	Pulse Sequence	zgpg30
10	Experiment	1D
11	Number of Scans	15000
12	Receiver Gain	101
13	Relaxation Delay	2.0000
14	Pulse Width	12.0000
15	Acquisition Time	0.9175
16	Acquisition Date	2021-10-21T04:47:50
17	Modification Date	2024-10-14T15:44:57
18	Spectrometer Frequency	150.92
19	Spectral Width	35714.3
20	Lowest Frequency	-1259.4
21	Nucleus	13C
22	Acquired Size	32768
23	Spectral Size	32768

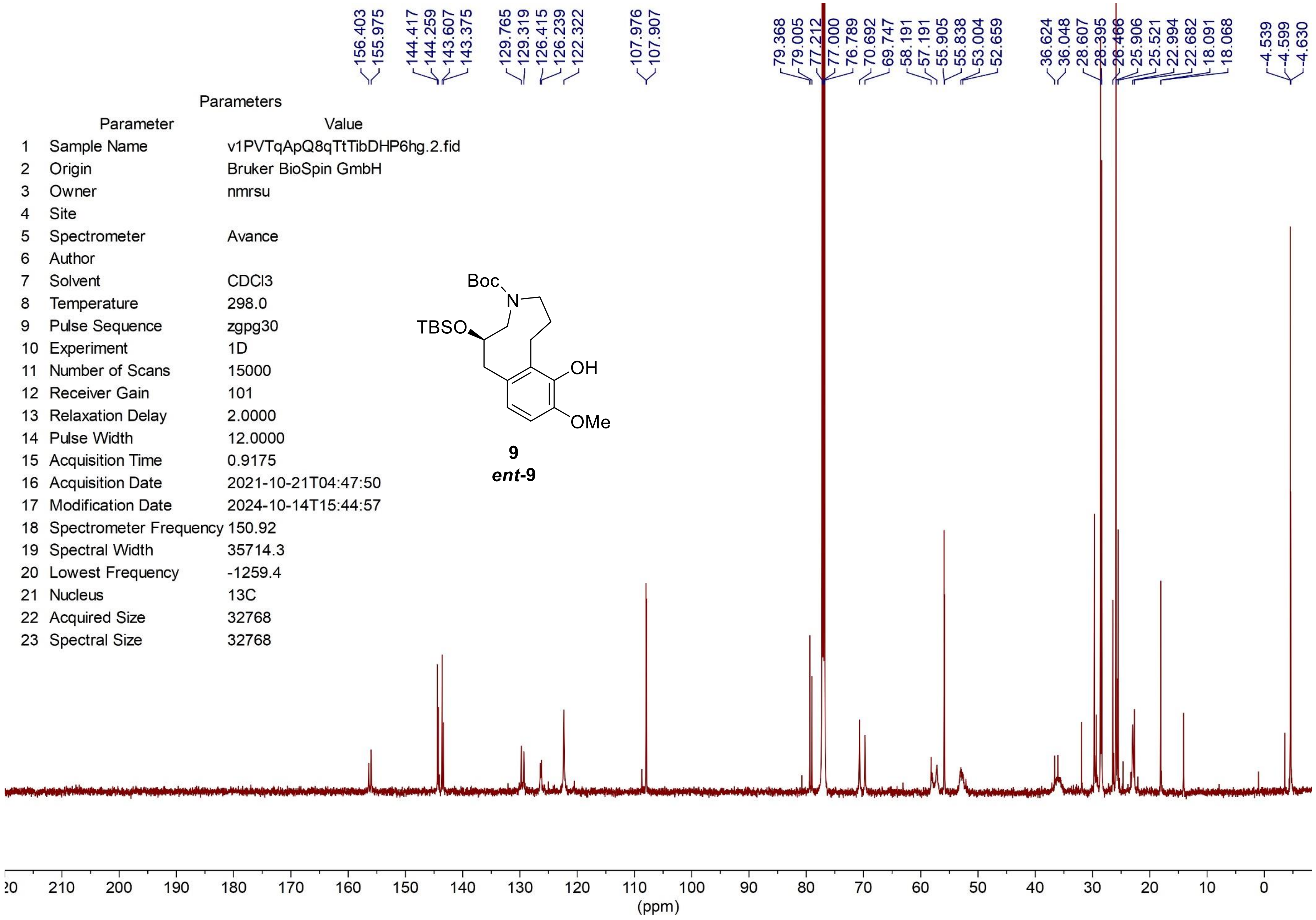

9
***ent*-9**

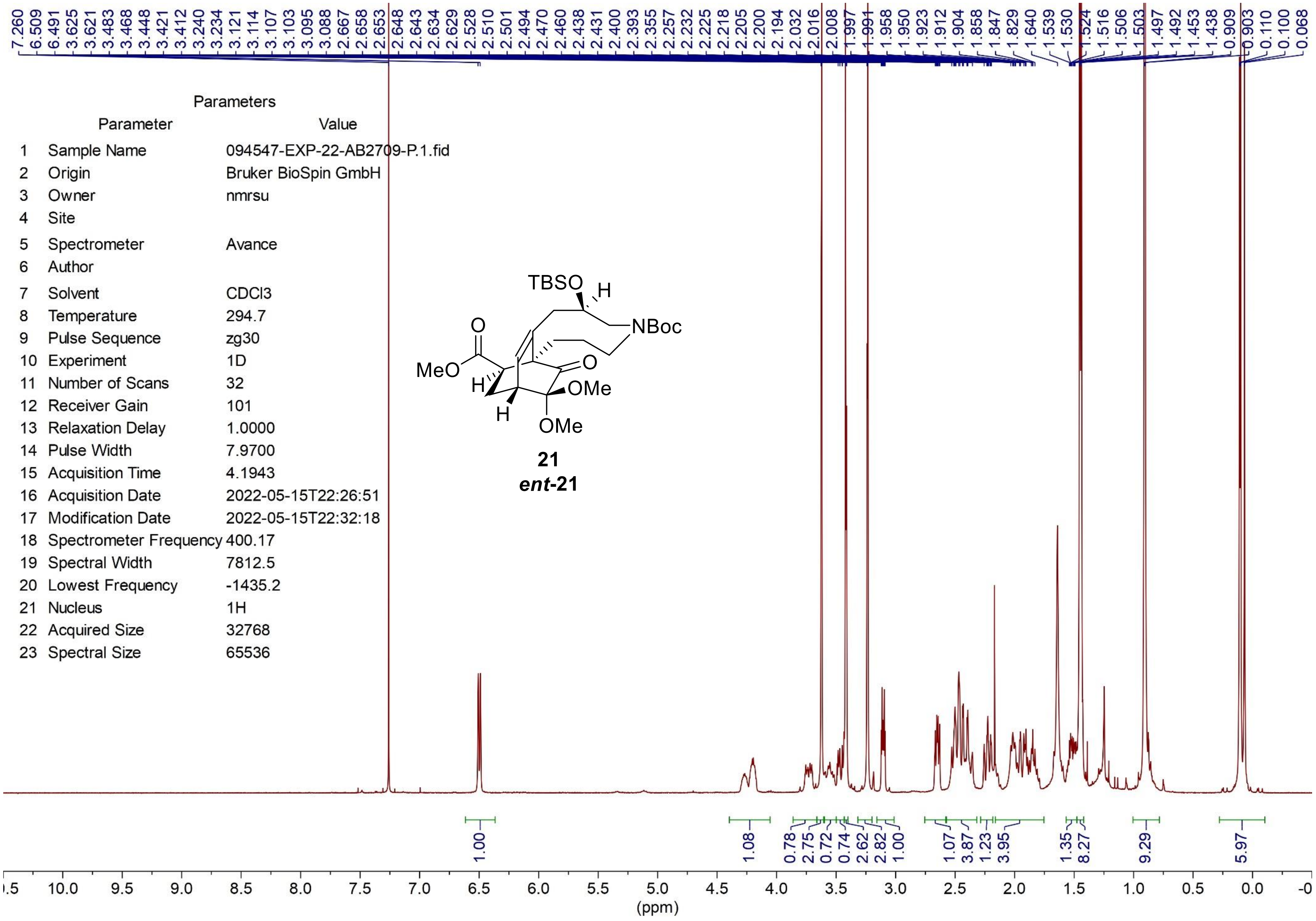

21
***ent*-21**

Parameters

	Parameter	Value
1	Sample Name	094547-EXP-22-AB2709-P.1.fid
2	Origin	Bruker BioSpin GmbH
3	Owner	nmrsu
4	Site	
5	Spectrometer	Avance
6	Author	
7	Solvent	CDCl3
8	Temperature	294.7
9	Pulse Sequence	zg30
10	Experiment	1D
11	Number of Scans	32
12	Receiver Gain	101
13	Relaxation Delay	1.0000
14	Pulse Width	7.9700
15	Acquisition Time	4.1943
16	Acquisition Date	2022-05-15T22:26:51
17	Modification Date	2022-05-15T22:32:18
18	Spectrometer Frequency	400.17
19	Spectral Width	7812.5
20	Lowest Frequency	-1435.2
21	Nucleus	1H
22	Acquired Size	32768
23	Spectral Size	65536

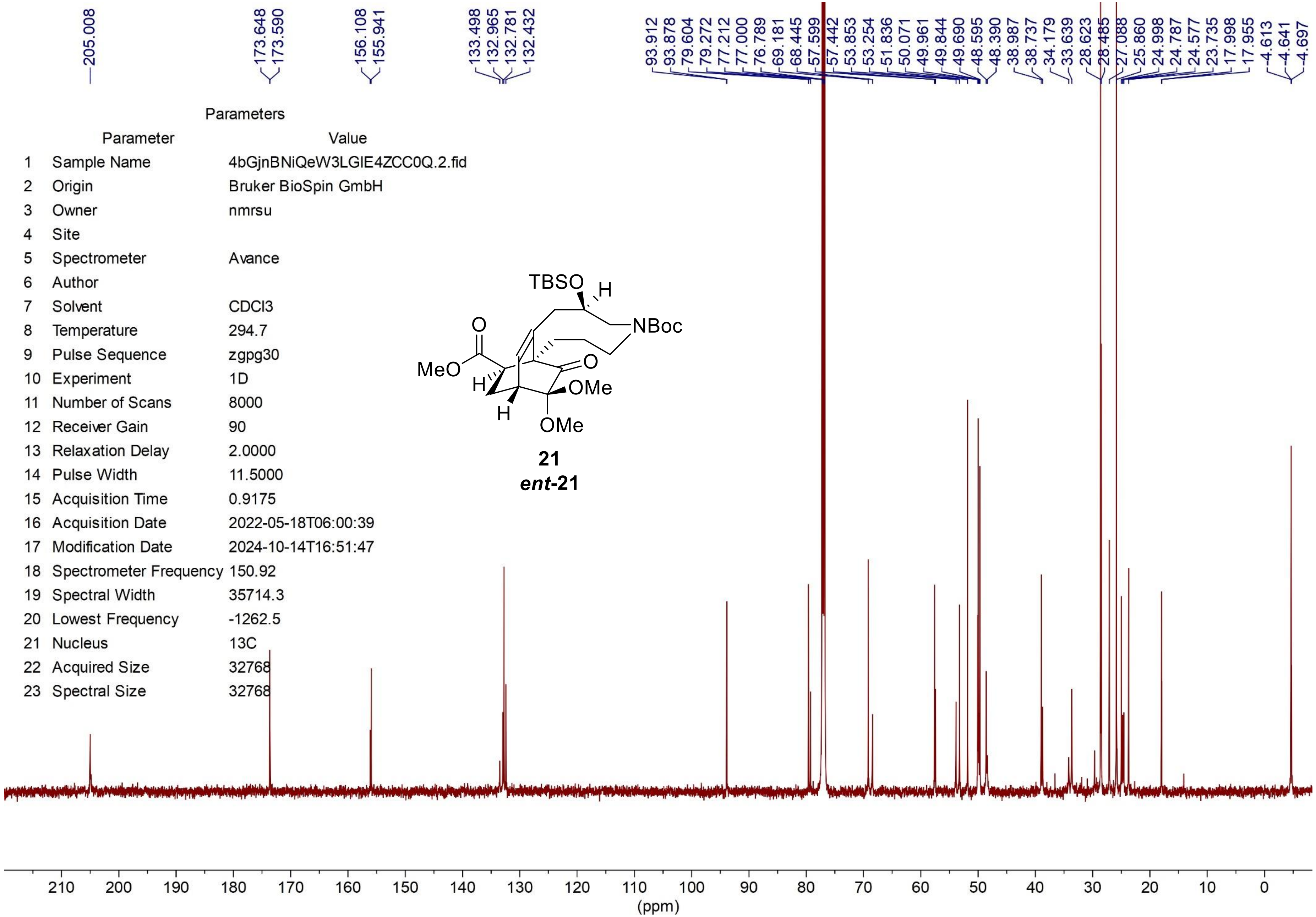

Parameters

	Parameter	Value
1	Sample Name	4bGjnBNiQeW3LGlE4ZCC0Q.2.fid
2	Origin	Bruker BioSpin GmbH
3	Owner	nmrsu
4	Site	
5	Spectrometer	Avance
6	Author	
7	Solvent	CDCl3
8	Temperature	294.7
9	Pulse Sequence	zgpg30
10	Experiment	1D
11	Number of Scans	8000
12	Receiver Gain	90
13	Relaxation Delay	2.0000
14	Pulse Width	11.5000
15	Acquisition Time	0.9175
16	Acquisition Date	2022-05-18T06:00:39
17	Modification Date	2024-10-14T16:51:47
18	Spectrometer Frequency	150.92
19	Spectral Width	35714.3
20	Lowest Frequency	-1262.5
21	Nucleus	13C
22	Acquired Size	32768
23	Spectral Size	32768

Parameters

	Parameter	Value
1	Sample Name	094547-exp-22-ab2765-aldehyde.1.fid
2	Origin	Bruker BioSpin GmbH
3	Owner	nmrsu
4	Site	
5	Spectrometer	Avance
6	Author	
7	Solvent	CDCl3
8	Temperature	294.7
9	Pulse Sequence	zg30
10	Experiment	1D
11	Number of Scans	32
12	Receiver Gain	74
13	Relaxation Delay	1.0000
14	Pulse Width	8.0000
15	Acquisition Time	4.1943
16	Acquisition Date	2022-10-06T17:02:42
17	Modification Date	2022-10-06T17:34:30
18	Spectrometer Frequency	400.17
19	Spectral Width	7812.5
20	Lowest Frequency	-1444.5
21	Nucleus	1H
22	Acquired Size	32768
23	Spectral Size	65536

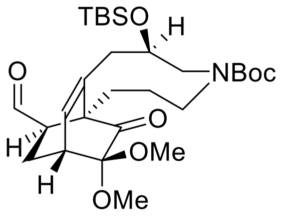

7

***ent*-7**

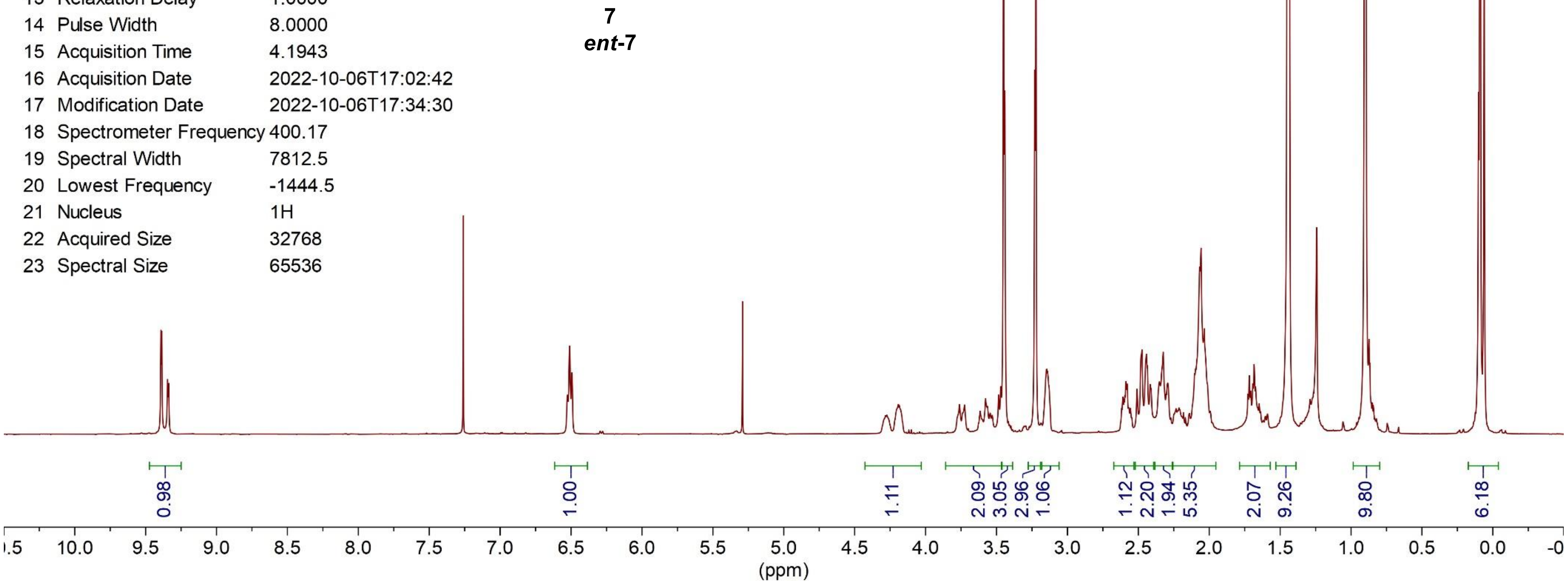

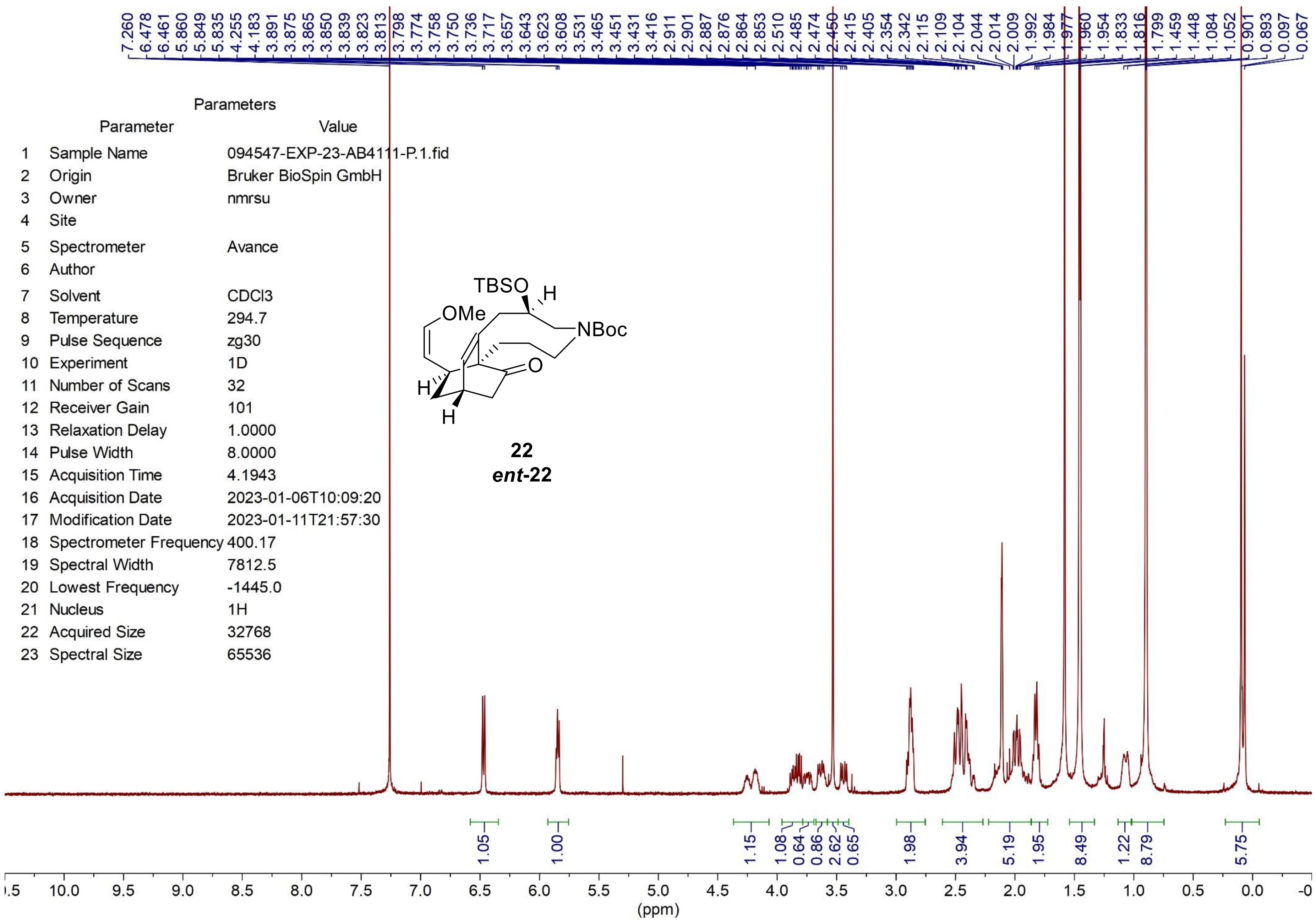
7.260
6.478
6.461
5.860
5.849
5.835
4.255
4.183
3.891
3.875
3.865
3.850
3.839
3.823
3.813
3.798
3.774
3.758
3.750
3.736
3.717
3.657
3.643
3.623
3.608
3.531
3.465
3.451
3.431
3.416
2.911
2.901
2.887
2.876
2.864
2.853
2.510
2.485
2.474
2.450
2.415
2.405
2.354
2.342
2.115
2.109
2.104
2.044
2.014
2.009
1.992
1.984
1.977
1.960
1.954
1.833
1.816
1.799
1.459
1.448
1.084
1.052
0.901
0.893
0.097
0.067
Parameters
Parameter
Value
1 Sample Name 094547-EXP-23-AB4111-P.1.fid
2 Origin Bruker BioSpin GmbH
3 Owner nmrsu
4 Site
5 Spectrometer Avance
6 Author
7 Solvent CDCl3
8 Temperature 294.7
9 Pulse Sequence zg30
10 Experiment 1D
11 Number of Scans 32
12 Receiver Gain 101
13 Relaxation Delay 1.0000
14 Pulse Width 8.0000
15 Acquisition Time 4.1943
16 Acquisition Date 2023-01-06T10:09:20
17 Modification Date 2023-01-11T21:57:30
18 Spectrometer Frequency 400.17
19 Spectral Width 7812.5
20 Lowest Frequency -1445.0
21 Nucleus 1H
22 Acquired Size 32768
23 Spectral Size 65536
TBSO
H
OMe
NBoc
O
H
H
22
ent-22
1.05
1.00
1.15
1.08
0.64
0.86
2.62
0.65
1.98
3.94
5.19
1.95
8.49
1.22
8.79
5.75
10.0
9.5
9.0
8.5
8.0
7.5
7.0
6.5
6.0
5.5
5.0
4.5
4.0
3.5
3.0
2.5
2.0
1.5
1.0
0.5
0.0
(ppm)

Parameters

	Parameter	Value
1	Sample Name	094547-EXP-22-AB2773-SPOT2.1.fid
2	Origin	Bruker BioSpin GmbH
3	Owner	nmrsu
4	Site	
5	Spectrometer	Avance
6	Author	
7	Solvent	CDCl3
8	Temperature	294.7
9	Pulse Sequence	zg30
10	Experiment	1D
11	Number of Scans	32
12	Receiver Gain	46
13	Relaxation Delay	1.0000
14	Pulse Width	8.0000
15	Acquisition Time	4.1943
16	Acquisition Date	2022-10-20T19:51:26
17	Modification Date	2022-10-25T16:24:19
18	Spectrometer Frequency	400.17
19	Spectral Width	7812.5
20	Lowest Frequency	-1435.2
21	Nucleus	1H
22	Acquired Size	32768
23	Spectral Size	65536

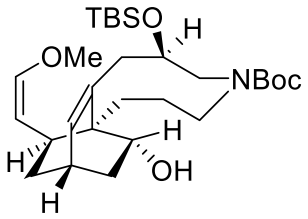

SI-6
***ent*-SI-6**

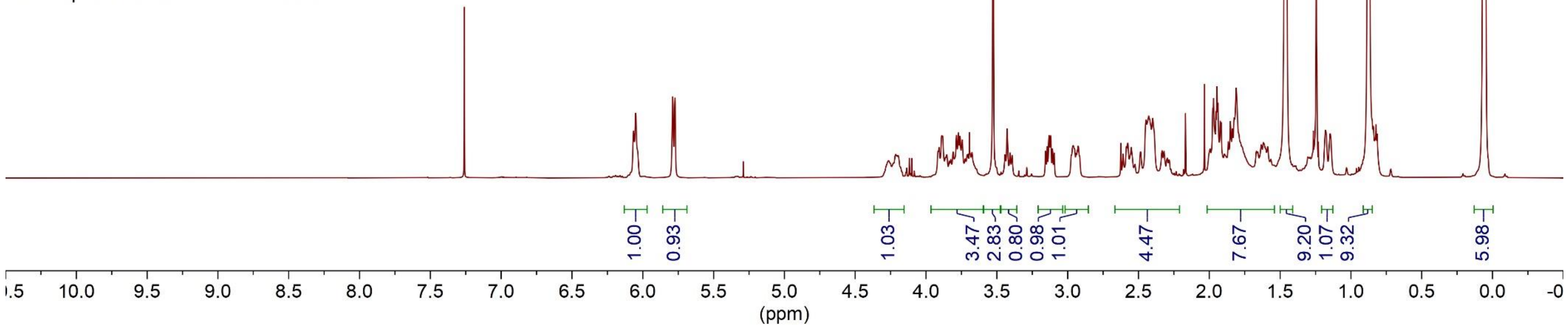

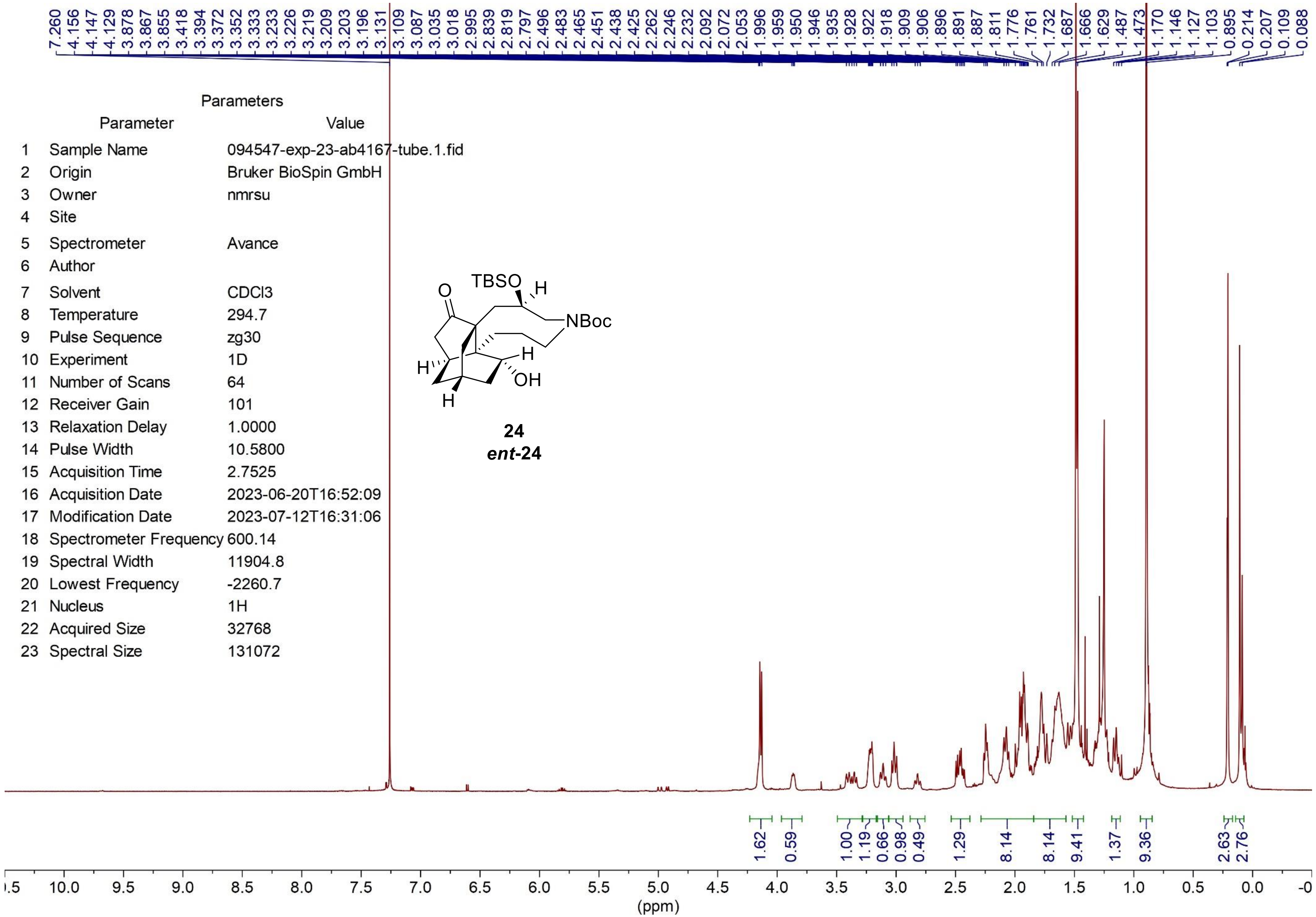
7.260 4.156 4.147 4.129 3.878 3.867 3.855 3.418 3.394 3.372 3.352 3.333 3.233 3.226 3.219 3.209 3.203 3.196 3.131 3.109 3.087 3.035 3.018 2.995 2.839 2.819 2.797 2.496 2.483 2.465 2.451 2.438 2.425 2.262 2.246 2.232 2.092 2.072 2.053 1.996 1.959 1.950 1.946 1.935 1.928 1.922 1.918 1.909 1.906 1.896 1.891 1.887 1.811 1.776 1.761 1.732 1.687 1.666 1.629 1.487 1.473 1.170 1.146 1.127 1.103 0.895 0.214 0.207 0.109 0.088
Parameters
Parameter Value
1 Sample Name 094547-exp-23-ab4167-tube.1.fid
2 Origin Bruker BioSpin GmbH
3 Owner nmrsu
4 Site
5 Spectrometer Avance
6 Author
7 Solvent CDCl3
8 Temperature 294.7
9 Pulse Sequence zg30
10 Experiment 1D
11 Number of Scans 64
12 Receiver Gain 101
13 Relaxation Delay 1.0000
14 Pulse Width 10.5800
15 Acquisition Time 2.7525
16 Acquisition Date 2023-06-20T16:52:09
17 Modification Date 2023-07-12T16:31:06
18 Spectrometer Frequency 600.14
19 Spectral Width 11904.8
20 Lowest Frequency -2260.7
21 Nucleus 1H
22 Acquired Size 32768
23 Spectral Size 131072
O
TBSO
H
NBoc
H
H
OH
H
24
ent-24
1.62 0.59 1.00 1.19 0.66 0.98 0.49 1.29 8.14 8.14 9.41 1.37 9.36 2.63 2.76
10.0 9.5 9.0 8.5 8.0 7.5 7.0 6.5 6.0 5.5 5.0 4.5 4.0 3.5 3.0 2.5 2.0 1.5 1.0 0.5 0.0
(ppm)

Parameters

	Parameter	Value
1	Sample Name	094547-exp-23-ab4167-c13.1.fid
2	Origin	Bruker BioSpin GmbH
3	Owner	nmrsu
4	Site	
5	Spectrometer	Avance
6	Author	
7	Solvent	CDCl3
8	Temperature	294.7
9	Pulse Sequence	zgpg30
10	Experiment	1D
11	Number of Scans	8000
12	Receiver Gain	101
13	Relaxation Delay	2.0000
14	Pulse Width	12.0000
15	Acquisition Time	0.9175
16	Acquisition Date	2023-06-20T23:48:33
17	Modification Date	2024-10-09T19:57:22
18	Spectrometer Frequency	150.92
19	Spectral Width	35714.3
20	Lowest Frequency	-1261.9
21	Nucleus	13C
22	Acquired Size	32768
23	Spectral Size	65536

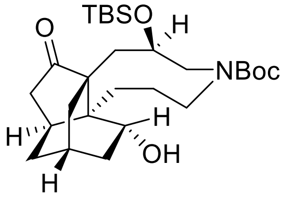

24
***ent*-24**

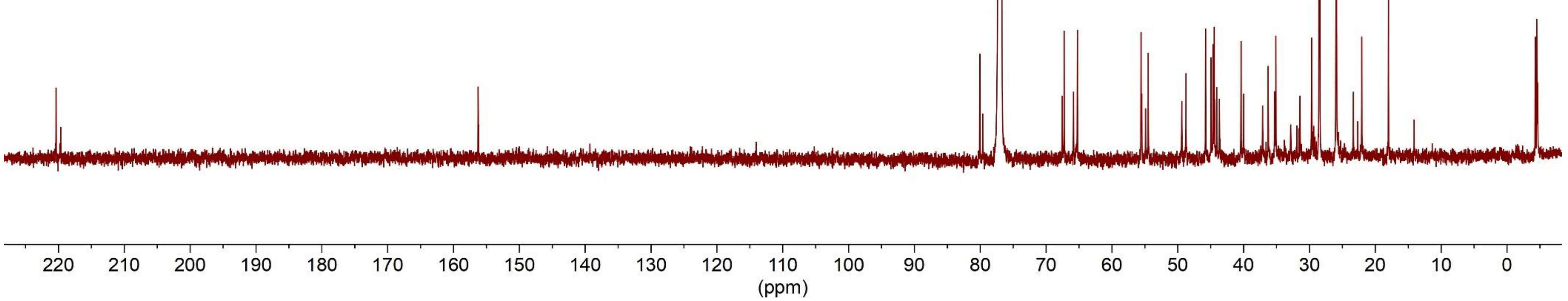

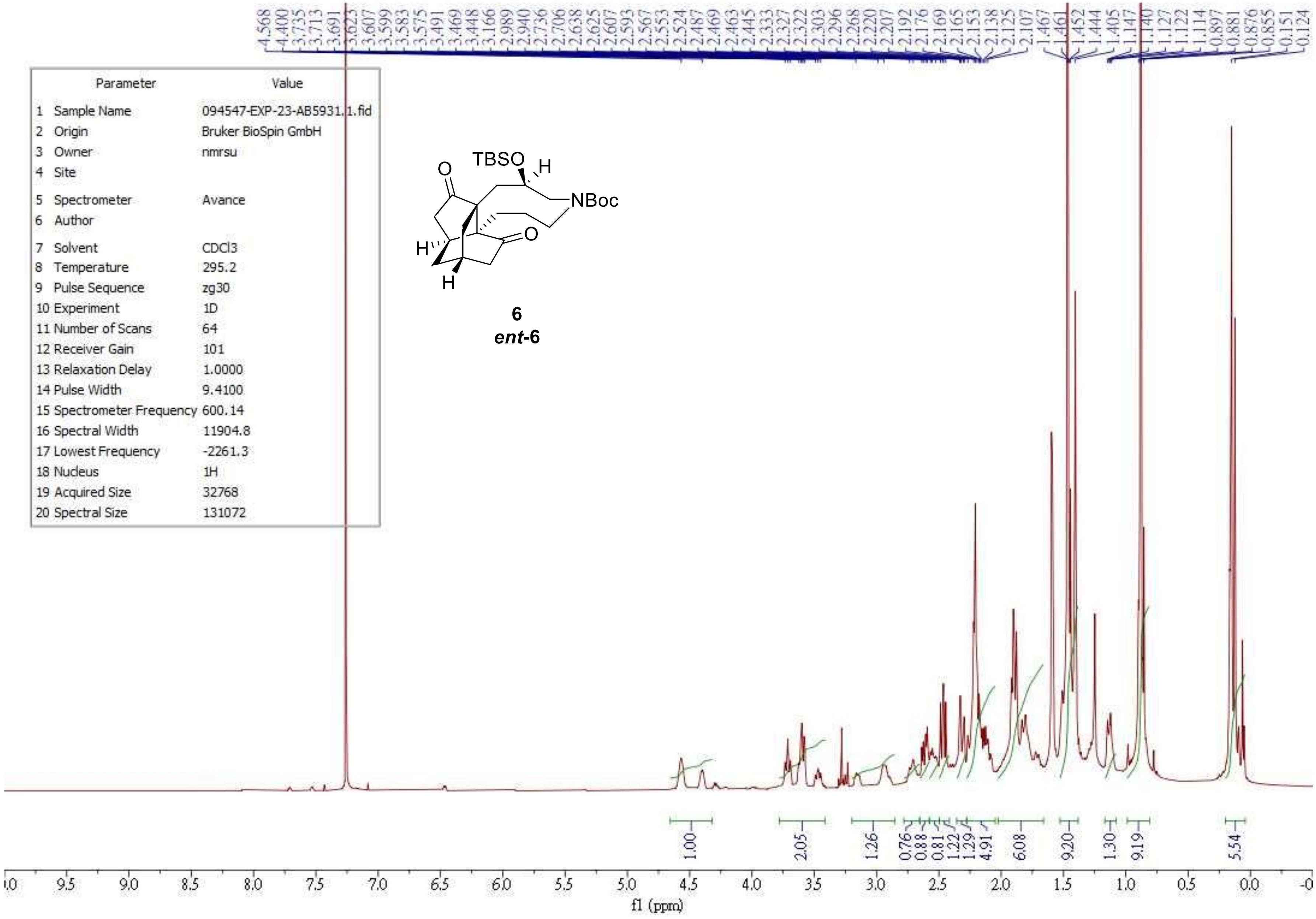
4.568 4.400 3.735 3.713 3.691 3.623 3.607 3.599 3.583 3.575 3.491 3.469 3.448 3.166 2.989 2.940 2.736 2.706 2.638 2.625 2.607 2.593 2.567 2.553 2.524 2.487 2.469 2.463 2.445 2.333 2.327 2.322 2.303 2.296 2.268 2.220 2.207 2.192 2.176 2.169 2.165 2.153 2.138 2.125 2.107 1.467 1.461 1.452 1.444 1.405 1.147 1.140 1.127 1.122 1.114 0.897 0.881 0.876 0.855 0.151 0.124
Parameter Value
1 Sample Name 094547-EXP-23-AB5931.1.fid
2 Origin Bruker BioSpin GmbH
3 Owner nmrsu
4 Site
5 Spectrometer Avance
6 Author
7 Solvent CDCl3
8 Temperature 295.2
9 Pulse Sequence zg30
10 Experiment 1D
11 Number of Scans 64
12 Receiver Gain 101
13 Relaxation Delay 1.0000
14 Pulse Width 9.4100
15 Spectrometer Frequency 600.14
16 Spectral Width 11904.8
17 Lowest Frequency -2261.3
18 Nucleus 1H
19 Acquired Size 32768
20 Spectral Size 131072
1.00 2.05 1.26 0.76 0.88 0.81 1.22 1.29 4.91 6.08 9.20 1.30 9.19 5.54
9.5 9.0 8.5 8.0 7.5 7.0 6.5 6.0 5.5 5.0 4.5 4.0 3.5 3.0 2.5 2.0 1.5 1.0 0.5 0.0
f1 (ppm)
TBSO
H
O
NBoc
O
H
H

6
***ent*-6**

	Parameter	Value
1	Sample Name	094547-exp-23-ab4138-p-c13.4.fid
2	Origin	Bruker BioSpin GmbH
3	Owner	nmrsu
4	Site	
5	Spectrometer	Avance
6	Author	
7	Solvent	CDCl3
8	Temperature	294.7
9	Pulse Sequence	zgpg30
10	Experiment	1D
11	Number of Scans	12000
12	Receiver Gain	101
13	Relaxation Delay	2.0000
14	Pulse Width	8.0000
15	Spectrometer Frequency	100.63
16	Spectral Width	23809.5
17	Lowest Frequency	-839.2
18	Nucleus	13C
19	Acquired Size	32768
20	Spectral Size	65536

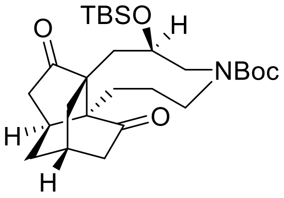

6
***ent*-6**

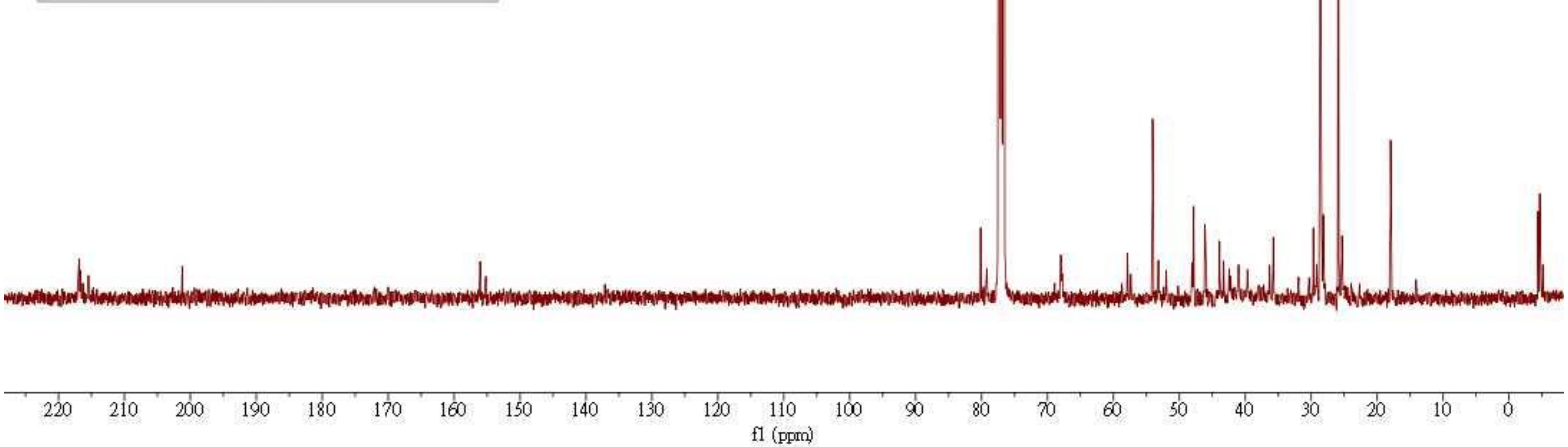

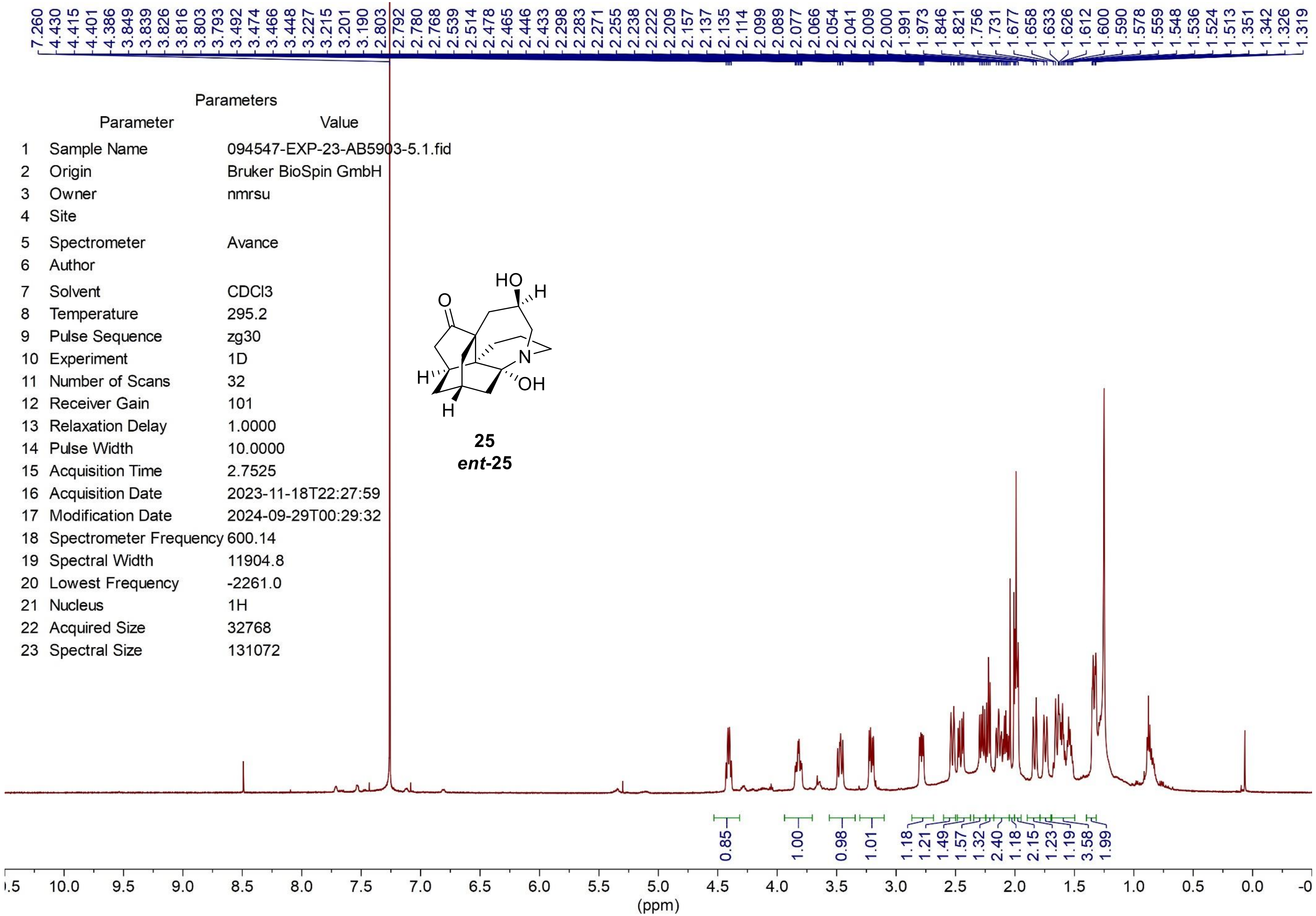

Parameters

	Parameter	Value
1	Sample Name	094547-EXP-23-AB5903-5.1.fid
2	Origin	Bruker BioSpin GmbH
3	Owner	nmrsu
4	Site	
5	Spectrometer	Avance
6	Author	
7	Solvent	CDCl3
8	Temperature	295.2
9	Pulse Sequence	zg30
10	Experiment	1D
11	Number of Scans	32
12	Receiver Gain	101
13	Relaxation Delay	1.0000
14	Pulse Width	10.0000
15	Acquisition Time	2.7525
16	Acquisition Date	2023-11-18T22:27:59
17	Modification Date	2024-09-29T00:29:32
18	Spectrometer Frequency	600.14
19	Spectral Width	11904.8
20	Lowest Frequency	-2261.0
21	Nucleus	1H
22	Acquired Size	32768
23	Spectral Size	131072

25
***ent*-25**

Parameters

	Parameter	Value
1	Sample Name	094547-EXP-23-AB5903-C13-2.1.fid
2	Origin	Bruker BioSpin GmbH
3	Owner	nmrsu
4	Site	
5	Spectrometer	Avance
6	Author	
7	Solvent	CDCl3
8	Temperature	295.2
9	Pulse Sequence	zgpg30
10	Experiment	1D
11	Number of Scans	7000
12	Receiver Gain	90
13	Relaxation Delay	2.0000
14	Pulse Width	12.0000
15	Acquisition Time	0.9175
16	Acquisition Date	2023-11-19T05:57:03
17	Modification Date	2024-07-12T17:05:31
18	Spectrometer Frequency	150.92
19	Spectral Width	35714.3
20	Lowest Frequency	-1264.9
21	Nucleus	13C
22	Acquired Size	32768
23	Spectral Size	65536

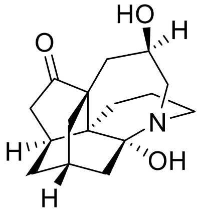

25
***ent*-25**

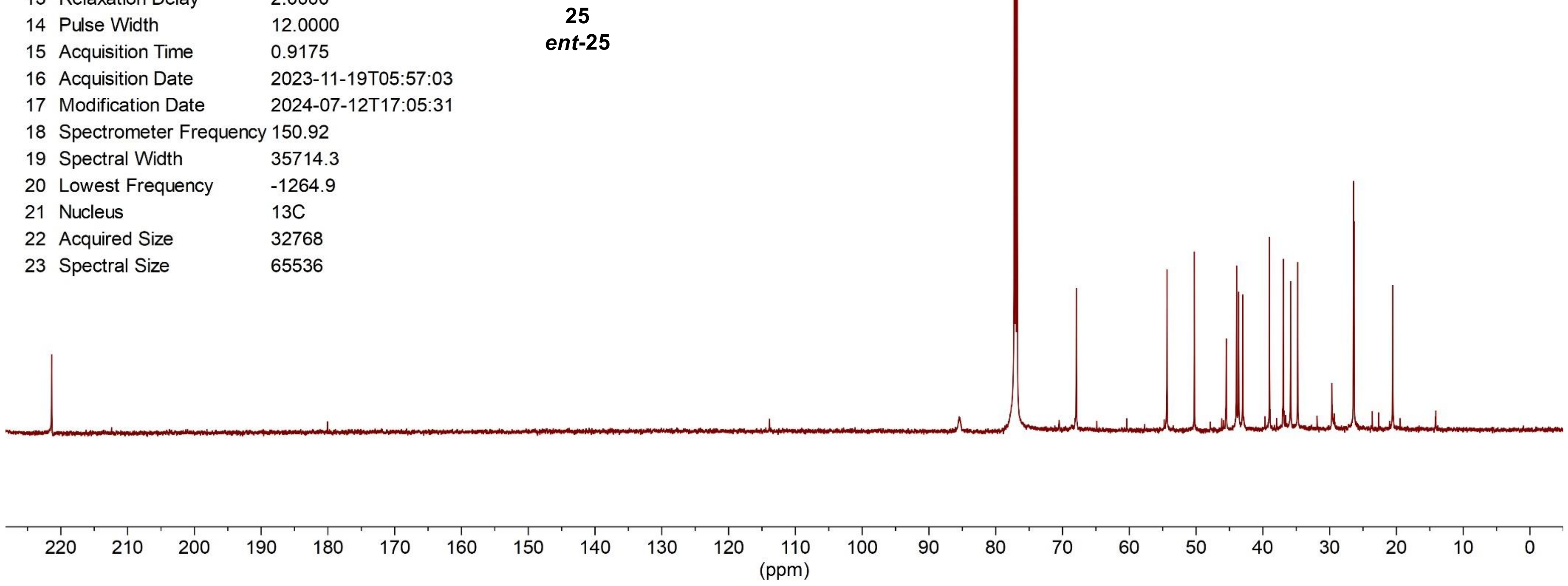

Parameters

	Parameter	Value
1	Sample Name	094547-exp-23-ab5906-k2co3-1.1.fid
2	Origin	Bruker BioSpin GmbH
3	Owner	nmrsu
4	Site	
5	Spectrometer	Avance
6	Author	
7	Solvent	CDCl3
8	Temperature	295.2
9	Pulse Sequence	zg30
10	Experiment	1D
11	Number of Scans	64
12	Receiver Gain	101
13	Relaxation Delay	1.0000
14	Pulse Width	10.0000
15	Acquisition Time	2.7525
16	Acquisition Date	2023-11-21T17:03:06
17	Modification Date	2023-11-21T17:16:18
18	Spectrometer Frequency	600.14
19	Spectral Width	11904.8
20	Lowest Frequency	-2246.5
21	Nucleus	1H
22	Acquired Size	32768
23	Spectral Size	131072

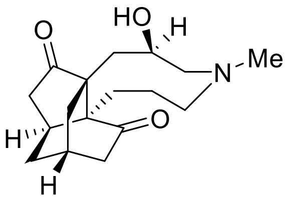

(-)-palhinine B (**1**)
(+)-palhinine B (***ent*-1**)

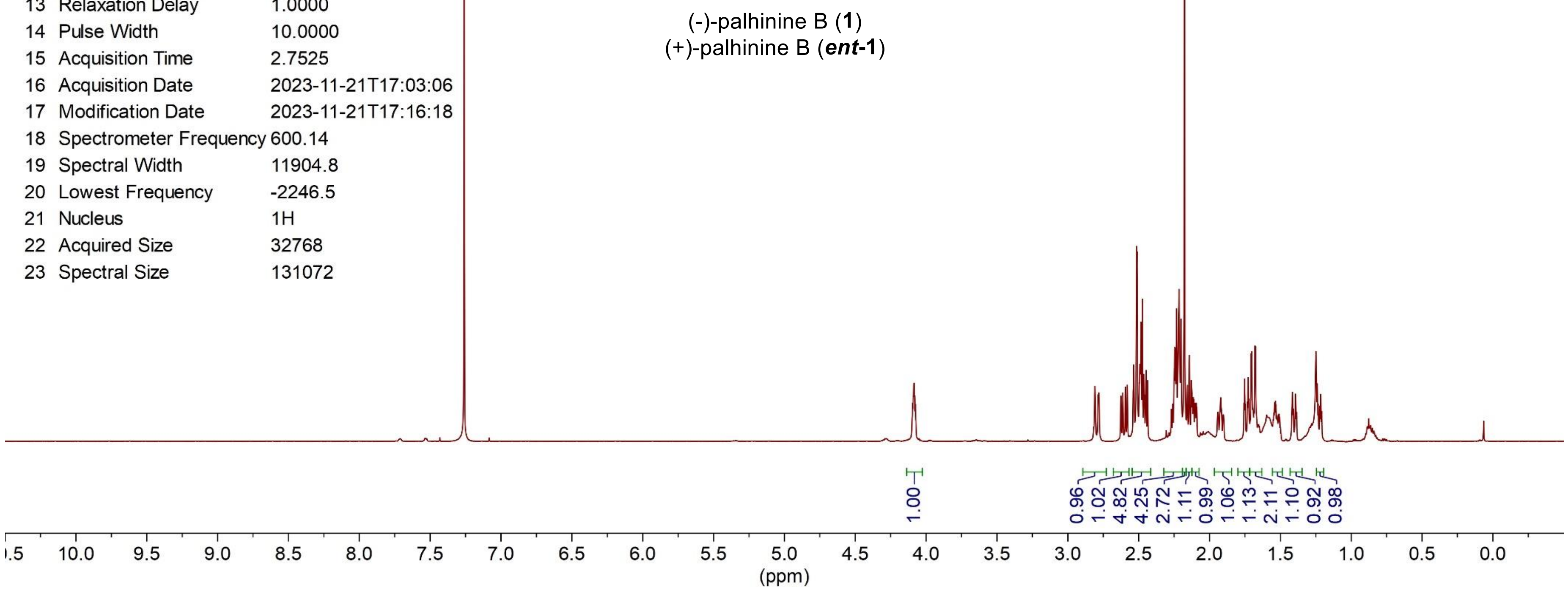

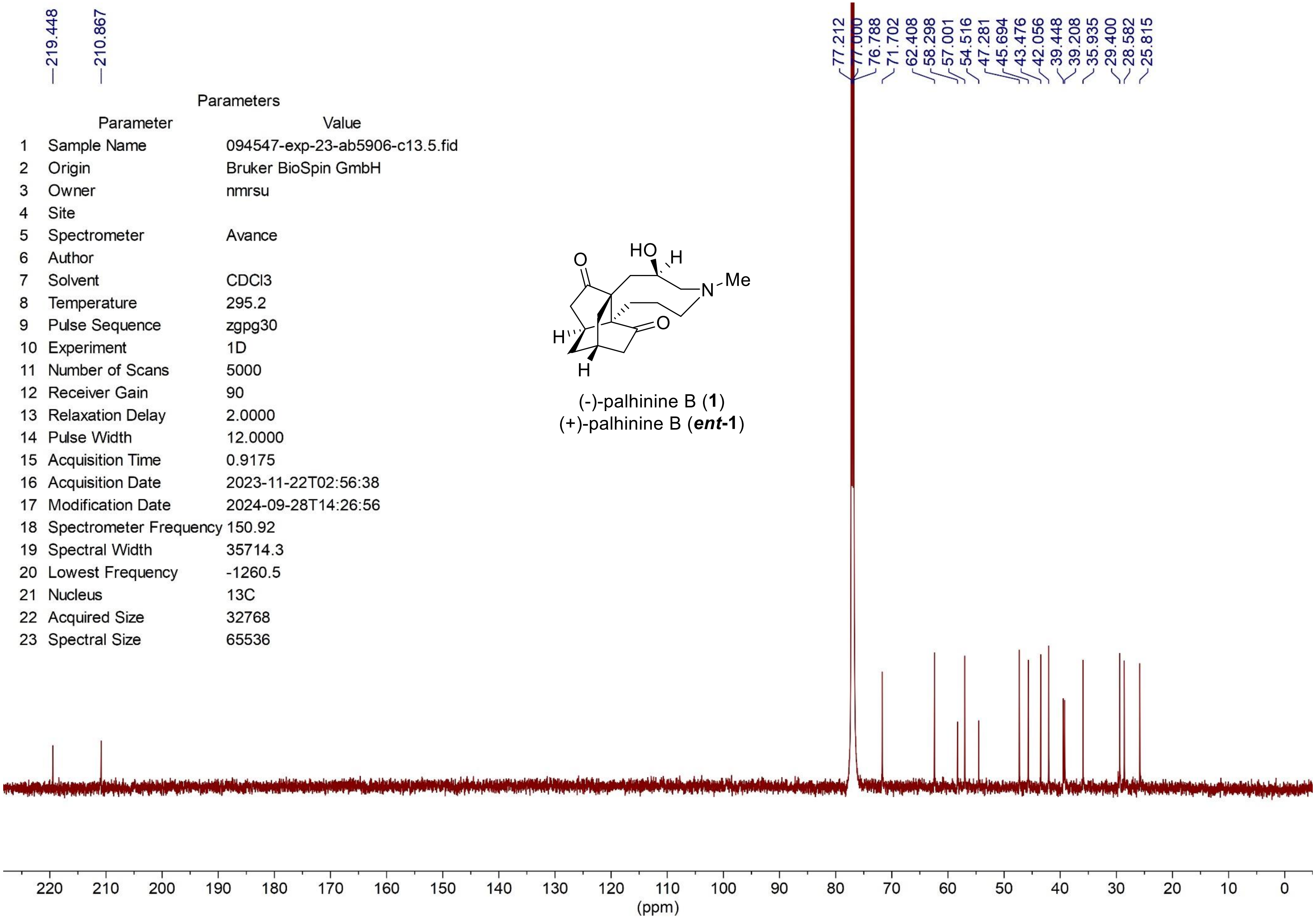

Parameters

	Parameter	Value
1	Sample Name	094547-exp-23-ab5906-c13.5.fid
2	Origin	Bruker BioSpin GmbH
3	Owner	nmrsu
4	Site	
5	Spectrometer	Avance
6	Author	
7	Solvent	CDCl3
8	Temperature	295.2
9	Pulse Sequence	zgpg30
10	Experiment	1D
11	Number of Scans	5000
12	Receiver Gain	90
13	Relaxation Delay	2.0000
14	Pulse Width	12.0000
15	Acquisition Time	0.9175
16	Acquisition Date	2023-11-22T02:56:38
17	Modification Date	2024-09-28T14:26:56
18	Spectrometer Frequency	150.92
19	Spectral Width	35714.3
20	Lowest Frequency	-1260.5
21	Nucleus	13C
22	Acquired Size	32768
23	Spectral Size	65536

(-)-palhinine B (**1**)
(+)-palhinine B (***ent*-1**)

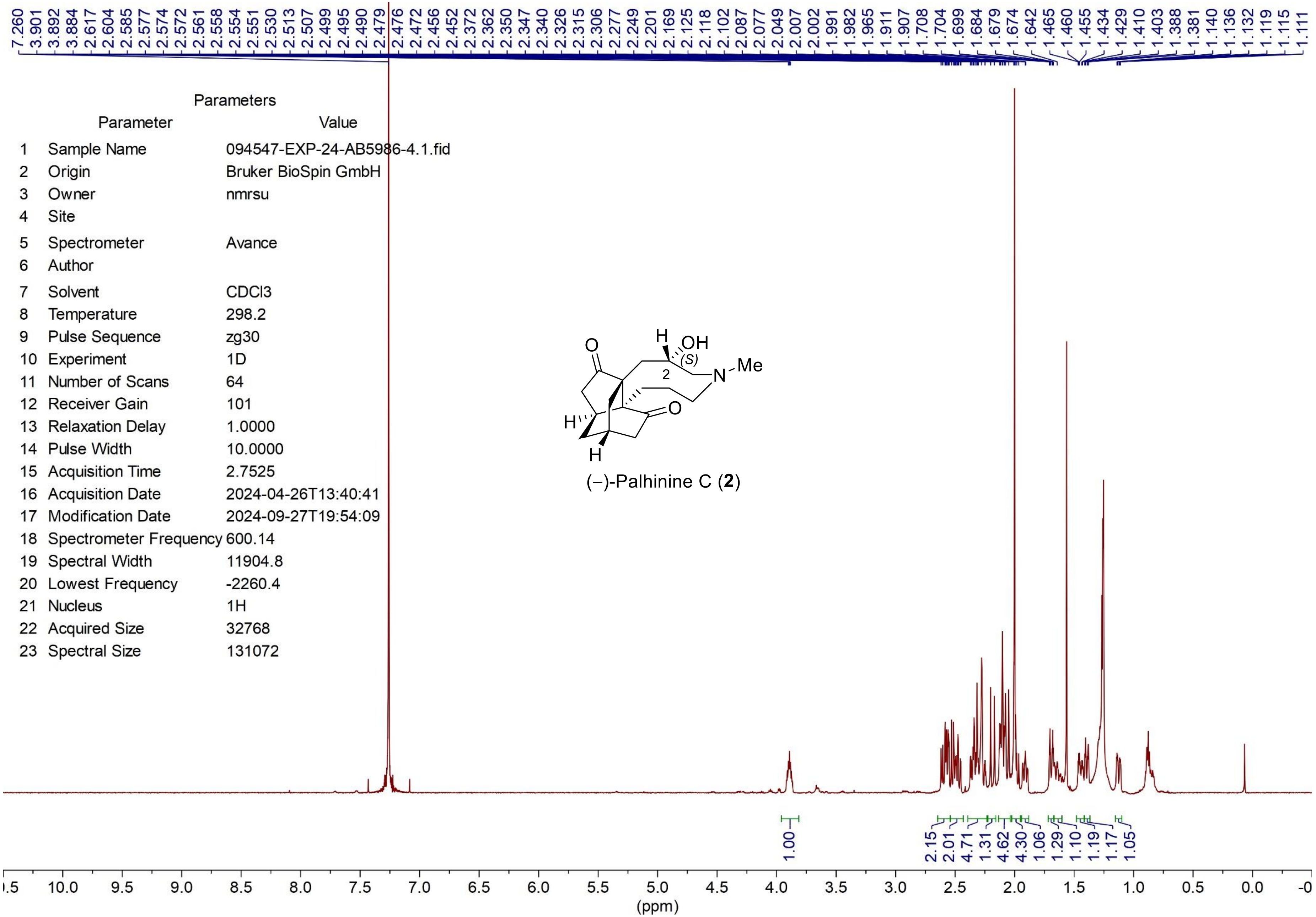

(−)-Palhinine C (**2**)

Parameters

	Parameter	Value
1	Sample Name	094547-EXP-24-AB5986-4.1.fid
2	Origin	Bruker BioSpin GmbH
3	Owner	nmrsu
4	Site	
5	Spectrometer	Avance
6	Author	
7	Solvent	CDCl3
8	Temperature	298.2
9	Pulse Sequence	zg30
10	Experiment	1D
11	Number of Scans	64
12	Receiver Gain	101
13	Relaxation Delay	1.0000
14	Pulse Width	10.0000
15	Acquisition Time	2.7525
16	Acquisition Date	2024-04-26T13:40:41
17	Modification Date	2024-09-27T19:54:09
18	Spectrometer Frequency	600.14
19	Spectral Width	11904.8
20	Lowest Frequency	-2260.4
21	Nucleus	1H
22	Acquired Size	32768
23	Spectral Size	131072

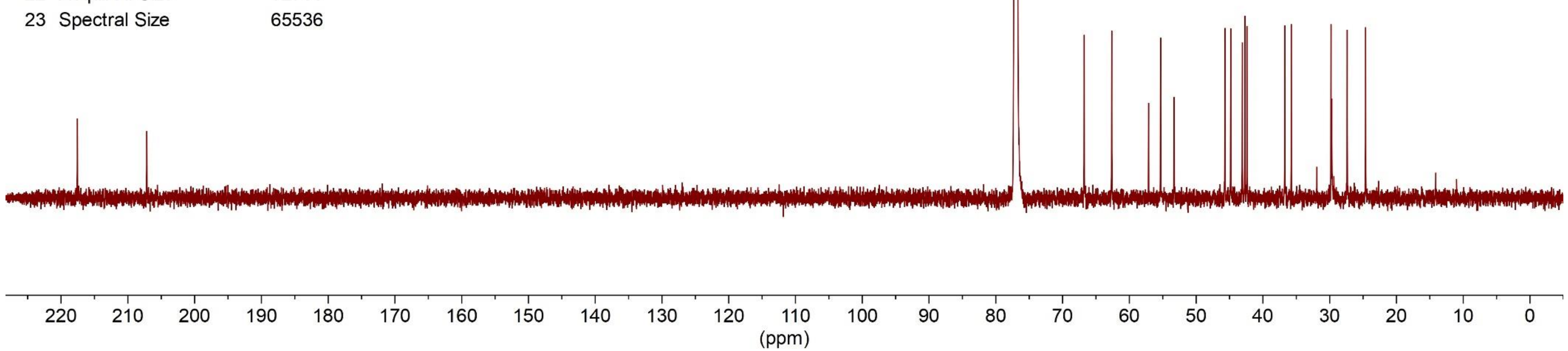

Parameters

	Parameter	Value
1	Sample Name	XGPQgJb6TrS4uEa3lkZevQ.1.fid
2	Origin	Bruker BioSpin GmbH
3	Owner	nmrsu
4	Site	
5	Spectrometer	Avance
6	Author	
7	Solvent	CDCl3
8	Temperature	298.2
9	Pulse Sequence	zgpg30
10	Experiment	1D
11	Number of Scans	8500
12	Receiver Gain	101
13	Relaxation Delay	2.0000
14	Pulse Width	12.0000
15	Acquisition Time	0.9175
16	Acquisition Date	2024-04-26T08:12:14
17	Modification Date	2024-04-26T13:22:19
18	Spectrometer Frequency	150.92
19	Spectral Width	35714.3
20	Lowest Frequency	-1258.7
21	Nucleus	13C
22	Acquired Size	32768
23	Spectral Size	65536

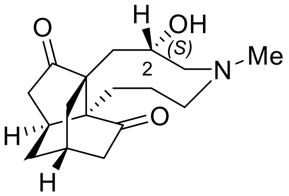

(–)-Palhinine C (**2**)

8. HPLC Spectra

HPLC Spectra of **(9+*ent*-9)-Mixture**

Column: Chiralpak IA	Flow rate: 1.0 mL/min
Solvent: *n*-Hexane/ EtOH = 90/10	Detector: UV 254 nm

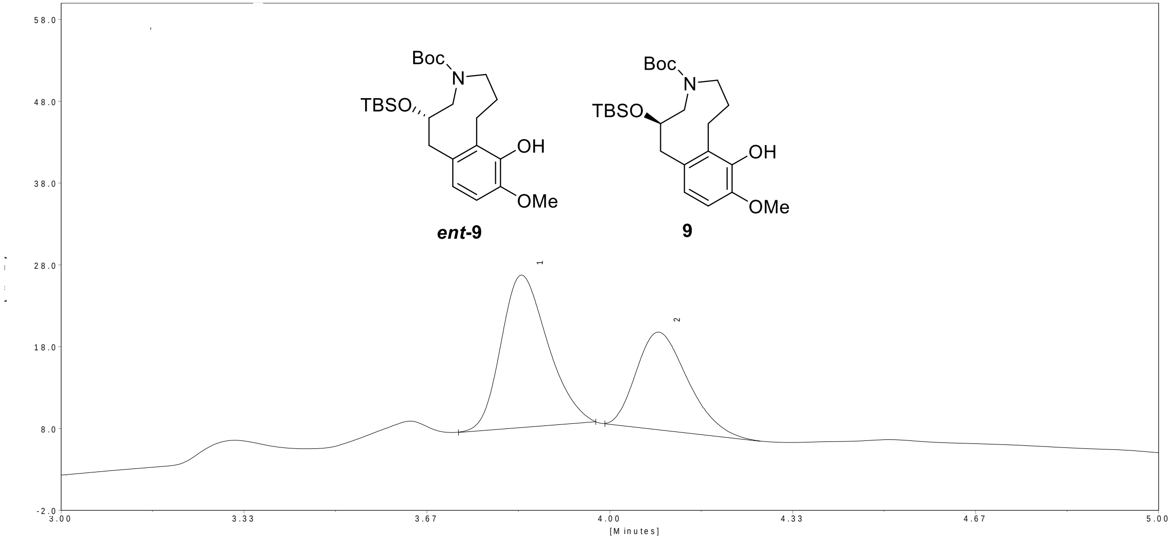

#	Ret. Time (min)	Peak Height (mv)	Area (mv.sec)	Rel. area (%)
1	3.84	18.59	109.70	59.1679
2	4.09	11.84	75.71	40.8321

HPLC Spectra of enantiopure **9**

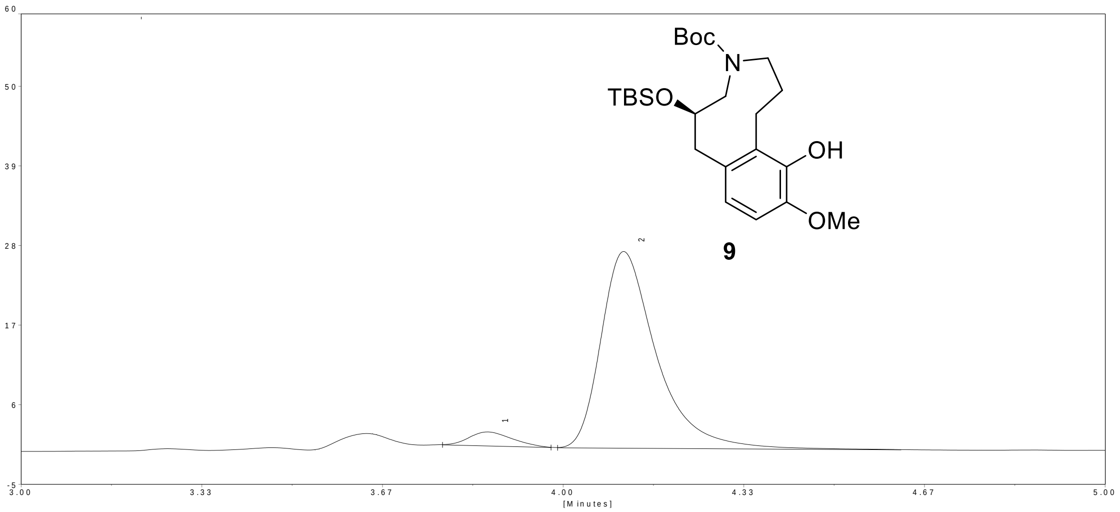

#	Ret. Time (min)	Peak Height (mv)	Area (mv.sec)	Rel. area (%)
1	3.86	1.90	10.32	5.0771
2	4.11	27.15	193.03	94.9229

HPLC Spectra of enantiopure ***ent*-9**

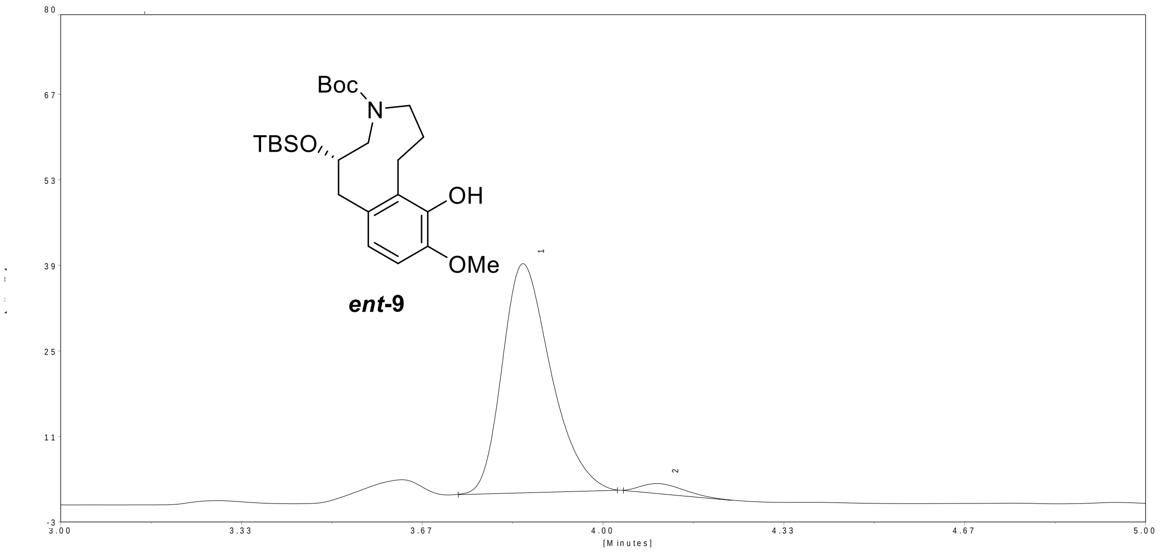

#	Ret. Time (min)	Peak Height (mv)	Area (mv.sec)	Rel. area (%)
1	3.85	37.46	233.78	96.6818
2	4.10	1.56	8.02	3.3182
